# Evolution of energy and nutrient intake in Brazil between 2008–2009 and 2017–2018

**DOI:** 10.11606/s1518-8787.2021055003343

**Published:** 2021-11-22

**Authors:** Eliseu Verly, Dirce Maria Marchioni, Marina Campos Araujo, Eduardo De Carli, Dayan Carvalho Ramos Salles de Oliveira, Edna Massae Yokoo, Rosely Sichieri, Rosangela Alves Pereira

**Affiliations:** I Universidade do Estado do Rio de Janeiro Instituto de Medicina Social Departamento de Epidemiologia Rio de Janeiro RJ Brasil Universidade do Estado do Rio de Janeiro. Instituto de Medicina Social. Departamento de Epidemiologia. Rio de Janeiro, RJ, Brasil; II Fundação Oswaldo Cruz Escola Nacional de Saúde Pública Sérgio Arouca Departamento de Epidemiologia e Métodos Quantitativos em Saúde Rio de Janeiro RJ Brasil Fundação Oswaldo Cruz. Escola Nacional de Saúde Pública Sérgio Arouca. Departamento de Epidemiologia e Métodos Quantitativos em Saúde. Rio de Janeiro, RJ, Brasil; III Universidade de São Paulo Faculdade de Saúde Pública Departamento de Nutrição São Paulo SP Brasil Universidade de São Paulo. Faculdade de Saúde Pública. Departamento de Nutrição. São Paulo, SP, Brasil; IV Universidade Federal Fluminense Instituto de Saúde Coletiva Departamento de Epidemiologia e Bioestatística Niterói RJ Brasil Universidade Federal Fluminense. Instituto de Saúde Coletiva. Departamento de Epidemiologia e Bioestatística. Niterói, RJ, Brasil; V Universidade Federal do Rio de Janeiro Instituto de Nutrição Josué de Castro Departamento de Nutrição Social e Aplicada Rio de Janeiro RJ Brasil Universidade Federal do Rio de Janeiro. Instituto de Nutrição Josué de Castro. Departamento de Nutrição Social e Aplicada. Rio de Janeiro, RJ, Brasil

**Keywords:** Food Consumption, Energy Intake, Deficiency Diseases, epidemiology, Diet, Food, and Nutrition, Diet Surveys

## Abstract

**OBJECTIVE:**

To assess the evolution of energy and nutrient intake and the prevalence of inadequate micronutrients intakes according to sociodemographic characteristics and Brazilian regions.

**METHODS:**

The food consumption of 32,749 individuals from the National Dietary Survey of the Household Budget Survey 2008–2009 was analyzed by two food registries, as well as 44,744 subjects from two 24-hour recalls in 2017–2018. Usual intake and percentage of individuals with consumption below the average recommendation for calcium, magnesium, phosphorus, copper and zinc, vitamins A, C, D, E, thiamine, riboflavin, pyridoxine and cobalamin were estimated. Sodium intake was compared to the reference value to reduce the risk of chronic diseases. Analyses were stratified by sex, age group, region and income.

**RESULTS:**

Mean daily energy intake was 1,753 kcal in 2008–2009 and 1,748 kcal in 2017–2018. The highest prevalence of inadequacy (> 50%) in the two periods were calcium; magnesium; vitamins A, D and E; pyridoxine and, only among adolescents, phosphorus. There was an increase in the prevalence of inadequate vitamin A, riboflavin, cobalamin, magnesium, and zinc among women, and riboflavin among men. The prevalence of inadequacy decreased for thiamine. Sodium intake was excessive in approximately 50% of the population in both periods. The highest variations (about 50%) in the prevalence of inadequacy between the lowest and highest income (< 0.5 minimum wage and > 2 minimum wages per capita) were observed for vitamin B12 and C in both periods. The North and Northeast regions had the highest prevalence of inadequacy.

**CONCLUSION:**

Both surveys found high prevalence of inadequate nutrient intake and excessive sodium intake. The inadequacy varies according to income strata, increasing in the poorest regions of the country.

## INTRODUCTION

The impact of low-quality diets on morbidity and mortality from chronic non-communicable diseases (NCDs) worldwide was greater than that of any other risk factor, including smoking, according to the Global Burden of Disease^1^. Malnutrition in low- and middle-income countries is often characterized by excess energy intake and micronutrient deficiencies, making it challenging to tackle the double burden of diseases generated by the coexistence of obesity and malnutrition, as well as other non-communicable diseases related to diet^2^.

Food security and nutrition are closely linked. Food insecurity is related to poor diet quality, which in turn is linked to manifestations of poor nutrition that increase the risk of malnutrition, overweight, and obesity^2^. In 2015, the countries of the United Nations, including Brazil, committed to the 17 Sustainable Development Goals (SDGs) of the 2030 Agenda. In SDG 2, goal 2.2 is to end all forms of malnutrition^3^.

In Brazil, in 2019, 26% of the population over 18 years old was obese and about 60% overweight^4^. Between 2002–2003 and 2019, the proportion of obese in the population aged 20 years and over more than doubled, going from 12.2% to 26.8%^4^. On the other hand, data from the Household Budget Survey showed that, in 2017–2018, of the 68.9 million households in Brazil, 36.7% (the equivalent of 25.3 million) were with some degree of food insecurity^5^.

The beneficial effects of a healthy eating pattern derive from a cumulative and synergistic effect of nutrients from various food sources^6^. Vitamins and minerals are essential for humans and play an important role in a myriad of metabolic cycles supporting cellular actions. These effects include cardiovascular and bone health, as well as the structure and function of the nervous system^7,8^.

The analysis of a population’s nutritional status and food consumption can follow several complementary approaches, covering nutrient intakes, food groups, and dietary patterns. The assessment of nutrient intake allows identifying the population groups in which there is a risk of inadequacy, both to plan actions and to analyze public policies already implemented, such as food fortification. Recommendations for nutrient intake aim to ensure that most of the population receives amounts meeting physiological needs^9^.

In the National Dietary Survey (INA) carried out in 2008–2009^10^, the first to collect data on individual food consumption in Brazil, high prevalence of inadequacy were observed for vitamin E, vitamin D, calcium, vitamin A, magnesium, and vitamin C in adolescents, adults, and elderly of both sexes. In view of such result, this study aims to assess the evolution of energy and nutrient intake and the prevalence of inadequate micronutrient intakes among Brazilian adolescents, adults and elderly, according to sociodemographic characteristics and Brazilian regions.

## METHODS

Data from two National Dietary Surveys (INA) conducted in 2008–2009 and 2017–2018 were analyzed, as modules of the Household Budget Survey (POF). In both surveys, sampling was defined by clusters, in two stages: in the first, census tracts were drawn; in the second, were drawn households within each sector. Details about the sampling plan are found in other publications^10,11^.

The INA samples corresponded to 24.2% and 34.7% of the households sampled for the POF, totaling 13,569 households and 34,003 individuals in 2008–2009, and 20,112 households and 46,164 individuals in 2017–2018. All individuals aged 10 years or over in the selected households were invited to participate in the individual consumption module. Pregnant and lactating women were not considered in these analyses (n = 1,254 in 2008–2009; n = 1,420 in 2017–2018), totaling 32,749 and 44,744 individuals in the first and second surveys, respectively.

### Data Collection and processing

In the 2008–2009 survey, food consumption data were obtained through a food record filled in by the resident on two non-consecutive days (97% responded the second record). Individuals were instructed to record in detail all the foods and beverages consumed, including the way of preparation, ingredients and quantities, preferably in household measures. Still in the households, research agents passed the information on food consumption to a specific program developed by the Brazilian Institute of Geography and Statistics (IBGE)^11^.

In the 2017–2018 survey, collection was performed using 24-hour recalls (R24h) applied on two non-consecutive days, in the same week in which household expenditure data were obtained (84% responded the second R24h). Subjects were asked to report all foods and beverages consumed the day before the two interviews, including a detailed description of the foods (ingredients, additions, preparation) and quantities. Interviews were structured according to the automated multiple-pass method ^12^, using a software specifically designed for the survey for data collection, with 12 options of items that could be added to the food (olive oil, butter, margarine, mayonnaise, cheese, cream milk, sugar, honey, molasses, ketchup, mustard, and soy sauce).

To analyze the amount and nutritional composition of the additions, the type of reported additions was considered. Fat-based additions (olive oil, butter/margarine, mayonnaise, cheese, and sour cream), when reported, could add a maximum of 20% to the consumption, in grams, of the food to which they were added. The additions of sugar, honey, molasses, ketchup, mustard and soy sauce represented a maximum of 10% of the item’s consumption. That is, if ketchup and mustard were added to a sandwich, each addition represents 5% of the sandwich’s weight. Details on data collection, training, quality control and imputation can be found in the IBGE official publication^10^.

### Nutritional Composition of Foods

To convert the reported foods into amounts of energy and nutrients, the Brazilian Table of Food Composition (TBCA) v.7.0 was used in both surveys^13^. One should mention, however, the nutritional composition table changed between surveys. The justification for this change is due to the fact the TBCA describes nutritional data on Brazilian foods with the reliability ensured by the International Food Data System Network, of the Food and Agriculture Organization of the United Nations (FAO), which determines guidelines and criteria to be used in the generation, compilation, and use of food composition data^14^. Thus, to reduce the possibility of divergences in trend assessments and to allow for comparability, the TBCA was used in the energy and nutrient estimates of both surveys.

### Statistical Analysis

Usual intake distribution of each nutrient was estimated using the National Cancer Institute (NCI) method, which applies a mixed-effect model after Box-Cox transformation of intake data^15^. Parameters (population mean, inter- and within-person variances, effects of the age group and sex variables, income and region, and the lambda of the Box-Cox transformation) are estimated and then used to create usual consumption distributions using Monte Carlo simulation.

Usual intake distributions were estimated for each age-sex group for which there are nutrient recommendation established. Prevalence of inadequacy was estimated using the EAR (estimated average requirement) method as a cutoff point, which represents the percentage of individuals with an intake below the estimated mean value of need for each age-sex group^16^.

Sodium intake was compared to the reference value for reducing the risk of chronic diseases (chronic disease risk reduction intakes), as proposed in the latest review of reference values for sodium by the US Institute of Medicine^17^. From this reference, the percentage of population that should reduce sodium consumption to reduce the risk of chronic diseases is calculated. Considering the incompatibility between the preformed niacin composition in foods, available in the TBCA, with the EAR values expressed in niacin equivalents, prevalence of inadequacy was not calculated for this nutrient.

The prevalence of inadequacy was estimated for each age-sex group for which there are EAR established (9 to 13 years old, 14 to 18, 19 to 30, 31 to 50, 51 to 70, and 70 or more). Then, averages of the prevalence weighted by the expanded sample size of each age group were calculated, stratified into age groups corresponding to the life cycles: adolescents (10–18 years old), adults (19–59 years old), and elderly (60 years or more). The same procedure was used to compute overall prevalence by income strata and region of the country. For the income strata, per capita income of the families was used: less than 0.5, between 0.5 and 1, between 1 and 2, and greater than 2 minimum wages per capita. The minimum wage values on the reference dates of the surveys were: R$ 415.00 (January 15, 2009) and R$ 954.00 (January 15, 2018).

The 95% confidence intervals (95%CI) were calculated for the means and the prevalence of inadequacy using standard error corrected by sample design, estimated by the balanced repeated replication (BRR) technique, used in the NCI method. The 95%CI was used to compare the prevalence of inadequacy between sexes, age groups, income, regions of the country, and the two periods evaluated. Analyses were performed using the SAS software version 9.4.

## RESULTS

The mean (95%CI) daily energy intake were similar in the two surveys: 1,753 kcal (1,734–1,772) in 2008–2009 and 1,748 kcal (1,732–1,763) in 2017–2018. Among men, the mean caloric intake in 2008–2009 for adolescents, adults, and the elderly was 1,996 kcal (1,945–2,047), 1,969 kcal (1,940–1,998) and 1,680 kcal (1,633–1,726), respectively; and 1,969 kcal (1,927–2010), 2,018 kcal (1,993–2,044) and 1,708 kcal (1,669–1,747) in 2017–2018. Among women, the values were 1,753 kcal (1,716–1,798), 1,549 kcal (1,539–1,582) and 1,410 kcal (1,370–1,449) in 2008–2009; and 1,674 kcal (1,633–1,715), 1,549 kcal (1,530–1,568) and 1,409 kcal (1,381–1,437) in 2017–2018 for the same age groups, respectively.

A lower energy intake was observed among women and with increasing age in both surveys, with the exception of adult men, whose means did not differ from those of adolescents. However, in both surveys, adult men, when compared to other age groups and women, had the highest average intake for most micronutrients. There was a slight reduction (< 23%) among surveys in average intakes for most micronutrients, while increases in average intakes (4%–10%) occurred only among male adults for thiamine, and among adults of both sexes for vitamin E (Table 1).

**Table 1:** Mean (95%CI) micronutrient intakes among adolescents, adults, and the elderly in the National Dietary Surveys, 2008–2009 and 2017–2018.

Nutrients		Adolescents		Adults		Elderly	
		2008–2009	2017–2018	2008–2009	2017–2018	2008–2009	2017–2018
Men							
	Calcium (mg)	470.1 (455.5–484.7)	457.1 (436.5–477.6)	483.7 (469.6–497.7)	472.7 (459.0–486.4)	469.3 (453.3–485.3)	446.3 (434.8–457.7)
	Magnesium (mg)	285.8 (280.3–291.2)	273.3 (266.6–280.0)	310.0 (307.2–312.8)	304.7 (301.5–307.9)	285.3 (277.6–293.0)	275.7 (270.6–280.8)
	Phosphorus (mg)	1,094.6 (1,074.7–1,114.4)	1,081.9 (1,067.0–1096.8)	1167.2 (1147.3–1187.0)	1,153.4 (1,144.9–1,161.8)	1,036.6 (1,022.7–1,050.5)	977.1 (959.8–994.4)
	Sodium (mg)	2,784.4 (2,706.2–2,862.5)	2,805.5 (2,745.6–2,865.5)	2,910.9 (2,866.9–2,954.8)	2,961.0 (2,922.1–2,999.8)	2,462.1 (2,369.4–2,554.7)	2,472.8 (2,396.8–2,548.8)
	Potassium (mg)	2,387.5 (2,325.5–2,449.4)	2,218.2 (2,166.8–2,269.6)	2,607.3 (2,571.4–2,643.1)	2,492.4 (2,459.6–2,525.3)	2,484.7 (2,412.2–2,557.2)	2,275.7 (2,229.3–2,322.1)
	Copper (mg)	1.4 (1.4–1.5)	1.4 (1.4–1.5)	1.6 (1.5–1.6)	1.6 (1.5–1.6)	1.4 (1.3–1.5)	1.4 (1.4–1.4)
	Zinc (mg)	11.8 (11.3–12.2)	11.5 (11.1–11.9)	12.9 (12.7–13.1)	12.7 (12.5–12.8)	11.5 (11.2–11.9)	10.9 (10.8–11.0)
	Vitamin A (mcg)^a^	376.3 (343.2–409.3)	324.6 (307.6–341.7)	378.5 (359.3–397.8)	320.1 (306.6–333.6)	376.5 (357–396.0)	342.4 (324.7–360.0)
	Thiamine (mg)	1.0 (1.0–1.1)	1.1 (1.1–1.2)	1.0 (0.9–1.0)	1.1 (1.1–1.1)	0.9 (0.8–0.9)	0.9 (0.9–1.0)
	Riboflavin (mg)	1.3 (1.3–1.4)	1.2 (1.2–1.3)	1.2 (1.2–1.3)	1.1 (1.1–1.1)	1.2 (1.2–1.2)	1.1 (1.1–1.1)
	Pyridoxine (mg)	0.8 (0.8–0.8)	0.8 (0.8–0.9)	0.8 (0.7–0.8)	0.8 (0.8–0.8)	0.6 (0.6–0.7)	0.7 (0.7–0.7)
	Cobalamin (mcg)	5.0 (4.7–5.3)	4.1 (3.9–4.4)	5.6 (5.5–5.8)	4.6 (4.5–4.7)	5.2 (4.9–5.4)	3.9 (3.8–4.0)
	Vitamin D (mcg)^b^	2.4 (2.3–2.5)	2.0 (1.9–2.0)	2.2 (2.0–2.3)	1.7 (1.6–1.7)	2.0 (1.9–2.1)	1.5 (1.5–1.5)
	Vitamin E (mg)^c^	6.6 (6.4–6.8)	6.7 (6.6–6.9)	7.0 (6.9–7.0)	7.3 (7.2–7.5)	6.2 (6.1–6.4)	6.4 (6.3–6.6)
	Vitamin C (mg)	124.0 (119.2–128.8)	117.1 (108.2–126.0)	131.4 (127.9–134.9)	119.8 (117.2–122.4)	126.0 (118.9–133.0)	116.5 (107.0–126.0)
	Niacin (mg)^d^	15.6 (14.9–16.2)	17.3 (16.3–18.4)	18.6 (18.0–19.2)	19.7 (19.1–20.3)	15.5 (14.6–16.3)	15.8 (15.0–16.6)
Women							
	Calcium (mg)	447.2 (435.4–458.9)	406.6 (379.2–434.1)	429.4 (419.7–439.2)	398.7 (394.1–403.2)	451.3 (425.5–477.1)	429.3 (418.5–440.0)
	Magnesium (mg)	244.3 (238.9–249.7)	229.7 (224.2–235.2)	240.8 (238.9–242.7)	232.8 (229.1–236.6)	232.4 (229.9–234.9)	225.5 (221.9–229.1)
	Phosphorus (mg)	977.1 (962.4–991.7)	924.6 (889.9–959.3)	928.9 (923.1–934.7)	878.9 (861.3–896.6)	877.3 (866.2–888.3)	811.2 (802.3–820.1)
	Sodium (mg)	2,408.6 (2,344.3–2472.8)	2,349.7 (2,293.8–2,405.7)	2,227 (2,193.6–2,260.5)	2,180.2 (2,151.7–2,208.7)	1,983.3 (1,917.3–2,049.3)	1,921.2 (1,878.0–1,964.4)
	Potassium (mg)	2,100.3 (2,052.8–2147.7)	1,906.2 (1,863.0–1,949.4)	2,140.5 (2,111.4–2,169.6)	1,972.3 (1,948.7–1,996.0)	2,109.0 (2,057.3–2,160.8)	1,948.3 (1,911.5–1,985.1)
	Copper (mg)	1.2 (1.2–1.3)	1.2 (1.2–1.2)	1.2 (1.2–1.2)	1.2 (1.2–1.2)	1.1 (1.1–1.2)	1.2 (1.1–1.2)
	Zinc (mg)	10.2 (9.9–10.5)	9.6 (9.4–9.8)	10.1 (10.0–10.2)	9.5 (9.3–9.7)	9.5 (9.2–9.8)	8.6 (8.4–8.8)
	Vitamin A (mcg)^a^	400.2 (359.9–440.5)	319.6 (289.5–349.8)	410.4 (384.6–436.1)	342.2 (325.9–358.6)	450.8 (429.3–472.4)	412 (399.8–424.3)
	Thiamine (mg)	1.0 (0.9–1.0)	1.0 (1.0–1.0)	0.8 (0.8–0.9)	0.9 (0.8–0.9)	0.8 (0.7–0.8)	0.8 (0.8–0.8)
	Riboflavin (mg)	1.2 (1.2–1.3)	1.1 (1.0–1.1)	1.2 (1.1–1.2)	1.0 (0.9–1.0)	1.2 (1.1–1.2)	1.0 (1.0–1.0)
	Pyridoxine (mg)	0.8 (0.7–0.8)	0.7 (0.7–0.7)	0.7 (0.6–0.7)	0.6 (0.6–0.6)	0.6 (0.5–0.6)	0.6 (0.6–0.6)
	Cobalamin (mcg)	4.7 (4.2–5.3)	3.8 (3.5–4.0)	4.6 (4.5–4.8)	3.6 (3.5–3.7)	4.4 (4.3–4.6)	3.3 (3.3–3.4)
	Vitamin D (mcg)^b^	2.2 (2.1–2.4)	1.8 (1.6–1.9)	1.8 (1.8–1.9)	1.4 (1.4–1.5)	1.8 (1.6–2.0)	1.3 (1.3–1.3)
	Vitamin E (mg)^c^	5.8 (5.6–6.0)	5.8 (5.4–6.1)	5.5 (5.4–5.6)	5.9 (5.7–6.1)	5.3 (5.3–5.4)	5.5 (5.3–5.6)
	Vitamin C (mg)	128.0 (120.7–135.2)	126.0 (117.6–134.4)	134.6 (131.7–137.6)	120.6 (117.6–123.6)	133.5 (122.4–144.7)	128.9 (121.2–136.6)
	Niacin (mg)^d^	13.8 (13.2–14.3)	14.6 (13.7–15.4)	13.7 (13.4–14.0)	14.4 (14.0–14.8)	12.6 (12.0–13.2)	13.0 (12.4–13.5)

a Retinol activity equivalent (RAE).

b Ergocalciterol (D2) + cholecalciferol (D3).

c Total alpha-tocopherol.

d Preformed Niacin.

Inadequate intakes above 50% of the individuals were observed for pyridoxine, vitamin A, and magnesium in all age groups and, in particular, for phosphorus among adolescents, thiamine among adults and the elderly in both surveys and, in 2017–2018, riboflavin among elderly men and adults of both sexes. Calcium, vitamin D, and vitamin E showed the greatest inadequacies in both surveys (> 85%) (Table 2).

**Table 2:** Prevalence (95%CI) of inadequate micronutrient intakes among adolescents, adults, and the elderly in the National Dietary Surveys,2008–2009 and 2017–2018.

Nutrients		Adolescents		Adults		Elderly	
		2008–2009	2017–2018	2008–2009	2017–2018	2008–2009	2017–2018
Men							
	Calcium (mg)	97.4 (96.9–97.9)	98.1 (97.9–98.4)	89.1 (88.0–90.2)	91.0 (90.1–91.8)	92.4 (91.0–93.8)	94.4 (93.6–95.1)
	Magnesium (mg)	50.2 (48.5–52.0)	54.2 (51.2–57.2)	66.1 (65.2–67.1)	69.2 (67.9–70.5)	77.0 (74.4–79.5)	80.5 (78.7–82.3)
	Phosphorus (mg)	50.3 (47.8–52.8)	51.8 (49.9–53.7)	2.8 (2.3–3.4)	2.1 (1.8–2.5)	6.5 (5.7–7.4)	6.9 (6.4–7.5)
	Copper (mg)	3.1 (2.7–3.6)	2.1 (1.7–2.4)	3.7 (3.2–4.1)	2.4 (2.1–2.7)	6.4 (4.7–8.1)	4.5 (4.0–5.0)
	Zinc (mg)	16.5 (13.9–19.0)	17.5 (14.7–20.3)	22.4 (21.1–23.6)	23.5 (22.4–24.7)	33.5 (30.8–36.1)	39.0 (37.7–40.2)
	Vitamin A (mcg)^a^	78.8 (75.4–82.3)	83.5 (81.1–85.9)	84.7 (82.9–86.4)	89.3 (88.4–90.2)	84.7 (82.9–86.6)	87.5 (86.1–89.0)
	Thiamine (mg)	41.1 (38.1–44.1)	32.0 (29.7–34.4)	59.8 (56.8–62.8)	51.8 (50.9–52.7)	70.0 (68.1–72.0)	63.6 (61.8–65.4)
	Riboflavin (mg)	31.4 (26.7–36.0)	36.6 (34.3–38.9)	45.9 (43.9–47.8)	55.0 (54.1–55.9)	47.9 (45.2–50.5)	57.9 (56.9–58.8)
	Pyridoxine (mg)	70.2 (68.6–71.8)	68.8 (65.7–72.0)	81.5 (77.9–85.0)	82.3 (80.5–84.0)	95.9 (94.5–97.2)	95.0 (94.3–95.7)
	Cobalamin (mcg)	6.2 (4.3–8.1)	7.7 (6.4–9.1)	5.5 (4.3–6.6)	7.2 (6.2–8.1)	7.9 (6.2–9.6)	13.3 (11.9–14.7)
	Vitamin D (mcg)^b^	99.4 (99.2–99.6)	99.8 (99.8–99.9)	99.6 (99.4–99.8)	99.9 (99.9–100)	99.7 (99.6–99.9)	100 (99.9–100.0)
	Vitamin E (mg)^c^	90.6 (89.5–91.7)	89.1 (87.4–90.8)	93.6 (93.0–94.1)	91.6 (90.4–92.8)	96.1 (95.3–96.8)	95.3 (94.2–96.3)
	Vitamin C (mg)	33.2 (31.7–34.6)	34.5 (30.0–38.9)	43.7 (42.8–44.6)	47.5 (45.8–49.2)	45.3 (42.8–47.8)	48.7 (46.2–51.3)
Women							
	Calcium (mg)	98.0 (97.6–98.3)	99.0 (98.5–99.4)	93.7 (93.1–94.3)	96.1 (95.8–96.5)	97.5 (96.9–98.1)	98.5 (98.3–98.6)
	Magnesium (mg)	57.8 (54.2–61.4)	64.7 (62.5–66.9)	64.1 (63.3–64.9)	68.8 (66.9–70.7)	69.1 (68.0–70.1)	73.0 (71.8–74.1)
	Phosphorus (mg)	64.0 (62.2–65.7)	71.1 (66.8–75.3)	11.5 (10.8–12.2)	12.8 (12.0–13.6)	15.0 (14.1–15.9)	18.7 (17.2–20.1)
	Copper (mg)	7.3 (6.3–8.3)	6.6 (5.9–7.2)	13.1 (12.0–14.3)	10.6 (9.9–11.3)	15.0 (13.8–16.3)	11.9 (10.3–13.5)
	Zinc (mg)	21.1 (18.8–23.4)	25.6 (23.7–27.5)	18.1 (17.1–19.1)	22.2 (20.7–23.8)	23.0 (20.2–25.7)	31.4 (29.2–33.7)
	Vitamin A (mcg)^a^	69.2 (64.9–73.4)	78.9 (75.3–82.5)	72.4 (69.7–75.1)	80.1 (78.5–81.6)	68.0 (65.7–70.3)	72.3 (71.1–73.6)
	Thiamine (mg)	42.5 (40.2–44.8)	40.8 (39.3–42.3)	63.5 (62.4–64.6)	61.8 (60.6–63.1)	70.2 (67.3–73.1)	66.5 (65.8–67.2)
	Riboflavin (mg)	27.1 (22.2–31.9)	40.2 (34.4–46.1)	36.6 (34.5–38.6)	51.4 (50.0–52.8)	36.6 (32.6–40.6)	46.2 (44.7–47.8)
	Pyridoxine (mg)	71.2 (68.6–73.8)	75.8 (72.6–78.9)	89.5 (87.8–91.3)	91.8 (91.1–92.5)	96.4 (95.1–97.8)	96.7 (96.2–97.2)
	Cobalamin (mcg)	7.6 (4.4–10.8)	11.4 (7.6–15.3)	10.9 (9.2–12.5)	17.1 (15.5–18.8)	12.6 (11.1–14.0)	21.3 (18.2–24.4)
	Vitamin D (mcg)^b^	99.5 (99.3–99.6)	99.9 (99.8–100.0)	99.8 (99.7–99.9)	100.0 (99.9–100)	99.8 (99.6–100.0)	100.0 (100.0–100.0)
	Vitamin E (mg)^c^	94.1 (93.2–94.9)	93.9 (92.1–95.6)	98.1 (97.9–98.4)	97.0 (96.1–97.8)	98.4 (98.2–98.6)	98.0 (97.4–98.6)
	Vitamin C (mg)	29 (26.3–31.7)	29.4 (25.7–33.1)	34.4 (33.5–35.2)	38.5 (37.1–39.9)	34.7 (31.6–37.9)	35.7 (32.9–38.6)

a Retinol activity equivalent (RAE).

b Ergocalciterol (D2) + cholecalciferol (D3).

c Total alpha-tocopherol.

The prevalence of inadequacy for most micronutrients was higher in 2017–2018 than in 2008–2009. The most important differences (> 10%) found in the female population were for riboflavin and zinc in all age groups, and riboflavin between adult and elderly men. Conversely, thiamine among elderly women and men of all ages, and copper among adult and elderly women and among adolescent and adult men were the only nutrients that showed a slight reduction (< 10 percentage points) in the prevalence of inadequacy over the period (Table 2).

In 2008–2009, the percentage of individuals whose sodium intake should be lowered to reduce the risk of chronic disease was, among men, 75% (72%–78%) in adolescents, 71% (70%–72%) in adults, and 52% (47%–56%) in the elderly. Among women, these percentages were 61% (59%–63%), 41% (39%–43%), and 29% (28%–30%), respectively. In 2017–2018, the percentages were, for men, 78% (76%–80%) in adolescents, 74% (73%–75%) in adults, and 52% (50%–54%) in the elderly. Among women, they were 59% (56%–61%), 39% (36%–41%), and 25% (24%–27%), respectively.

Both in 2008–2009 (Tables 3 and 4) and 2017–2018 (Tables 5 and 6), there was a reduction in the prevalence of inadequacy of micronutrients with the increase in average per capita income. In the last survey, except for copper, magnesium, and vitamin D among the elderly, and vitamin E among adolescent women, significant differences were observed between the lowest and highest income levels (< 0.5 *vs* > 2 minimum wages), being the most expressive (> 15 percentage points) for vitamin C, vitamin A, riboflavin, and thiamine at all ages, phosphorus and pyridoxine among adolescents, and calcium among adult men (Tables 5 and 6).

**Table 3:** Prevalence (95%CI) of inadequate micronutrient intakes among men according to per capita household income in the National Dietary Survey 2008–2009.

Nutrients	Age group	Per capita income			
		< 0.5 SM	0.5–1 MW	1–2 MW	> 2 MW
		% (95%CI)	% (95%CI)	% (95%CI)	% (95%CI)
Calcium (mg)					
	Adolescents	99.3 (98.9–99.6)	98.5 (98.3–98.8)	97.2 (96.4–98.0)	92.4 (90.9–94.0)
	Adults	96.7 (96.1–97.3)	94.2 (93.8–94.7)	90.7 (89.6–91.8)	80.7 (78.5–83.0)
	Elderly	98.4 (97.9–98.9)	96.8 (96.1–97.4)	95.1 (94.5–95.7)	87.4 (85.1–89.7)
Magnesium (mg)					
	Adolescents	50.0 (45.2–54.8)	51.0 (48.1–53.9)	48.1 (45.6–50.5)	53.3 (50.3–56.2)
	Adults	71.1 (69.1–73.1)	65.3 (62.8–67.7)	63.6 (62.5–64.7)	66.6 (64.4–68.9)
	Elderly	81.4 (77.0–85.9)	76.4 (72.4–80.4)	76.1 (72.3–79.9)	77.6 (76.1–79.0)
Phosphorus (mg)					
	Adolescents	62.2 (60.0–64.3)	50.3 (48.1–52.6)	45.4 (42.6–48.1)	36.1 (33.5–38.7)
	Adults	5.6 (4.9–6.3)	3.3 (2.4–4.2)	2.5 (2.1–3.0)	1.5 (1.1–1.9)
	Elderly	13.4 (10.8–16.1)	8.2 (6.7–9.7)	7.2 (5.6–8.7)	4.1 (3.6–4.6)
Copper (mg)					
	Adolescents	3.6 (2.7–4.4)	2.9 (2.5–3.4)	2.7 (2.1–3.3)	3.5 (3.0–3.9)
	Adults	4.6 (3.7–5.5)	3.3 (2.9–3.8)	3.3 (2.7–4.0)	3.7 (3.1–4.4)
	Elderly	8.4 (5.5–11.4)	6.0 (4.2–7.7)	6.4 (4.0–8.8)	6.3 (5.0–7.5)
Zinc (mg)					
	Adolescents	21.4 (16.2–26.6)	15.3 (13.9–16.7)	14.0 (12.1–16.0)	14.0 (12.6–15.4)
	Adults	30.3 (28.2–32.4)	22.1 (19.7–24.5)	20.8 (19.7–22.0)	20.1 (18.2–22.0)
	Elderly	44.4 (40.4–48.4)	34.0 (31.3–36.7)	34.3 (31.6–37.0)	31.0 (26.6–35.4)
Vitamin A (mcg)^a^					
	Adolescents	87.0 (82.1–91.9)	81.6 (79.0–84.3)	76.0 (73.8–78.2)	61.6 (58.7–64.6)
	Adults	95.2 (93.4–96.9)	90.2 (88.7–91.7)	86.6 (84.7–88.4)	73.9 (70.9–76.9)
	Elderly	96.1 (93.9–98.2)	92.3 (90.6–93.9)	88.8 (87.5–90.2)	77.1 (74.3–79.9)
Thiamine (mg)					
	Adolescents	52.8 (45.3–60.3)	41.6 (38.6–44.6)	34.5 (32.6–36.5)	28.5 (25.9–31.1)
	Adults	78.0 (74.9–81.0)	64.4 (60.9–67.8)	57.9 (55.8–60.0)	49.6 (43.9–55.2)
	Elderly	87.8 (84.4–91.3)	77.2 (74.4–80.1)	72.0 (68.0–76.0)	62.8 (59.9–65.8)
Riboflavin (mg)					
	Adolescents	42.8 (35.6–50.0)	30.6 (26.3–34.9)	25.5 (22.8–28.3)	20.1 (17.7–22.6)
	Adults	65.7 (61.1–70.2)	49.3 (46.9–51.7)	43.8 (42.3–45.2)	35.7 (32.9–38.6)
	Elderly	71.3 (68.1–74.5)	54.9 (50.9–58.9)	49.9 (47.7–52.2)	39.8 (36.1–43.6)
Pyridoxine (mg)					
	Adolescents	81.5 (78.1–84.8)	71.3 (69.1–73.4)	64.6 (62.3–67.0)	55.2 (52.7–57.6)
	Adults	92.9 (91.1–94.6)	85.1 (82.1–88.1)	81.4 (77.9–84.8)	73.6 (68.2–79.0)
	Elderly	99.5 (99.2–99.8)	97.8 (97.2–98.5)	97.0 (96.3–97.8)	94.0 (92.5–95.6)
Cobalamin (mcg)					
	Adolescents	7.6 (5.2–9.9)	6.6 (4.7–8.5)	5.4 (3.9–7.0)	3.8 (2.6–4.9)
	Adults	8.1 (6–10.3)	6.4 (5.1–7.8)	5.5 (4.4–6.7)	3.5 (2.5–4.6)
	Elderly	12.8 (10.9–14.6)	9.8 (7.7–12)	9.2 (7.7–10.7)	5.3 (3.5–7.1)
Vitamin D (mcg)^b^					
	Adolescents	99.5 (99.3–99.7)	99.5 (99.4–99.6)	99.3 (99.1–99.6)	98.9 (98.5–99.2)
	Adults	99.7 (99.6–99.9)	99.7 (99.5–99.8)	99.7 (99.5–99.8)	99.3 (99.0–99.7)
	Elderly	99.9 (99.7–100)	99.8 (99.7–100)	99.7 (99.6–99.9)	99.6 (99.3–99.9)
Vitamin E (mg)^c^					
	Adolescents	91.5 (89.2–93.7)	90.3 (89.2–91.4)	89.8 (88.6–91.1)	90.1 (89.3–90.9)
	Adults	95.3 (94.7–95.8)	93.3 (92.0–94.6)	93.4 (93.2–93.7)	93.1 (92.1–94.1)
	Elderly	97.3 (96.5–98.2)	96.0 (95.3–96.7)	96.4 (95.9–96.8)	95.7 (94.4–96.9)
Vitamin C (mg)					
	Adolescents	43.8 (40.6–46.9)	35.2 (33.4–36.9)	27.5 (25.9–29.1)	17.8 (16.1–19.5)
	Adults	64.2 (61.3–67.2)	51.5 (49.3–53.6)	43.1 (41.2–45.1)	29.3 (27.4–31.2)
	Elderly	69.8 (65.6–74.0)	57.3 (54.6–59.9)	48.7 (46.3–51.2)	33.2 (30.1–36.3)

a Retinol activity equivalent (RAE).

b Ergocalciterol (D2) + cholecalciferol (D3).

c Total alpha-tocopherol.

**Table 4:** Prevalence (95%CI) of inadequate micronutrient intakes among women according to per capita household income in the National Dietary Survey 2008–2009.

Nutrients	Age group	Per capita income			
		< 0.5 SM	0.5–1 MW	1–2 MW	> 2 MW
		% (95%CI)	% (95%CI)	% (95%CI)	% (95%CI)
Calcium (mg)					
	Adolescents	99.3 (99.1–99.5)	98.9 (98.7–99.1)	97.9 (97.5–98.2)	94.2 (93.3–95.1)
	Adults	98.2 (97.9–98.6)	96.9 (96.6–97.2)	94.7 (94.1–95.2)	87.8 (86.3–89.4)
	Elderly	99.6 (99.6–99.7)	99.3 (99.2–99.4)	98.6 (98.3–99.0)	96.0 (94.8–97.2)
Magnesium (mg)					
	Adolescents	58.9 (55.8–62.0)	56.4 (52.4–60.4)	56.6 (52.0–61.3)	60.8 (52.9–68.7)
	Adults	69.5 (67.4–71.5)	63.3 (61.3–65.3)	61.5 (60.3–62.7)	64.5 (62.5–66.5)
	Elderly	73.3 (70.8–75.9)	69.2 (66.9–71.5)	67.1 (65.2–69.1)	69.9 (68.3–71.6)
Phosphorus (mg)					
	Adolescents	73.2 (70.6–75.8)	65.1 (63.5–66.8)	59.5 (56.7–62.3)	51.8 (49.8–53.8)
	Adults	19.1 (17.5–20.8)	13.3 (12.0–14.6)	10.4 (9.4–11.3)	7.3 (6.9–7.7)
	Elderly	26.5 (25.0–28.0)	19.7 (17.6–21.8)	15.7 (14.1–17.4)	11.1 (10.1–12)
Copper (mg)					
	Adolescents	8.1 (6.4–9.8)	6.7 (5.9–7.5)	6.8 (5.7–7.9)	7.6 (6.4–8.8)
	Adults	15.7 (13.4–17.9)	12.6 (11.8–13.4)	12.1 (10.1–14.1)	13.2 (12.5–14.0)
	Elderly	17.7 (14.6–20.7)	14.8 (13.1–16.5)	14.4 (12.4–16.4)	15.3 (13.8–16.9)
Zinc (mg)					
	Adolescents	26.6 (22.1–31.2)	19.9 (18.9–20.8)	18.4 (15.7–21.0)	17.7 (16.1–19.3)
	Adults	25.1 (22.3–27.9)	18.0 (16.5–19.4)	16.6 (15.4–17.7)	16.2 (15.4–17.0)
	Elderly	31.9 (26.3–37.5)	24.4 (21.4–27.4)	22.5 (18.9–26.0)	21.6 (19.6–23.7)
Vitamin A (mcg)^a^					
	Adolescents	81.4 (75.9–86.9)	71.6 (68.6–74.7)	65.6 (63.4–67.7)	48.0 (44.7–51.3)
	Adults	88.1 (84.8–91.4)	79.6 (77.3–81.9)	74.3 (71.8–76.8)	57.1 (52.8–61.3)
	Elderly	88.2 (84.4–91.9)	79.8 (77.7–81.8)	73.6 (71.4–75.9)	56.7 (53.9–59.5)
Thiamine (mg)					
	Adolescents	55.5 (50.0–61.1)	41.9 (39.8–44.0)	36.1 (34.0–38.3)	29.4 (25.9–32.9)
	Adults	80.5 (77.4–83.7)	67.6 (65.3–70.0)	61.4 (60.1–62.8)	53.3 (50.5–56.2)
	Elderly	88.5 (85.8–91.2)	78.2 (74.5–81.9)	71.7 (69.4–74.0)	64.4 (59.4–69.3)
Riboflavin (mg)					
	Adolescents	39.6 (32.8–46.4)	25.6 (20.8–30.5)	21.3 (18.1–24.6)	15.5 (11.5–19.6)
	Adults	56.5 (51.9–61.0)	39.6 (36.4–42.8)	34.0 (32.9–35.1)	26.6 (24.2–28.9)
	Elderly	60.5 (53.3–67.6)	44.0 (40.3–47.6)	37.4 (33.9–40.9)	29.7 (25.7–33.7)
Pyridoxine (mg)					
	Adolescents	82.9 (80.4–85.5)	71.5 (67.8–75.2)	66.0 (61.8–70.2)	56.0 (50.1–61.9)
	Adults	96.7 (95.8–97.6)	92.1 (90.4–93.7)	89.5 (87.6–91.4)	84.0 (81.4–86.6)
	Elderly	99.5 (99.4–99.7)	98.3 (97.6–99.0)	97.4 (96.5–98.3)	95.3 (93.4–97.1)
Cobalamin (mcg)					
	Adolescents	9.2 (5.7–12.7)	8.1 (4.3–11.9)	6.9 (4.7–9.2)	4.5 (2.4–6.6)
	Adults	15.2 (12.8–17.7)	12.7 (10.4–15.1)	10.7 (9.2–12.2)	7.5 (6.1–8.8)
	Elderly	18.9 (16.0–21.9)	16.4 (14.2–18.6)	13.6 (12.4–14.9)	9.4 (7.4–11.3)
Vitamin D (mcg)^b^					
	Adolescents	99.6 (99.4–99.7)	99.6 (99.5–99.7)	99.5 (99.3–99.7)	99.1 (98.8–99.3)
	Adults	99.9 (99.8–100)	99.8 (99.8–99.9)	99.8 (99.8–99.9)	99.6 (99.5–99.8)
	Elderly	99.9 (99.8–100)	99.9 (99.8–100)	99.8 (99.8–99.9)	99.7 (99.3–100.1)
Vitamin E (mg)^c^					
	Adolescents	94.5 (93.5–95.6)	93.7 (92.1–95.3)	94.0 (93.0–94.9)	93.9 (92.1–95.7)
	Adults	98.6 (98.5–98.8)	98.1 (97.6–98.6)	98.1 (97.9–98.2)	97.9 (97.6–98.2)
	Elderly	99.1 (98.8–99.4)	98.5 (98.0–99.0)	98.4 (98.3–98.6)	98.3 (98.1–98.6)
Vitamin C (mg)					
	Adolescents	41.2 (36.9–45.5)	30.1 (26.5–33.6)	23.3 (19.1–27.5)	13.5 (10.3–16.7)
	Adults	54.1 (51.3–56.9)	41.1 (39.1–43.0)	32.9 (31.4–34.5)	20.8 (18.6–23.0)
	Elderly	58.6 (53.8–63.4)	46.3 (42.6–49.9)	37.4 (32.9–41.8)	24.3 (21.4–27.2)

a Retinol activity equivalent (RAE).

b Ergocalciterol (D2) + cholecalciferol (D3).

c Total alpha-tocopherol.

**Table 5:** Prevalence (95%CI) of inadequate micronutrient intakes among men according to per capita household income in the National Dietary Survey 2017–2018.

Nutrients	Age group	Per capita income			
		< 0.5 MW	0.5–1 MW	1–2 MW	> 2 MW
Calcium (mg)					
	Adolescents	99.6 (99.5–99.8)	99.0 (98.9–99.2)	97.8 (97.3–98.3)	93.1 (92.1–94.2)
	Adults	98.1 (97.7–98.5)	95.8 (95.2–96.4)	91.7 (90.4–93.0)	81.9 (79.8–83.9)
	Elderly	99.2 (99.0–99.5)	98.2 (97.8–98.7)	96.6 (95.5–97.6)	90.2 (89.1–91.3)
Magnesium (mg)					
	Adolescents	57.4 (54.7–60.1)	51.8 (48.6–54.9)	53.9 (47.7–60.0)	53.1 (45.4–60.9)
	Adults	73.9 (70.1–77.7)	67.4 (65.6–69.3)	68.4 (64.7–72.0)	69.1 (66.8–71.4)
	Elderly	83.7 (81.2–86.3)	78.9 (77.7–80.1)	80.9 (77.9–83.8)	80.5 (78.7–82.2)
Phosphorus (mg)					
	Adolescents	63.1 (59.7–66.4)	51.8 (48.6–55.0)	45.5 (41–50.0)	38.3 (35.0–41.5)
	Adults	4.5 (2.9–6.0)	2.3 (1.9–2.7)	1.8 (1.6–2.0)	1.1 (0.8–1.3)
	Elderly	14 (11.9–16.1)	9.0 (8.3–9.7)	7.2 (6.4–8.1)	4.5 (4–5.1)
Copper (mg)					
	Adolescents	2.2 (1.7–2.6)	1.8 (1.4–2.1)	2.3 (1.6–3.1)	2.2 (1.5–3.0)
	Adults	2.4 (1.6–3.2)	2.0 (1.6–2.3)	2.5 (2.1–3.0)	2.7 (2.3–3.1)
	Elderly	4.1 (2.7–5.5)	3.6 (3.0–4.1)	4.7 (3.9–5.6)	4.7 (4.3–5.1)
Zinc (mg)					
	Adolescents	20.6 (17.0–24.3)	17.6 (14.3–20.9)	15.5 (14.0–16.9)	14.1 (11.7–16.5)
	Adults	28.7 (26.4–31.1)	24.6 (22.8–26.5)	22.3 (20.3–24.3)	21.0 (18.5–23.5)
	Elderly	45.3 (41.9–48.8)	41.1 (38.1–44.1)	39.4 (37.6–41.2)	36.5 (35.3–37.7)
Vitamin A (mcg)^a^					
	Adolescents	92.7 (90.2–95.3)	85.8 (82.3–89.3)	78.6 (73.7–83.4)	64.6 (60.2–69.0)
	Adults	97.7 (97.1–98.3)	94.3 (93.6–95.0)	89.3 (87.8–90.9)	79.7 (78.1–81.3)
	Elderly	98.1 (97.7–98.6)	94.7 (93.7–95.8)	90.2 (88.6–91.8)	80.7 (78.8–82.6)
Thiamine (mg)					
	Adolescents	42.6 (37.7–47.6)	30.1 (27.6–32.6)	26.5 (24.6–28.5)	23.2 (21.1–25.3)
	Adults	67.4 (63.7–71.2)	53.3 (51.9–54.6)	48.8 (45.8–51.8)	45.2 (43.2–47.2)
	Elderly	79.7 (77.3–82.1)	67.6 (65.3–69.9)	63.9 (59.7–68)	59.5 (58.2–60.7)
Riboflavin (mg)					
	Adolescents	46.9 (44.2–49.6)	35.9 (32.0–39.8)	31.3 (27.0–35.7)	25.1 (22.7–27.5)
	Adults	70.4 (68.4–72.4)	58.3 (56.4–60.3)	52.8 (49.8–55.8)	46.1 (43.6–48.6)
	Elderly	75.7 (73.6–77.7)	64.1 (62.6–65.5)	58.8 (56.1–61.5)	51.7 (48.5–54.9)
Pyridoxine (mg)					
	Adolescents	77.5 (75.6–79.4)	68.6 (63.4–73.9)	64.7 (58.8–70.5)	57.0 (53.1–60.8)
	Adults	90.2 (88.1–92.3)	84.2 (82.0–86.4)	81.5 (79.9–83.0)	77.0 (74.0–79.9)
	Elderly	98.5 (98.2–98.7)	96.7 (95.7–97.6)	95.7 (94.8–96.6)	93.3 (92.4–94.1)
Cobalamin (mcg)					
	Adolescents	9.2 (7.3–11.1)	9.0 (7.6–10.5)	6.3 (5.3–7.3)	2.6 (1.7–3.5)
	Adults	10.4 (9.2–11.7)	10.0 (8.5–11.5)	7.0 (5.1–8.8)	3.4 (3.0–3.8)
	Elderly	19.8 (17.8–21.8)	19.8 (17.8–21.8)	15.1 (12.2–18)	7.7 (6.9–8.4)
Vitamin D (mcg)^b^					
	Adolescents	99.9 (99.9–100)	99.9 (99.9–99.9)	99.8 (99.7–99.8)	99.3 (99–99.5)
	Adults	100 (100–100)	100 (100–100)	99.9 (99.9–100)	99.8 (99.7–99.9)
	Elderly	100 (100–100)	100 (100–100)	100 (100–100)	99.9 (99.9– 100)
Vitamin E (mg)^c^					
	Adolescents	91.4 (89.7–93.1)	89.4 (87.7–91)	88.3 (85–91.6)	84.6 (81.0–88.3)
	Adults	94.4 (92.7–96.2)	92.7 (91.7–93.8)	91.4 (89.5–93.3)	89.2 (88.0–90.5)
	Elderly	97.2 (96.0–98.5)	96.2 (95.3–97.2)	95.8 (94.6–97)	94.0 (92.9–95.1)
Vitamin C (mg)					
	Adolescents	47.7 (41.0–54.5)	34.1 (31.4–36.7)	27.6 (24.9–30.2)	17.8 (16.0–19.7)
	Adults	68.0 (63.9–72.2)	53.2 (51.7–54.7)	45.5 (44.3–46.6)	33.3 (32.1–34.5)
	Elderly	72.8 (70.0–75.5)	58.5 (55.0–62.1)	51.3 (48.7–53.8)	38.0 (34.6–41.3)

MW: Minimum Wage.

a Retinol activity equivalent (RAE).

b Ergocalciterol (D2) + cholecalciferol (D3).

c Total alpha-tocopherol.

**Table 6:** Prevalence (95%CI) of inadequate micronutrient intakes among women according to per capita household income in the National Dietary Survey 2017–2018.

Nutrients	Age group	Per capita income			
		< 0.5 MW	0.5–1 MW	1–2 MW	> 2 MW
Calcium (mg)					
	Adolescents	99.8 (99.7–99.9)	99.5 (99.2–99.8)	98.8 (98.0–99.6)	96.1 (95.0–97.1)
	Adults	99.3 (99.2–99.4)	98.4 (97.9–98.9)	96.5 (95.3–97.8)	91.3 (90.6–92.0)
	Elderly	99.9 (99.9–100)	99.7 (99.7–99.8)	99.2 (99.0–99.5)	97.3 (96.9–97.6)
Magnesium (mg)					
	Adolescents	65.9 (61.7–70.2)	63.0 (60.7–65.3)	65.5 (58.9–72.1)	63.9 (58.9–68.8)
	Adults	73.2 (69.9–76.5)	67.2 (65.6–68.7)	67.9 (63.7–72.1)	68.6 (65.4–71.8)
	Elderly	77.3 (71.6–83.0)	72.1 (68.4–75.8)	73.0 (71.3–74.7)	72.8 (72.3–73.4)
Phosphorus (mg)					
	Adolescents	80.3 (77.4–83.2)	70.9 (66.4–75.3)	66.2 (59.4–73.0)	57.7 (51.5–64.0)
	Adults	20.9 (18.3–23.5)	13.9 (12.8–15.0)	11.3 (9.7–12.9)	8.2 (7.2–9.2)
	Elderly	32.5 (26.4–38.7)	23.1 (21.8–24.4)	19.0 (17.9–20.2)	13.8 (12.5–15.1)
Copper (mg)					
	Adolescents	6.2 (5.1–7.3)	5.9 (5–6.7)	7.3 (5.4–9.2)	7.6 (5.1–10.1)
	Adults	10.5 (8.1–13)	9.2 (8.2–10.2)	11.0 (10.2–11.9)	11.6 (10.7–12.4)
	Elderly	11.6 (7.7–15.4)	10.1 (7.9–12.4)	12.1 (10.2–14.0)	12.3 (11.1–13.6)
Zinc (mg)					
	Adolescents	29.2 (26.7–31.7)	25.5 (23.6–27.4)	23.2 (20.3–26.1)	21.4 (17.5–25.3)
	Adults	26.5 (24.0–28.9)	23.3 (21.6–25.0)	21.0 (18.6–23.4)	20.0 (17.8–22.2)
	Elderly	38.7 (34.6–42.7)	34.6 (32.5–36.7)	31.3 (28.4–34.1)	29.3 (27.0–31.7)
Vitamin A (mcg)^a^					
	Adolescents	89.9 (87.3–92.4)	81.5 (78.7–84.4)	72.3 (66.8–77.9)	55.6 (50.9–60.3)
	Adults	93.9 (92.9–95.0)	87.2 (86.4–88.0)	78.9 (77.3–80.5)	64.8 (62.2–67.5)
	Elderly	92.4 (91.4–93.5)	85.0 (83.7–86.2)	75.9 (74.1–77.8)	60.4 (58.4–62.4)
Thiamine (mg)					
	Adolescents	50.3 (45.1–55.4)	39.2 (37.3–41.0)	35.8 (32.4–39.2)	31.2 (29.4–32.9)
	Adults	75.3 (72.3–78.4)	63.2 (61.0–65.4)	58.7 (54.9–62.5)	55.3 (53.5–57.2)
	Elderly	82.0 (78.8–85.3)	70.9 (69.4–72.3)	66.8 (65.0–68.5)	62.5 (60.5–64.4)
Riboflavin (mg)					
	Adolescents	50.6 (45.6–55.5)	39.5 (33.2–45.9)	34.7 (26.7–42.7)	28.1 (23.9–32.3)
	Adults	66.5 (63.6–69.3)	54.6 (51.9–57.4)	48.5 (44.2–52.8)	42.0 (40.4–43.6)
	Elderly	65.8 (63.3–68.4)	53.3 (51.0–55.6)	46.9 (43.5–50.2)	39.7 (37.6–41.9)
Pyridoxine (mg)					
	Adolescents	82.3 (80.3–84.4)	75.9 (72.6–79.1)	72.7 (66.6–78.9)	64.8 (62.0–67.7)
	Adults	96.1 (95.4–96.7)	93.2 (92.0–94.3)	91.4 (89.9–92.8)	88.4 (87.0–89.7)
	Elderly	99.0 (98.7–99.2)	98.0 (97.5–98.6)	97.2 (96.4–98.0)	95.5 (94.8–96.2)
Cobalamin (mcg)					
	Adolescents	13.1 (9.8–16.3)	13.3 (9.5–17.1)	9.9 (5.0–14.9)	5.1 (2.9–7.3)
	Adults	22.4 (20.1–24.7)	22.4 (19.9–24.8)	16.9 (13.6–20.1)	9.6 (8.6–10.5)
	Elderly	30.9 (26.2–35.6)	30.5 (26.1–34.8)	23.5 (18.4–28.5)	13.7 (12.2–15.2)
Vitamin D (mcg)^b^					
	Adolescents	100 (99.9–100)	99.9 (99.9–100)	99.9 (99.8–100)	99.6 (99.5–99.7)
	Adults	100 (100–100)	100 (100–100)	100 (100–100)	99.9 (99.9–99.9)
	Elderly	100 (100–100)	100 (100–100)	100 (100–100)	100 (99.9–100)
Vitamin E (mg)^c^					
	Adolescents	94.9 (93.2–96.6)	94.1 (93.0–95.3)	93.6 (90.3–96.8)	91.7 (89.4–93.9)
	Adults	98.2 (97.6–98.8)	97.5 (96.9–98.1)	96.9 (95.6–98.1)	95.8 (94.7–96.8)
	Elderly	98.9 (98.4–99.5)	98.7 (98.2–99.2)	98.3 (97.5–99.0)	97.5 (96.8–98.2)
Vitamin C (mg)					
	Adolescents	41.4 (33.7–49.1)	28.6 (25.7–31.6)	22.9 (20.1–25.8)	14.1 (12.2–16.1)
	Adults	58.1 (53.4–62.8)	43.2 (41.5–44.9)	35.6 (34.1–37.1)	24.6 (23.2–26.0)
	Elderly	60.2 (54.5–65.9)	45.0 (42.1–47.9)	37.4 (34.3–40.5)	25.8 (23.6–27.9)

MW: Minimum Wage.

a Retinol activity equivalent (RAE).

b Ergocalciterol (D2) + cholecalciferol (D3).

c Total alpha-tocopherol.

**Figure 1:**
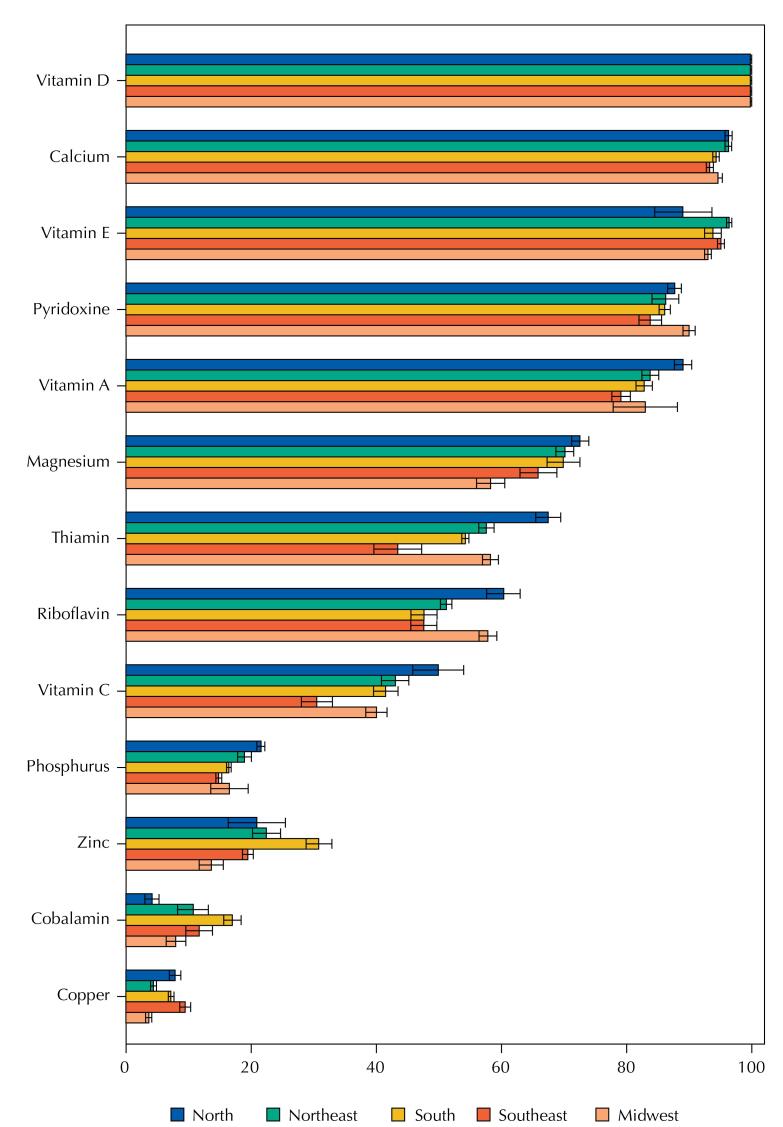
Prevalence (95%CI) of inadequate micronutrient intakes by region in the National Dietary Survey 2017–2018.

**Figure 2:**
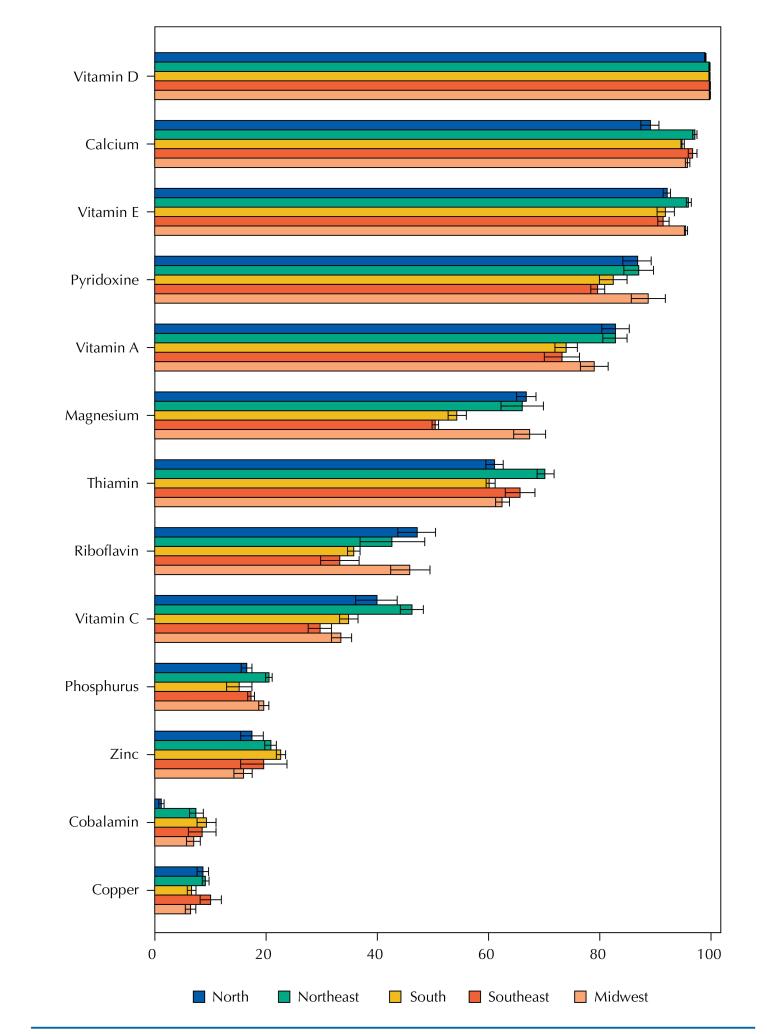
Prevalence (95%CI) of inadequate micronutrient intakes by region in the National Dietary Survey 2008–2009.

When the analyses were stratified by regions of the country, similar results were found for men and women, both in 2008–2009 (Tables 7 and 8) and in 2017–2018 (Tables 9 and 10). We found that thiamine, zinc, vitamin C, magnesium, cobalamin, and copper stood out as the most variable micronutrients between regions (Figures 1 and 2). In 2017–2018, the North and Northeast regions, followed by the Midwest, stood out between the first and second positions with the highest prevalence of inadequacy for most of the analyzed nutrients. Among the statistically significant differences, we chose to highlight the most evident ones (> 15 percentage points).

**Table 7:** Prevalence (95%CI) of inadequate micronutrient intakes among men according to region in the National Dietary Survey 2008–2009.

Nutrients	Age group	Region					
		North	Northeast	South	Southeast	Midwest
		% (95%CI)	% (95%CI)	% (95%CI)	% (95%CI)	% (95%CI)
Calcium (mg)						
	Adolescents	96.8 (96.3–97.3)	98.6 (98.2–99.0)	96.8 (95.8–97.7)	96.5 (95.7–97.3)	98.5 (98.1–98.8)
	Adults	87.5 (86.4–88.5)	93.3 (92.7–93.9)	87.1 (84.8–89.4)	86.7 (85.7–87.8)	92.6 (92.1–93.0)
	Elderly	91.0 (89.7–92.2)	95.6 (94.8–96.5)	90.8 (88.3–93.3)	90.7 (89.1–92.3)	95.0 (94.1–96.0)
Magnesium (mg)						
	Adolescents	47.5 (42.9–52.2)	56.5 (54.3–58.7)	46.6 (45.1–48.1)	52.0 (48.5–55.4)	46.5 (41.8–51.1)
	Adults	64.6 (63.0–66.2)	72.8 (70.9–74.7)	62.0 (60.4–63.6)	68.1 (65.9–70.2)	65.1 (63.6–66.7)
	Elderly	75.5 (70.6–80.4)	82.7 (78.6–86.7)	73.4 (71.4–75.5)	78.7 (74.5–82.9)	76.1 (72.4–79.9)
Phosphorus (mg)						
	Adolescents	44.4 (41.8–47.1)	55.0 (52.2–57.8)	46.7 (44.7–48.7)	52.0 (48.8–55.2)	56.0 (52.5–59.5)
	Adults	1.7 (1.5–2.0)	3.5 (3.1–3.9)	2.4 (1.7–3.1)	3.1 (2.6–3.7)	3.6 (3.1–4.1)
	Elderly	4.6 (3.7–5.6)	8.3 (6.6–9.9)	5.2 (4.5–5.9)	7.0 (5.6–8.3)	8.4 (6.8–10.1)
Copper (mg)						
	Adolescents	3.6 (2.8–4.5)	3.5 (3.0–4.0)	2.5 (2.2–2.8)	3.9 (2.7–5.1)	2.4 (2.0–2.9)
	Adults	4.4 (3.5–5.2)	4.3 (3.8–4.9)	2.9 (2.6–3.3)	4.7 (3.7–5.8)	2.7 (2.4–3.1)
	Elderly	7.6 (5.5–9.7)	7.8 (5.6–10.0)	4.9 (3.7–6.2)	8.2 (5.3–11.1)	4.8 (3.1–6.6)
Zinc (mg)						
	Adolescents	14.1 (10.6–17.5)	16.7 (13.8–19.6)	17.9 (15.3–20.4)	15.7 (11.1–20.2)	12.4 (9.6–15.3)
	Adults	19.1 (17.0–21.2)	22.4 (21.2–23.7)	24.3 (23.2–25.3)	20.9 (16.8–25.0)	17.2 (15.5–18.9)
	Elderly	29.4 (26.6–32.2)	33.9 (30.6–37.2)	35.0 (31.7–38.4)	31.9 (26.6–37.2)	27.5 (24.6–30.5)
Vitamin A (mcg)^a^						
	Adolescents	83.1 (80.1–86.2)	83.7 (80.6–86.7)	75.2 (72.2–78.1)	74.6 (70.2–79.0)	79.1 (75.4–82.7)
	Adults	89.0 (87.3–90.7)	89.0 (87.5–90.5)	82.0 (80.4–83.6)	81.5 (78.6–84.5)	86.1 (84.4–87.8)
	Elderly	89.2 (87.2–91.2)	89.6 (87.4–91.7)	82.1 (80.4–83.9)	82.3 (79.9–84.8)	86.5 (84.4–88.6)
Thiamine (mg)						
	Adolescents	49.9 (47.0–52.7)	48.2 (43.0–53.5)	35.3 (33.1–37.5)	32.0 (28.8–35.1)	48.0 (44.9–51.2)
	Adults	69.5 (67.0–71.9)	67.6 (63.6–71.5)	54.7 (49.9–59.6)	51.3 (49.1–53.4)	68.8 (65.6–72.0)
	Elderly	78.9 (76.4–81.4)	77.7 (74.0–81.4)	65.4 (63.6–67.3)	62.2 (57.9–66.5)	78.3 (75.0–81.6)
Riboflavin (mg)						
	Adolescents	38.9 (34.2–43.6)	34.7 (27.6–41.7)	28.1 (24.6–31.6)	25.7 (21.3–30.2)	36.0 (31.6–40.3)
	Adults	54.9 (51.4–58.4)	49.9 (44.2–55.5)	42.4 (40.6–44.3)	40.2 (36.3–44.2)	53.3 (49.5–57.1)
	Elderly	57.4 (53.8–61.0)	53.0 (47.8–58.3)	44.4 (42.2–46.7)	42.8 (37.6–47.9)	56.8 (53.1–60.4)
Pyridoxine (mg)						
	Adolescents	75.9 (73.4–78.4)	75.2 (71.4–78.9)	66.4 (63.2–69.7)	62.4 (59.8–65.0)	76.6 (73.0–80.1)
	Adults	85.9 (82.8–89.0)	85.2 (81.8–88.5)	79.2 (73.7–84.8)	76.2 (73.5–78.9)	87.2 (83.6–90.7)
	Elderly	97.6 (96.7–98.5)	97.3 (96.2–98.3)	94.9 (92.8–97.0)	94.1 (93.0–95.1)	97.8 (96.8–98.8)
Cobalamin (mcg)						
	Adolescents	1.0 (0.5–1.5)	5.8 (3.9–7.7)	7.5 (5.4–9.6)	6.8 (3.5–10.1)	5.5 (3.6–7.5)
	Adults	0.7 (0.4–1.0)	5.0 (3.9–6.0)	6.4 (5.0–7.8)	5.7 (3.7–7.8)	4.5 (3.6–5.5)
	Elderly	1.2 (0.6–1.8)	7.5 (6.1–8.9)	8.5 (6.3–10.8)	8.0 (5.5–10.6)	6.6 (5.0–8.3)
Vitamin D (mcg)^b^						
	Adolescents	98.5 (97.9–99.0)	99.5 (99.3–99.6)	99.4 (99.2–99.6)	99.5 (99.2–99.8)	99.6 (99.3–99.8)
	Adults	98.9 (98.4–99.4)	99.6 (99.5–99.8)	99.6 (99.4–99.9)	99.7 (99.5–99.9)	99.7 (99.5–99.9)
	Elderly	99.1 (98.6–99.7)	99.8 (99.6–99.9)	99.7 (99.6–99.9)	99.8 (99.6–100)	99.8 (99.7–99.9)
Vitamin E (mg)^c^						
	Adolescents	81.0 (78.6–83.3)	93.8 (92.9–94.6)	89.5 (88.4–90.6)	92.5 (91.1–93.9)	90.9 (89.5–92.3)
	Adults	86.1 (84.0–88.3)	96.1 (95.4–96.7)	92.8 (92.0–93.5)	95.3 (94.2–96.4)	94.2 (93.4–95.0)
	Elderly	90.7 (88.6–92.7)	97.6 (97.3–98.0)	95.3 (94.4–96.2)	97.2 (96.7–97.7)	96.6 (96.1–97.1)
Vitamin C (mg)						
	Adolescents	34.8 (32.2–37.4)	41.2 (38.5–43.8)	30.1 (28.6–31.6)	25.4 (22.5–28.2)	27.9 (25.7–30.1)
	Adults	46.7 (42.9–50.5)	52.9 (50.5–55.3)	40.9 (38.8–43.1)	35.7 (33.6–37.7)	39.7 (37.8–41.6)
	Elderly	48.5 (44.4–52.6)	55.1 (52.1–58.2)	42.3 (39.1–45.4)	37.7 (33.4–42.0)	42.0 (39.1–44.8)

a Retinol activity equivalent (RAE).

b Ergocalciterol (D2) + cholecalciferol (D3).

c Total alpha-tocopherol.

**Table 8:** Prevalence (95%CI) of inadequate micronutrient intakes among women according to region in the National Dietary Survey 2008–2009.

Nutrients	Age group	Region				
		North	Northeast	South	Southeast	Midwest
		% (95%CI)	% (95%CI)	% (95%CI)	% (95%CI)	% (95%CI)
Calcium (mg)						
	Adolescents	97.7 (97.3–98.2)	99.0 (98.7–99.2)	97.4 (97.1–97.7)	97.2 (96.7–97.8)	98.7 (98.4–99.0)
	Adults	92.3 (91.7–92.9)	96.2 (95.8–96.7)	92.6 (91.3–93.8)	92.0 (90.5–93.4)	96.1 (95.6–96.6)
	Elderly	97.1 (96.4–97.8)	98.7 (98.5–98.9)	97.1 (96.3–97.9)	96.7 (95.9–97.5)	98.3 (98.0–98.6)
Magnesium						
(mg)	Adolescents	54.2 (51.6–56.9)	64.1 (61.2–67.0)	54.2 (47.7–60.8)	58.8 (55.0–62.7)	56.6 (53.0–60.2)
	Adults	62.4 (60.6–64.3)	71.0 (69.3–72.6)	59.9 (58.7–61.2)	65.4 (64.0–66.9)	63.1 (61.3–64.9)
	Elderly	67.5 (65.8–69.1)	75.3 (73.6–77.0)	64.9 (63.6–66.3)	69.5 (66.9–72.1)	67.8 (66.0–69.5)
Phosphorus (mg)						
	Adolescents	58.1 (55.9–60.2)	68.3 (66.7–69.8)	61.0 (59.3–62.7)	65.8 (62.8–68.7)	68.5 (66.5–70.4)
	Adults	8.4 (7.8–9.0)	13.6 (12.8–14.4)	10.1 (9.2–11.0)	12.2 (10.9–13.5)	14.0 (13.1–15.0)
	Elderly	11.6 (10.7–12.5)	18.0 (16.4–19.5)	13.1 (12.3–13.8)	15.9 (15.0–16.8)	18.1 (16.9–19.2)
Copper (mg)						
	Adolescents	8.0 (6.9–9.1)	8.4 (7.4–9.4)	6.0 (5.1–6.8)	9.2 (7.0–11.4)	6.1 (4.8–7.4)
	Adults	15.0 (13.5–16.6)	15.0 (13.9–16.1)	11.1 (10.0–12.2)	16.0 (13.6–18.3)	10.9 (9.6–12.2)
	Elderly	17.3 (15.3–19.3)	17.4 (16.2–18.5)	12.7 (11.5–14.0)	18.2 (15.9–20.6)	12.8 (11.3–14.3)
Zinc (mg)						
	Adolescents	18.1 (15.4–20.8)	21.2 (18.8–23.6)	23.1 (20.7–25.4)	19.9 (15.2–24.7)	16.7 (14.2–19.1)
	Adults	15.1 (13.3–16.9)	18.1 (17.3–18.8)	19.9 (19.1–20.7)	16.7 (13.1–20.4)	13.7 (12.2–15.1)
	Elderly	19.6 (16.1–23.1)	23.1 (19.9–26.3)	24.4 (21.7–27.2)	21.1 (17.3–24.9)	17.6 (14.7–20.4)
Vitamin A (mcg)^a^						
	Adolescents	75.4 (71.6–79.2)	75.5 (71.3–79.7)	64.7 (61.1–68.3)	63.8 (59.2–68.5)	70.3 (66.1–74.5)
	Adults	78.6 (75.5–81.7)	78.8 (76.2–81.4)	68.8 (66.3–71.3)	67.8 (64.1–71.4)	74.4 (71.3–77.4)
	Elderly	75.5 (71.5–79.4)	74.8 (72.1–77.6)	64.2 (61.9–66.6)	62.6 (59.3–65.9)	70.7 (67.7–73.7)
Thiamine (mg)						
	Adolescents	51.4 (47.8–55.1)	49.9 (44.8–55.0)	36.6 (34.3–39.0)	32.8 (30.4–35.1)	51.2 (46.1–56.4)
	Adults	73.0 (71.2–74.7)	71.2 (67.8–74.6)	58.9 (56.9–60.9)	54.5 (53.5–55.4)	72.3 (69.5–75.1)
	Elderly	80.4 (77.9–82.9)	77.9 (74.9–81.0)	66.5 (62.5–70.4)	61.9 (60.3–63.6)	79.1 (77.1–81.1)
Riboflavin (mg)						
	Adolescents	34.4 (29.1–39.7)	30.2 (22.3–38.2)	23.7 (20.4–27.0)	21.6 (17.0–26.2)	33.1 (27.2–39.1)
	Adults	45.6 (42.0–49.3)	40.8 (34.9–46.6)	33.5 (32.3–34.6)	30.6 (26.9–34.3)	43.7 (40.2–47.2)
	Elderly	46.5 (40.5–52.5)	41.7 (35.6–47.7)	33.7 (30.9–36.5)	31.1 (26.0–36.2)	44.4 (40.1–48.7)
Pyridoxine (mg)						
	Adolescents	76.9 (72.9–80.9)	76.2 (72.1–80.3)	67.6 (62.1–73.0)	63.2 (61.3–65.1)	78.3 (72.1–84.5)
	Adults	92.4 (90.5–94.3)	92.1 (90.0–94.2)	88.2 (85.4–91.0)	85.5 (83.7–87.3)	93.5 (91.4–95.5)
	Elderly	98.0 (96.9–99.2)	97.6 (96.6–98.6)	96.0 (94.2–97.8)	94.7 (93.3–96.0)	98.0 (97.2–98.7)
Cobalamin (mcg)						
	Adolescents	1.4 (0.5–2.2)	7.4 (4.0–10.8)	9.0 (4.9–13.0)	8.3 (3.7–12.9)	7.2 (4.0–10.3)
	Adults	2.0 (1.3–2.6)	10.1 (8.7–11.5)	12.5 (10.3–14.8)	11.3 (8.3–14.3)	9.6 (8.2–11.0)
	Elderly	2.3 (1.6–3.1)	11.6 (9.8–13.3)	13.5 (11.8–15.3)	12.8 (10.7–14.9)	10.8 (9.0–12.7)
Vitamin D (mcg)^b^						
	Adolescents	98.8 (98.5–99.2)	99.5 (99.4–99.7)	99.5 (99.3–99.7)	99.7 (99.5–99.8)	99.7 (99.6–99.8)
	Adults	99.4 (99.1–99.7)	99.8 (99.7–99.9)	99.8 (99.7–99.9)	99.8 (99.7–99.9)	99.9 (99.8–99.9)
	Elderly	99.5 (99.2–99.8)	99.8 (99.7–100)	99.8 (99.6–100)	99.9 (99.8–100)	99.9 (99.7–100)
Vitamin E (mg)^c^						
	Adolescents	87.0 (84.4–89.6)	96.2 (95.5–96.9)	93.4 (92.3–94.5)	95.5 (94.3–96.6)	94.5 (93.5–95.4)
	Adults	95.0 (93.8–96.1)	99.0 (98.8–99.1)	97.9 (97.6–98.2)	98.6 (98.3–99.0)	98.5 (98.2–98.7)
	Elderly	95.9 (94.7–97.0)	99.1 (98.9–99.4)	98.2 (98.0–98.5)	98.9 (98.7–99.0)	98.5 (98.2–98.8)
Vitamin C (mg)						
	Adolescents	30.8 (26.2–35.4)	36.9 (34.7–39.1)	26.1 (21.5–30.6)	21.5 (18.9–24.1)	25.4 (21.8–29.1)
	Adults	37.1 (33.2–41.0)	43.4 (40.7–46.0)	31.9 (30.7–33.0)	26.5 (24.4–28.7)	30.6 (28.5–32.7)
	Elderly	37.4 (32.2–42.5)	43.9 (41.2–46.6)	31.9 (27.8–36.1)	26.7 (23.7–29.7)	30.9 (27.9–34.0)

a Retinol activity equivalent (RAE).

b Ergocalciterol (D2) + cholecalciferol (D3).

c Total alpha-tocopherol.

**Table 9:** Prevalence (95%CI) of inadequate micronutrient intakes among men according to region in the National Dietary Survey 2017–2018.

Nutrients	Age group	Region				
		North	Northeast	South	Southeast	Midwest
		% (95%CI)	% (95%CI)	% (95%CI)	% (95%CI)	% (95%CI)
Calcium (mg)						
	Adolescents	98.8 (98.5–99.1)	98.6 (98.4–98.9)	97.9 (97.4–98.3)	97.3 (96.9–97.6)	98.0 (97.7–98.4)
	Adults	93.3 (92.1–94.5)	93.2 (92.3–94.0)	89.9 (88.6–91.3)	88.5 (87.4–89.7)	91.1 (90.5–91.7)
	Elderly	96.2 (94.8–97.5)	96.1 (95.0–97.2)	93.8 (93.4–94.3)	92.7 (91.3–94.1)	94.4 (93.4–95.4)
Magnesium (mg)						
	Adolescents	58.4 (56.6–60.2)	55.9 (53.9–57.9)	54.0 (49.7–58.4)	53.4 (46.7–60.2)	43.7 (40.3–47.1)
	Adults	74.4 (73.1–75.6)	71.1 (69.3–72.9)	70.2 (67.7–72.6)	65.8 (63.3–68.3)	58.9 (56.5–61.2)
	Elderly	84.8 (83.1–86.6)	82.3 (80.4–84.3)	81.3 (79.1–83.5)	77.8 (75.0–80.6)	71.8 (70.1–73.6)
Phosphorus (mg)						
	Adolescents	56.1 (54.3–58.0)	52.9 (50.4–55.3)	51.2 (48.0–54.3)	48.3 (46.5–50.2)	50.6 (45.3–55.8)
	Adults	2.7 (2.0–3.4)	2.3 (1.7–2.8)	2.13 (2.0–2.3)	1.78 (1.3–2.3)	1.87 (1.1–2.7)
	Elderly	8.5 (7.0–10.0)	7.4 (6.1–8.7)	7.0 (6.6–7.3)	5.81 (5.0–6.7)	6.57 (4.8–8.3)
Copper (mg)						
	Adolescents	3.0 (2.6–3.4)	1.3 (1.2–1.4)	2.3 (1.8–2.8)	3.5 (2.7–4.4)	1.0 (0.8–1.2)
	Adults	3.3 (2.7–3.9)	1.4 (1.2–1.7)	2.6 (2.2–3.0)	3.7 (3.1–4.3)	1.1 (0.8–1.4)
	Elderly	5.8 (4.6–7.1)	2.7 (2.2–3.2)	4.9 (4.3–5.4)	6.5 (5.8–7.2)	2.3 (1.9–2.8)
Zinc (mg)						
	Adolescents	15.5 (12.3–18.8)	16.1 (12.8–19.3)	22.5 (20.3–24.8)	13.7 (10.6–16.8)	9.2 (6.6–11.8)
	Adults	20.5 (16.1–2)	21.4 (19.1–23.6)	29.3 (27.1–31.5)	18.0 (17.2–18.9)	12.8 (10.8–14.9)
	Elderly	34.7 (28–41.4)	36.3 (33.3–39.2)	45.7 (44.5–46.8)	31.6 (30.1–33.1)	24.3 (21.7–26.9)
Vitamin A (mcg)^a^						
	Adolescents	88.7 (86.2–91.1)	84.0 (81.2–86.8)	82.9 (81.4–84.4)	80.1 (76.9–83.3)	83.0 (77.3–88.7)
	Adults	93.5 (92.6–94.5)	89.9 (88.8–91.1)	89.1 (87.9–90.4)	86.1 (84.9–87.3)	89.1 (84.8–93.5)
	Elderly	92.2 (90.6–93.8)	88.3 (86.1–90.5)	87.6 (86.1–89.2)	84.2 (82.4–86.0)	87.6 (83.4–91.8)
Thiamine (mg)						
	Adolescents	45.2 (41.8–48.5)	34.1 (31.1–37.0)	29.1 (27.1–31.2)	22.4 (18.2–26.6)	33.6 (30.8–36.3)
	Adults	67.2 (65.0–69.3)	55.2 (54.1–56.2)	50.2 (49.4–51.1)	39.3 (35.3–43.3)	55.7 (54.3–57.2)
	Elderly	78.2 (76.1–80.4)	67.4 (65.1–69.8)	63.2 (61.6–64.9)	51.4 (47.4–55.4)	67.6 (65.4–69.9)
Riboflavin (mg)						
	Adolescents	45.8 (43.1–48.4)	37.2 (34.5–39.8)	32.8 (29.7–35.8)	34.6 (31.6–37.7)	42.3 (40.5–44.0)
	Adults	65.6 (62.7–68.5)	56.1 (54.1–58.2)	51.9 (50.8–53.0)	51.7 (49.1–54.3)	62.4 (60.4–64.4)
	Elderly	68.7 (65.8–71.6)	59.1 (57.3–60.9)	55.7 (54.5–57.0)	54.6 (52.1–57.1)	65.7 (63.3–68.0)
Pyridoxine (mg)						
	Adolescents	73.5 (71.0–76.0)	69.5 (64.9–74.0)	66.8 (63.6–70.0)	65.1 (61.0–69.2)	75.7 (73.8–77.7)
	Adults	85.7 (84.1–87.2)	82.8 (79.4–86.1)	81.6 (80.6–82.6)	78.7 (76.0–81.4)	87.5 (85.8–89.2)
	Elderly	96.7 (96.0–97.4)	95.5 (94.5–96.5)	94.8 (94.1–95.5)	93.3 (92.3–94.3)	97.1 (96.6–97.6)
Cobalamin (mcg)						
	Adolescents	2.6 (2.2–3.0)	6.8 (5.3–8.3)	10.8 (9.4–12.1)	7.7 (5.4–10.0)	4.8 (4.0–5.6)
	Adults	2.1 (1.5–2.7)	5.8 (4.4–7.2)	9.9 (9.0–10.9)	6.3 (4.9–7.6)	4.1 (3.3–5.0)
	Elderly	4.3 (2.8–5.8)	10.9 (8.0–13.8)	17.1 (15.8–18.5)	11.5 (9.6–13.4)	8.2 (6.7–9.8)
Vitamin D (mcg)^b^						
	Adolescents	99.8 (99.7–99.9)	99.9 (99.8–99.9)	99.8 (99.7–99.9)	99.8 (99.8–99.9)	99.8 (99.7–99.9)
	Adults	99.9 (99.8–99.9)	99.9 (99.9–100)	99.9 (99.9–100)	99.9 (99.8–99.9)	99.9 (99.8–100)
	Elderly	100 (99.9–100)	100 (100–100)	100 (99.9–100)	99.9 (99.9–100)	99.9 (99.9–100)
Vitamin E (mg)^c^						
	Adolescents	81.8 (76.3–87.2)	92.8 (91.9–93.8)	88.0 (85.3–90.7)	90.6 (89.5–91.6)	87.4 (86.4–88.5)
	Adults	85.3 (79.3–91.3)	94.7 (94.1–95.4)	90.7 (89.2–92.2)	92.7 (91.8–93.6)	90.0 (89.1–91.0)
	Elderly	91.2 (86.3–96.1)	97.3 (96.7–97.8)	94.7 (93.5–96.0)	95.7 (95.0–96.4)	94.2 (93.3–95.1)
Vitamin C (mg)						
	Adolescents	42.7 (38.0–47.3)	36.2 (31.6–40.8)	33.9 (29.2–38.6)	25.3 (20.5–30.1)	32.8 (28.5–37.0)
	Adults	57.2 (53.8–60.5)	50.0 (48.2–51.9)	48.0 (45.7–50.3)	36.1 (33.2–38.9)	46.6 (44.9–48.4)
	Elderly	58.8 (53.4–64.2)	51.4 (48.1–54.8)	50.1 (48.0–52.1)	37.6 (34.0–41.2)	48.6 (45.5–51.7)

a Retinol activity equivalent (RAE).

b Ergocalciterol (D2) + cholecalciferol (D3).

c Total alpha-tocopherol.

**Table 10:** Prevalence (95%CI) of inadequate micronutrient intakes among women according to region in the National Dietary Survey 2017–2018.

Nutrients	Age group	Region				
		North	Northeast	South	Southeast	Midwest
		% (95%CI)	% (95%CI)	% (95%CI)	% (95%CI)	% (95%CI)
Calcium (mg)						
	Adolescents	99.4 (99.0–99.8)	99.3 (98.8–99.7)	98.8 (98.4–99.1)	98.5 (97.8–99.3)	99.0 (98.4–99.6)
	Adults	97.2 (96.5–97.8)	97.2 (96.5–97.8)	95.7 (95.5–95.9)	95.0 (94.2–95.8)	96.3 (95.6–96.9)
	Elderly	99.1 (98.8–99.4)	99.0 (98.9–99.1)	98.4 (98.2–98.5)	97.8 (97.5–98.1)	98.6 (98.4–98.8)
Magnesium (mg)						
	Adolescents	68.9 (66.4–71.4)	65.5 (63.0–68.1)	66.0 (63.3–68.6)	61.2 (57.8–64.6)	54.7 (50.5–58.8)
	Adults	74.0 (72.9–75.1)	70.6 (68.9–72.3)	69.7 (66.7–72.6)	65.6 (62.7–68.6)	58.5 (55.4–61.6)
	Elderly	77.0 (74.2–79.8)	74.8 (72.4–77.3)	74.0 (72.9–75.2)	69.3 (67.0–71.5)	62.8 (58.7–66.9)
Phosphorus (mg)						
	Adolescents	75.0 (71.0–79.0)	72.4 (68.0–76.8)	70.7 (65.8–75.6)	67.8 (64.0–71.6)	69.0 (63.0–74.9)
	Adults	14.7 (13.4–15.9)	13.3 (12.1–14.6)	12.8 (11.7–13.9)	11.2 (10.2–12.3)	11.7 (8.6–14.8)
	Elderly	21.6 (19.2–24.1)	19.5 (16.9–22.1)	18.8 (17.9–19.6)	16.5 (14.6–18.4)	17.6 (13.6–21.7)
Copper (mg)						
	Adolescents	8.6 (7.5–9.7)	4.6 (4.3–4.9)	7.2 (6.0–8.3)	9.8 (8.6–11.1)	3.8 (3.2–4.3)
	Adults	13.3 (12.0–14.6)	7.3 (6.6–8.1)	11.5 (10.8–12.1)	15.1 (13.7–16.4)	6.2 (5.3–7.1)
	Elderly	14.3 (11.8–16.9)	8.3 (6.7–9.9)	12.6 (11.0–14.2)	16.3 (14.2–18.3)	6.9 (5.4–8.4)
Zinc (mg)						
	Adolescents	22.9 (18.7–27.1)	23.6 (20.5–26.8)	31.8 (28.8–34.9)	20.3 (19.1–21.4)	14.5 (12.0–17.0)
	Adults	18.8 (14.3–23.2)	20.3 (18.0–22.6)	27.8 (25.6–29.9)	17.2 (16.3–18.1)	12.0 (9.8–14.2)
	Elderly	27.3 (22.4–32.2)	28.6 (25.8–31.4)	37.5 (34.5–40.5)	24.4 (22.7–26.0)	18.3 (15.8–20.9)
Vitamin A (mcg)^a^						
	Adolescents	85.3 (81.5–89.2)	79.4 (75.0–83.7)	78.4 (76.1–80.6)	73.5 (68.2–78.7)	77.8 (69.6–86.0)
	Adults	86.9 (85.9–87.8)	80.8 (79.6–82.1)	79.9 (77.2–82.5)	75.6 (74.2–77.1)	79.9 (74.4–85.3)
	Elderly	80.0 (77.5–82.5)	73.3 (71.1–75.5)	72.6 (71.3–74.0)	66.8 (64.5–69.1)	72.2 (65.0–79.4)
Thiamine (mg)						
	Adolescents	54.2 (51.0–57.5)	42.8 (40.5–45.1)	38.6 (36.8–40.3)	29.2 (25.0–33.3)	42.9 (41.2–44.5)
	Adults	75.8 (73.7–77.9)	65.2 (63.3–67.1)	60.6 (59.7–61.4)	49.7 (45.8–53.6)	65.7 (64.4–67.0)
	Elderly	79.5 (77.3–81.8)	70.5 (68.7–72.2)	66.2 (65.6–66.9)	54.5 (51.4–57.7)	70.5 (69.2–71.9)
Riboflavin (mg)						
	Adolescents	50.4 (44.5–56.4)	40.8 (35.8–45.8)	36.9 (30.2–43.5)	36.6 (31.1–42.1)	46.6 (41.6–51.6)
	Adults	62.2 (59.7–64.7)	52.6 (51.5–53.6)	48.2 (46.0–50.4)	48.3 (46.4–50.2)	59.2 (58.0–60.4)
	Elderly	57.9 (53.9–61.9)	47.8 (46.0–49.6)	43.9 (41.7–46.2)	43.3 (42.0–44.6)	54.6 (52.5–56.7)
Pyridoxine (mg)						
	Adolescents	79.9 (76.4–83.4)	76.0 (72.4–79.6)	74.7 (71.4–78.1)	70.9 (66.5–75.3)	81.1 (78.2–83.9)
	Adults	93.9 (93.2–94.6)	92.2 (90.7–93.7)	91.4 (90.9–91.9)	89.6 (88.3–90.9)	94.8 (94.3–95.2)
	Elderly	97.9 (97.3–98.4)	97.0 (96.2–97.9)	96.6 (96.2–97.1)	95.4 (94.7–96.2)	98.2 (97.8–98.5)
Cobalamin (mcg)						
	Adolescents	4.2 (1.8–6.6)	10.0 (5.5–14.5)	15.7 (10.8–20.7)	11.0 (6.5–15.5)	7.4 (4.0–10.9)
	Adults	6.1 (4.6–7.6)	14.8 (11.8–17.8)	22.2 (21.0–23.5)	15.9 (13.5–18.3)	11.1 (9.2–13.1)
	Elderly	7.9 (5.7–10.0)	17.8 (13.5–22.1)	26.3 (23.7–28.8)	18.8 (14.9–22.7)	14.0 (10.6–17.3)
Vitamin D (mcg)^b^						
	Adolescents	99.8 (99.7–100)	99.9 (99.9–99.9)	99.9 (99.9–100)	99.8 (99.8–99.9)	99.9 (99.8–99.9)
	Adults	100 (99.9–100)	100 (100–100)	100 (99.9–100)	99.9 (99.9–100)	99.9 (99.9–100)
	Elderly	100 (100–100)	100 (100–100)	100 (100–100)	100 (99.9–100)	100 (99.9–100)
Vitamin E (mg)^c^						
	Adolescents	89.1 (83.8–94.5)	96.0 (95.1–97.0)	93.4 (91.5–95.3)	94.8 (93.5–9.06)	92.3 (91.0–93.6)
	Adults	94.1 (91.0–97.2)	98.3 (98.0–98.7)	96.6 (95.6–97.6)	97.5 (97.0–97.9)	96.3 (95.8–96.9)
	Elderly	95.9 (92.7–99.2)	98.9 (98.6–99.2)	97.9 (97.2–98.6)	98.3 (97.9–98.6)	97.5 (97.0–97.9)
Vitamin C (mg)						
	Adolescents	37.5 (32.3–42.7)	30.9 (26.1–35.8)	29.2 (25.5–32.9)	20.0 (17.1–22.9)	28.0 (24.7–31.4)
	Adults	48.0 (44.0–51.9)	40.8 (38.5–43.1)	38.9 (37.0–40.8)	27.9 (25.4–30.5)	37.6 (35.6–39.6)
	Elderly	45.2 (41.0–49.4)	38.4 (36.0–40.9)	36.6 (33.4–39.8)	25.5 (21.7–29.2)	35.2 (32.7–37.7)

a Retinol activity equivalent (RAE).

b Ergocalciterol (D2) + cholecalciferol (D3).

c Total alpha-tocopherol.

Discrepancies were found in the prevalence of inadequacy of thiamine between the North and Southeast and of zinc between the South and Midwest, whose estimates, in all age groups, were 1.5 to 2 times higher in the first region compared to the last, respectively. The Southeast region stood out with lower prevalence of inadequacy of thiamine in relation to the Northeast and Midwest regions among adults and the elderly, while the South, in the second position of least inadequacy, still differed in relation to the North region among adolescents and adults. In addition, the North region emerged with the highest differences in the prevalence of inadequate vitamin C in contrast to the Southeast region among men and women of all age groups, and magnesium in contrast to the Midwest region for adults, but with a lower prevalence of inadequacy of cobalamin in relation to the South region among adult and elderly women (Tables 9 and 10).

## DISCUSSION

Despite the gap of a decade between the two National Dietary Surveys, energy consumption did not vary significantly over the period, and the nutrients that caused concern in the first survey remain those with the highest prevalence of inadequacy in the second survey, with emphasis on calcium, vitamins A, D and E, magnesium, and pyridoxine. The high prevalence of excessive sodium intake in both surveys is also noteworthy, especially among adult men.

The poorest individuals were more vulnerable to inadequate consumption of most of the investigated nutrients, with special attention to vitamins A and C, thiamine, and riboflavin, which had a great absolute and relative difference in comparison with the higher-income stratum. Demographic disparities were observed according to the regions of the country and, in general, the prevalence of inadequate consumption was higher for the North region, followed by the Northeast or Midwest regions.

The prevalence of inadequacy presented here is based on ideal values for the maintenance of biochemical markers in healthy individuals. Therefore, they do not necessarily indicate the appearance of clinical health outcomes. These outcomes result from metabolic mechanisms that involve the interaction between bioavailability and nutrient requirements, as well as individual characteristics related, for example, to genetic factors and nutritional status throughout life^18^.

Few studies with a representative sample of the Brazilian population analyzed the prevalence of inadequate nutrient intake. Among them, the Study of Cardiovascular Risks in Adolescents (Erica)^19^ evaluated Brazilian adolescents and pointed out as nutrients with the highest prevalence of inadequacy the same ones observed in this analysis; for example, calcium (99% prevalence of inadequacy), vitamins A (prevalence inadequacy ranging between 60% and 74%) and E (100% inadequacy prevalence), and sodium (overconsumption prevalence ranging between 79% and 91%).

A 50% inadequacy in phosphorus intake among the adolescents investigated in this analysis was also observed in the Erica study, which found even higher prevalence of inadequacy, between 59% and 65%. The greater inadequacy of phosphorus in this age group is due to the higher recommended intake, approximately twice that recommended for adults^20^. The importance of phosphorus in adolescence is also highlighted, along with calcium and vitamin D, for the proper maintenance of bone metabolism and prevention of diseases such as scoliosis and osteoporosis in older ages^21^.

Studies in Europe, North America, Latin America, Africa, and Asia also point to inadequate nutrient intake in different populations. Each region has specific characteristics regarding the nutrients whose intake by its population is most critical. However, in general, there were high prevalence of inadequate intake of calcium, vitamins A, D, E, zinc, folate, and iron^22,23^. The evolution of nutrient inadequacy was also assessed with data from adults from the North American National Health and Nutrition Examination Survey, from 2003 to 2016. A reduction in inadequacy was observed for most nutrients. Nonetheless, calcium, magnesium, vitamins A, C, D, and E, and folate remained inadequate above 50% in 2015–2016^24^. One should note, however, that although different studies indicate high prevalence of inadequacy for the same nutrients, such results need to be viewed with caution, as research differs in dietary assessment methods, reference values, and analyses used to estimate usual consumption.

Some important aspects about the high prevalence of inadequate intake of calcium and vitamin D need to be pointed out. The reference values for these two nutrients were revised in the last decade, based on the best evidence available at the time^20^. However, to establish these values little or no sun exposure^20^ was assumed, which may not be applicable in countries with a predominantly tropical climate, such as Brazil. Part of the need for vitamin D can be met by synthesis from sun exposure. Accordingly, in a study carried out in the city of São Paulo, almost 100% of the population had inadequate intake of vitamin D, but approximately half of the people had an adequate serum level^25^.

Calcium requirements are also a source of intense debate, involving both the adequacy of balance studies and the non-confirmation of beneficial effects for bone health in meta-analyses of longitudinal studies^26^. However, despite criticisms of reference values, the frequency of consumption of foods that are sources of calcium and vitamin D, such as dairy products and fish, has been very low since the 2008–2009^10^.

Excessive and inadequate consumption of sodium remained between the two surveys, a result in line with the 2013 National Health Survey, which estimated the consumption of salt in the Brazilian population by the urinary excretion of sodium. The verified consumption, higher among men, was twice the maximum amount of intake recommended by the World Health Organization^27^.

The high prevalence of excessive sodium intake has been a concern for decades in Brazil^28^ and worldwide^29^. The excessive consumption, associated with a diet low in potassium, increases blood pressure levels and represents a risk for the development of arterial hypertension, which in turn has been listed as one of the most important risk factors for morbidity and mortality from cardiovascular diseases^30^. Another outstanding result was the increase, among the elderly, of riboflavin inadequacy, a vitamin that can exert neuroprotective effects for some neurological disorders (for example, Parkinson’s disease, migraine, and multiple sclerosis), with milk and dairy products, viscera, and beef as its main sources^31^.

Individuals with lower per capita household income were those with the highest prevalence of inadequacy for most of the investigated nutrients. Similarly, in general, poorer regions of the country had higher prevalence of inadequacy. Studies with the North American population also showed that, among adults of low socioeconomic status, the proportion of individuals with inadequate nutrient intake increased^24^, while at higher income levels the risk of inadequacy decreased^32^.

The socioeconomic and demographic disparities highlighted here have already been observed in other analyses of the 2008–2009 survey^33^ and in other samples of Brazilian population base^34^. These disparities were also observed in the assessment of food consumption using the same database, with a reduction in the frequency of fruit consumption between 2008–2009 and 2017–2018, more pronounced in the lowest quartile of income. In contrast, the 2017–2018 results showed that typically Brazilian foods, such as rice, beans, manioc flour, corn and corn-based foods were consumed more frequently among low-income individuals^10^.

Regional differences were observed along with income differences. Foods such as cassava flour, corn, and corn-based foods were more frequently consumed in poorer regions such as the North and Northeast, respectively. On the other hand, the South and Southeast regions had the highest average per capita consumption of fruits and vegetables^10^. Also noteworthy is that results of the POF 2017–2018 also showed how the regular and permanent access of households to quality food in sufficient quantity is unequal in Brazil. The North and Northeast regions had the lowest proportions of private households in food security situation. Less than half of the residents of these regions had full and regular access to food^5^. Therefore, any strategy that aims to improve nutrient intake in Brazil needs to emphasize access to adequate food for the most disadvantaged individuals living in the poorest regions.

This study has some limitations. First, nutrient intake did not consider the information on the use of nutritional supplements collected in the last survey. Individuals were only asked about their use in the last 30 days, but without information on the amount and frequency consumed. Therefore, was not possible to estimate the amount of nutrients provided by supplements. The use of multivitamins, B-complex vitamins, and vitamin C was reported by 11.1% of the population, ranging up to 19.5% in elderly women, and the use of calcium supplementation was reported by 4.6%, ranging up to 21.3%, also in elderly women.

The second limitation was the change in the collection method of food consumption, which makes it difficult to compare the two surveys. The change occurred because validation studies systematically showed that collection using computer-assisted R24h decreases the underreporting of energy, sodium, potassium, and protein^35^. Thus, it is believed the change has allowed for more accurate estimates. However, the underreporting of energy intake, common in the collection by R24h, probably led to an overestimated prevalence of inadequacy. However, as caloric intake was similar between both surveys (1,753 kcal in 2008–2009 and 1,748 kcal in 2017–2018), it is likely that the effect of this possible underreporting was similar.

The high and persistent prevalence of inadequacy verified in this study point to a nutritionally deficient diet, in a context of global syndemic of obesity, malnutrition, and climate change^36^. Given this scenario, FAO recognizes the challenge of combating poor nutrition in all its forms, including malnutrition, specific nutritional deficiencies, overweight, obesity, and food-related CNCD^37^.

In Brazil, access to adequate food as a right is provided for by law^38^ and incorporated into the Federal Constitution^39^. From this perspective, physical and economic access to food must ensure not only sufficient food, but also adequate nutrition. However, as shown here, nutrient intake has been inadequate among Brazilians.

## CONCLUSION

High prevalence of inadequate nutrient intakes and excessive sodium intake were found in both surveys. The prevalence of inadequacy changes according to sociodemographic variables, increasing among individuals in the lowest income strata and in the poorest regions of the country.
