# Supplementary material for: Evolution of energy and nutrient intake in Brazil between 2008–2009 and 2017–2018
Source: Rev Saude Publica. 2021 Nov 22;55 Supl 1:5s. doi: 10.11606/s1518-8787.2021055003343 (PMC9586436; doi:10.11606/s1518-8787.2021055003343)
Supplement: Translation

# Translation: Evolução da ingestão de energia e nutrientes no Brasil entre 2008–2009 e 2017–2018

**Authors:** Eliseu Verly, Dirce Maria Marchioni, Marina Campos Araujo, Eduardo De Carli, Dayan Carvalho Ramos Salles de Oliveira, Edna Massae Yokoo, Rosely Sichieri, Rosangela Alves Pereira

**Affiliations:** I Rio de Janeiro RJ Brasil Universidade do Estado do Rio de Janeiro. Instituto de Medicina Social. Departamento de Epidemiologia. Rio de Janeiro, RJ, Brasil; II Rio de Janeiro RJ Brasil Fundação Oswaldo Cruz. Escola Nacional de Saúde Pública Sérgio Arouca. Departamento de Epidemiologia e Métodos Quantitativos em Saúde. Rio de Janeiro, RJ, Brasil; III São Paulo SP Brasil Universidade de São Paulo. Faculdade de Saúde Pública. Departamento de Nutrição. São Paulo, SP, Brasil; IV Niterói RJ Brasil Universidade Federal Fluminense. Instituto de Saúde Coletiva. Departamento de Epidemiologia e Bioestatística. Niterói, RJ, Brasil; V Rio de Janeiro RJ Brasil Universidade Federal do Rio de Janeiro. Instituto de Nutrição Josué de Castro. Departamento de Nutrição Social e Aplicada. Rio de Janeiro, RJ, Brasil

**Keywords:** Consumo de Alimentos, Ingestão de Energia, Deficiências Nutricionais, epidemiologia, Alimentos, Dieta e Nutrição, Inquéritos sobre Dietas

## Abstract

**OBJETIVO:**

Avaliar a evolução da ingestão de energia e nutrientes e a prevalência de inadequação da ingestão de micronutrientes segundo características sociodemográficas e regiões brasileiras.

**MÉTODOS:**

Foi analisado o consumo alimentar de 32.749 indivíduos do Inquérito Nacional de Alimentação da Pesquisa de Orçamentos Familiares de 2008–2009, por dois registros alimentares, e de 44.744 indivíduos a partir de dois recordatórios de 24 horas em 2017–2018. Estimaram-se a ingestão usual e o percentual de indivíduos com consumo abaixo da necessidade média para cálcio, magnésio, fósforo, cobre e zinco, vitaminas A, C, D, E, tiamina, riboflavina, piridoxina e cobalamina. A ingestão de sódio foi comparada ao valor de referência para reduzir risco de doenças crônicas. As análises foram estratificadas por sexo, faixa etária, região e renda.

**RESULTADOS:**

A ingestão energética diária média foi de 1.753 kcal em 2008–2009 e 1.748 kcal em 2017–2018. As prevalências de inadequação mais elevadas (> 50%) nos dois períodos foram de cálcio, magnésio, vitaminas A, D e E, piridoxina e, somente entre adolescentes, fósforo. Houve aumento na prevalência de inadequação de vitamina A, riboflavina, cobalamina, magnésio e zinco entre as mulheres, e de riboflavina entre os homens. A prevalência de inadequação diminuiu para a tiamina. A ingestão de sódio foi excessiva em aproximadamente 50% da população nos dois períodos. As variações mais altas (cerca de 50%) nas prevalências de inadequação entre os extremos de renda (< 0,5 salário-mínimo e > 2 salários-mínimos per capita) foram observadas para vitamina B12 e C nos dois períodos. As regiões Norte e Nordeste apresentaram maiores prevalências de inadequação.

**CONCLUSÃO:**

Ambos os inquéritos verificaram prevalências elevadas de inadequação de ingestão de nutrientes e consumo excessivo de sódio. A inadequação varia de acordo com os estratos de renda, aumentando nas regiões mais pobres do país.

## INTRODUÇÃO

O impacto de dietas de baixa qualidade na morbidade e mortalidade por doenças crônicas não transmissíveis (DCNT) em todo o mundo foi maior que o de qualquer outro fator de risco, incluindo tabagismo, segundo o Global Burden of Disease^1^. A má nutrição em países de baixa e média renda é caracterizada com frequência pelo excesso de ingestão de energia e deficiência de micronutrientes, tornando desafiador o enfrentamento da dupla carga de doenças geradas pela coexistência da obesidade e da desnutrição, além de outras doenças não transmissíveis relacionadas à dieta^2^.

A segurança alimentar e a nutrição estão intimamente ligadas. A insegurança alimentar se relaciona com a má qualidade da dieta, que por sua vez está ligada a manifestações de má nutrição que aumentam o risco de desnutrição, sobrepeso e obesidade^2^. Em 2015, os países das Nações Unidas, que incluem o Brasil, se comprometeram com os 17 Objetivos de Desenvolvimento Sustentável (ODS) da Agenda 2030. No ODS 2, a meta 2.2 é acabar com todas as formas de má nutrição^3^.

No Brasil, em 2019, 26% da população maior de 18 anos estava obesa e cerca de 60% com sobrepeso^4^. Entre 2002–2003 e 2019, a proporção de obesos na população com 20 anos ou mais de idade mais do que dobrou, indo de 12,2% para 26,8%^4^. Por outro lado, dados da Pesquisa de Orçamentos Familiares mostraram que, em 2017–2018, dos 68,9 milhões de domicílios no Brasil, 36,7% (o equivalente a 25,3 milhões) estavam com algum grau de insegurança alimentar^5^.

Os efeitos benéficos de um padrão saudável de alimentação derivam de um efeito cumulativo e sinergético de nutrientes de várias fontes alimentares^6^. Vitaminas e minerais são essenciais para os seres humanos e têm um papel importante em uma miríade de ciclos metabólicos que auxiliam ações celulares. Esses efeitos incluem a saúde cardiovascular e óssea, bem como a estrutura e a função do sistema nervoso^7,8^.

A análise da situação nutricional e do consumo alimentar de uma população pode seguir várias abordagens complementares, que abrangem a ingestão de nutrientes, grupos de alimentos e padrões da dieta. A avaliação da ingestão de nutrientes permite conhecer os grupos da população em que há risco de inadequação, tanto para planejar ações quanto para analisar políticas públicas já implementadas, como as de fortificação de alimentos. Recomendações para a ingestão de nutrientes almejam assegurar que a maior parte da população receba quantidades que supram as necessidades fisiológicas^9^.

No Inquérito Nacional de Alimentação (INA) realizado em 2008–2009^10^, o primeiro a coletar dados de consumo alimentar individual no Brasil, prevalências elevadas de inadequação foram observadas para vitamina E, vitamina D, cálcio, vitamina A, magnésio e vitamina C em adolescentes, adultos e idosos de ambos os sexos. Tendo em vista esse resultado, o presente estudo tem como objetivo avaliar a evolução da ingestão de energia e nutrientes e a prevalência de inadequação da ingestão de micronutrientes entre adolescentes, adultos e idosos brasileiros segundo características sociodemográficas e regiões brasileiras.

## MÉTODOS

Foram analisados dados de dois Inquéritos Nacionais de Alimentação (INA), conduzidos em 2008–2009 e 2017–2018 como módulos da Pesquisa de Orçamentos Familiares (POF). Nos dois inquéritos, a amostragem foi definida por conglomerados, em dois estágios: no primeiro, foram sorteados os setores censitários; no segundo, os domicílios dentro de cada setor. Detalhes sobre o plano amostral são encontrados em outras publicações^10,11^.

As amostras dos INA corresponderam a 24,2% e 34,7% dos domicílios amostrados para a POF, totalizando 13.569 domicílios e 34.003 indivíduos em 2008–2009, e 20.112 domicílios e 46.164 indivíduos em 2017–2018. Todos os indivíduos com 10 anos ou mais de idade nos domicílios selecionados foram convidados a participar do módulo de consumo individual. Gestantes e lactantes não foram consideradas nessas análises (n = 1.254 em 2008–2009; n = 1.420 em 2017–2018), totalizando 32.749 e 44.744 indivíduos no primeiro e no segundo inquérito, respectivamente.

### Coleta e Tratamento dos Dados

No inquérito de 2008–2009, os dados de consumo alimentar foram obtidos por meio de registro alimentar preenchido pelo morador em dois dias não consecutivos (97% responderam ao segundo registro). Os indivíduos foram orientados a anotar com detalhes todos os alimentos e bebidas consumidos, incluindo o modo de preparo, ingredientes e quantidades, preferencialmente em medidas caseiras. Ainda nos domicílios, os agentes de pesquisa passaram as informações de consumo alimentar para programa específico desenvolvido pelo Instituto Brasileiro de Geografia e Estatística^11^.

No inquérito de 2017–2018, a coleta foi feita por meio de recordatórios de 24 horas (R24h) aplicados em dois dias não consecutivos, na mesma semana em que os dados de despesa familiar foram obtidos (84% responderam ao segundo R24h). Os indivíduos foram orientados a relatar todos os alimentos e bebidas consumidos no dia anterior às duas entrevistas, incluindo descrição detalhada dos alimentos (ingredientes, adições, modo de preparo) e quantidades. As entrevistas foram estruturadas segundo o método automatizado de múltiplas passagens^12^, utilizando para a coleta de dados um software especificamente elaborado para o inquérito, com 12 opções de itens que poderiam ser adicionados aos alimentos (azeite, manteiga, margarina, maionese, queijo, creme de leite, açúcar, mel, melado, ketchup, mostarda e molho shoyu).

Para analisar a quantidade e a composição nutricional das adições, considerou-se o tipo de adições reportadas. Adições à base de gordura (azeite, manteiga/margarina, maionese, queijo e creme de leite), quando relatadas, poderiam acrescentar no máximo 20% ao consumo, em gramas, do alimento ao qual foram adicionadas. As adições de açúcar, mel, melado, ketchup, mostarda e molho shoyu representaram no máximo 10% do consumo do item. Ou seja, se a um sanduíche foi adicionado ketchup e mostarda, cada adição representa 5% da gramatura do sanduíche. Detalhes sobre coleta dos dados, treinamento, procedimentos de crítica e imputação podem ser obtidos na publicação oficial^10^.

### Composição Nutricional dos Alimentos

Para conversão dos alimentos relatados em quantidades de energia e nutrientes, em ambos os inquéritos utilizou-se a versão 7.0 da Tabela Brasileira de Composição de Alimentos (TBCA), elaborada pela Rede Brasileira de Dados de Composição de Alimentos, Universidade de São Paulo e Centro de Pesquisa em Alimentos^13^. É importante mencionar, porém, que a tabela de composição nutricional mudou entre os inquéritos. A justificativa dessa mudança se deve ao fato de a TBCA descrever dados nutricionais de alimentos brasileiros com a confiabilidade assegurada pela Rede Internacional de Sistema de Dados de Alimentos, da Organização das Nações Unidas para a Alimentação e a Agricultura (FAO), que determina diretrizes e critérios a serem usados na geração, compilação e utilização de dados de composição de alimentos^14^. Assim, para reduzir a possibilidade de divergências nas avaliações de tendência e permitir comparabilidade, a TBCA foi utilizada nas estimativas de energia e nutrientes dos dois inquéritos.

### Análise Estatística

A distribuição da ingestão usual de cada nutriente foi estimada pelo método do National Cancer Institute (NCI) que aplica um modelo de regressão de efeito misto após a transformação Box-Cox dos dados de consumo^15^. São estimados parâmetros (média populacional, variâncias inter e intrapessoal, efeitos das variáveis grupo etário e sexo, renda e região e o lambda da transformação Box-Cox) que são posteriormente utilizados para construir as distribuições de consumo usual por meio de simulação de Monte Carlo.

As distribuições da ingestão usual foram estimadas para cada grupo etário e sexo considerando as recomendações de ingestão de nutrientes definidas. As prevalências de inadequação foram estimadas utilizando o método da EAR (*estimated average requirement*) como ponto de corte, que representa o percentual de indivíduos com ingestão abaixo do valor médio estimado de necessidade para cada grupo etário e sexo^16^.

A ingestão de sódio foi comparada ao valor de referência para redução de risco de doenças crônicas (*chronic disease reduction risk intakes*), como proposto na última revisão dos valores de referência para sódio do Instituto de Medicina dos Estados Unidos^17^. A partir dessa referência calcula-se o percentual da população que deveria diminuir o consumo de sódio para reduzir o risco de doenças crônicas. Considerando a incompatibilidade entre os dados de niacina pré-formada, disponíveis na TBCA, com os valores de EAR expressos em equivalentes de niacina, prevalências de inadequação não foram calculadas para esse nutriente.

A prevalência de inadequação foi estimada para cada grupo etário e sexo para os quais há valores estabelecidos de necessidade média de ingestão (9 a 13 anos de idade, 14 a 18, 19 a 30, 31 a 50, 51 a 70, e 70 ou mais). Em seguida, foram calculadas as médias das prevalências ponderadas pelo tamanho amostral expandido de cada grupo etário, estratificadas em faixas etárias correspondentes aos ciclos de vida: adolescentes (10–18 anos), adultos (19–59 anos) e idosos (60 anos ou mais). O mesmo procedimento foi feito para totalizar prevalências por estratos de renda e região do país. Para os estratos de renda, utilizou-se o rendimento per capita das famílias: menor que 0,5, entre 0,5 e 1, entre 1 e 2, e maior que 2 salários-mínimos per capita. Os valores de salários-mínimos nas datas de referência das pesquisas foram: R$ 415,00 (15 de janeiro de 2009) e R$ 954,00 (15 de janeiro de 2018).

Os intervalos com 95% de confiança (IC95%) foram calculados para as médias e as prevalências de inadequação com base nos valores de erro-padrão corrigidos pelo desenho amostral, estimados pela técnica de replicação *balanced repeated replication* (BRR), usada no método do NCI. Utilizou-se o IC95% para comparar as prevalências de inadequação entre sexos, faixas etárias, renda, regiões do país e os dois períodos avaliados. As análises foram executadas no software SAS versão 9.4.

## RESULTADOS

As médias (IC 95%) de ingestão energética diária se mostraram semelhantes nos dois inquéritos: 1.753 kcal (1.734–1.772) em 2008–2009 e 1.748 kcal (1.732–1.763) em 2017-2018. Entre homens, a ingestão média calórica em 2008–2009, para adolescentes, adultos e idosos, foi de 1.996 kcal (1.945–2.047), 1.969 kcal (1.940–1.998) e 1.680 kcal (1.633–1.726), respectivamente, e 1.969 kcal (1.927–2.010), 2.018 kcal (1.993–2.044) e 1.708 kcal (1.669–1.747) em 2017–2018. Entre as mulheres, os valores foram de 1.753 kcal (1.716–1.798), 1.549 kcal (1.539–1.582) e 1.410 kcal (1.370–1.449) em 2008–2009, e 1.674 kcal (1.633–1.715), 1.549 kcal (1.530–1.568) e 1.409 kcal (1.381–1.437) em 2017–2018 para as mesmas faixas de idade, respectivamente.

Observa-se menor ingestão de energia entre mulheres e com o aumento da idade nos dois inquéritos, com exceção de homens adultos, cujas médias não diferiram da de adolescentes. Entretanto, em ambos os inquéritos, homens adultos, quando comparados às demais faixas etárias e às mulheres, tiveram as mais elevadas médias de ingestão para a maioria dos micronutrientes. Observou-se redução discreta (< 23%) entre os inquéritos nas médias de ingestão para a maioria dos micronutrientes, enquanto aumentos nas médias de ingestão (4%–10%) ocorreram somente entre adultos do sexo masculino para tiamina, e entre adultos de ambos os sexos para vitamina E (Tabela 1).

**Tabela 1:** Médias (IC95%) de ingestão de vitaminas e minerais entre adolescentes, adultos e idosos nos Inquéritos Nacionais de Alimentação 2008–2009 e 2017–2018.

Nutrientes		Adolescentes		Adultos		Idosos	
		2008–2009	2017–2018	2008–2009	2017–2018	2008–2009	2017–2018
Homens							
	Cálcio (mg)	470.1 (455.5–484,7)	457,1 (436,5–477,6)	483,7 (469,6–497,7)	472,7 (459,0–486,4)	469,3 (453,3–485,3)	446,3 (434,8–457,7)
	Magnésio (mg)	285,8 (280,3–291,2)	273,3 (266,6–280,0)	310,0 (307,2–312,8)	304,7 (301,5–307,9)	285,3 (277,6–293,0)	275,7 (270,6–280,8)
	Fósforo (mg)	1.094,6 (1.074,7–1.114,4)	1.081,9 (1.067,0–1.096,8)	1.167,2 (1.147,3–1.187,0)	1.153,4 (1.144,9–1.161,8)	1.036,6 (1.022,7–1.050,5)	977,1 (959,8–994,4)
	Sódio (mg)	2.784,4 (2.706,2–2.862,5)	2.805,5 (2.745,6–2.865,5)	2.910,9 (2.866,9–2.954,8)	2.961,0 (2.922,1– 2.999,8)	2.462,1 (2.369,4–2.554,7)	2.472,8 (2.396,8–2.548,8)
	Potássio (mg)	2.387,5 (2.325,5–2.449,4)	2.218,2 (2.166,8–2.269,6)	2.607,3 (2.571,4–2.643,1)	2.492,4 (2.459,6–2.525,3)	2.484,7 (2.412,2–2.557,2)	2.275,7 (2.229,3–2.322,1)
	Cobre (mg)	1,4 (1,4–1,5)	1,4 (1,4–1,5)	1,6 (1,5–1,6)	1,6 (1,5–1,6)	1,4 (1,3–1,5)	1,4 (1,4–1,4)
	Zinco (mg)	11,8 (11,3–12,2)	11,5 (11,1–11,9)	12,9 (12,7–13,1)	12,7 (12,5–12,8)	11,5 (11,2–11,9)	10,9 (10,8–11,0)
	Vitamina A (mcg)^a^	376,3 (343,2–409,3)	324,6 (307,6–341,7)	378,5 (359,3–397,8)	320,1 (306,6–333,6)	376,5 (357–396,0)	342,4 (324,7–360,0)
	Tiamina (mg)	1,0 (1,0–1,1)	1,1 (1,1–1,2)	1,0 (0,9–1,0)	1,1 (1,1–1,1)	0,9 (0,8–0,9)	0,9 (0,9–1,0)
	Riboflavina (mg)	1,3 (1,3–1,4)	1,2 (1,2–1,3)	1,2 (1,2–1,3)	1,1 (1,1–1,1)	1,2 (1,2–1,2)	1,1 (1,1–1,1)
	Piridoxina (mg)	0,8 (0,8–0,8)	0,8 (0,8–0,9)	0,8 (0,7–0,8)	0,8 (0,8–0,8)	0,6 (0,6–0,7)	0,7 (0,7–0,7)
	Cobalamina (mcg)	5,0 (4,7–5,3)	4,1 (3,9–4,4)	5,6 (5,5–5,8)	4,6 (4,5–4,7)	5,2 (4,9–5,4)	3,9 (3,8–4,0)
	Vitamina D (mcg)^b^	2,4 (2,3–2,5)	2,0 (1,9–2,0)	2,2 (2,0–2,3)	1,7 (1,6–1,7)	2,0 (1,9–2,1)	1,5 (1,5–1,5)
	Vitamina E (mg)^c^	6,6 (6,4–6,8)	6,7 (6,6–6,9)	7,0 (6,9–7,0)	7,3 (7,2–7,5)	6,2 (6,1–6,4)	6,4 (6,3–6,6)
	Vitamina C (mg)	124,0 (119,2–128,8)	117,1 (108,2–126,0)	131,4 (127,9–134,9)	119,8 (117,2–122,4)	126,0 (118,9–133,0)	116,5 (107,0–126,0)
	Niacina (mg)^d^	15,6 (14,9–16,2)	17,3 (16,3–18,4)	18,6 (18,0–19,2)	19,7 (19,1–20,3)	15,5 (14,6–16,3)	15,8 (15,0–16,6)
Mulheres							
	Cálcio (mg)	447,2 (435,4–458,9)	406,6 (379,2–434,1)	429,4 (419,7–439,2)	398,7 (394,1–403,2)	451,3 (425,5–477,1)	429,3 (418,5–440,0)
	Magnésio (mg)	244,3 (238,9–249,7)	229,7 (224,2–235,2)	240,8 (238,9–242,7)	232,8 (229,1–236,6)	232,4 (229,9–234,9)	225,5 (221,9–229,1)
	Fósforo (mg)	977,1 (962,4–991,7)	924,6 (889,9–959,3)	928,9 (923,1–934,7)	878,9 (861,3–896,6)	877,3 (866,2–888,3)	811,2 (802,3–820,1)
	Sódio (mg)	2.408,6 (2.344,3–2.472,8)	2.349,7 (2.293,8–2.405,7)	2.227 (2.193,6–2.260,5)	2.180,2 (2.151,7–2.208,7)	1.983,3 (1.917,3–2.049,3)	1.921,2 (1.878,0–1.964,4)
	Potássio (mg)	2.100,3 (2.052,8–2.147,7)	1.906,2 (1.863,0–1.949,4)	2.140,5 (2.111,4–2.169,6)	1.972,3 (1.948,7–1.996,0)	2.109,0 (2.057,3–2.160,8)	1.948,3 (1.911,5–1.985,1)
	Cobre (mg)	1,2 (1,2–1,3)	1,2 (1,2–1,2)	1,2 (1,2–1,2)	1,2 (1,2–1,2)	1,1 (1,1–1,2)	1,2 (1,1–1,2)
	Zinco (mg)	10,2 (9,9–10,5)	9,6 (9,4–9,8)	10,1 (10,0–10,2)	9,5 (9,3–9,7)	9,5 (9,2–9,8)	8,6 (8,4–8,8)
	Vitamina A (mcg)^a^	400,2 (359,9–440,5)	319,6 (289,5–349,8)	410,4 (384,6–436,1)	342,2 (325,9–358,6)	450,8 (429,3–472,4)	412 (399,8–424,3)
	Tiamina (mg)	1,0 (0,9–1,0)	1,0 (1,0–1,0)	0,8 (0,8–0,9)	0,9 (0,8–0,9)	0,8 (0,7–0,8)	0,8 (0,8–0,8)
	Riboflavina (mg)	1,2 (1,2–1,3)	1,1 (1,0–1,1)	1,2 (1,1–1,2)	1,0 (0,9–1,0)	1,2 (1,1–1,2)	1,0 (1,0–1,0)
	Piridoxina (mg)	0,8 (0,7–0,8)	0,7 (0,7–0,7)	0,7 (0,6–0,7)	0,6 (0,6–0,6)	0,6 (0,5–0,6)	0,6 (0,6–0,6)
	Cobalamina (mcg)	4,7 (4,2–5,3)	3,8 (3,5–4,0)	4,6 (4,5–4,8)	3,6 (3,5–3,7)	4,4 (4,3–4,6)	3,3 (3,3–3,4)
	Vitamina D (mcg)^b^	2,2 (2,1–2,4)	1,8 (1,6–1,9)	1,8 (1,8–1,9)	1,4 (1,4–1,5)	1,8 (1,6–2,0)	1,3 (1,3–1,3)
	Vitamina E (mg)^c^	5,8 (5,6–6,0)	5,8 (5,4–6,1)	5,5 (5,4–5,6)	5,9 (5,7–6,1)	5,3 (5,3–5,4)	5,5 (5,3–5,6)
	Vitamina C (mg)	128,0 (120,7–135,2)	126,0 (117,6–134,4)	134,6 (131,7–137,6)	120,6 (117,6–123,6)	133,5 (122,4–144,7)	128,9 (121,2–136,6)
	Niacina (mg)^d^	13,8 (13,2–14,3)	14,6 (13,7–15,4)	13,7 (13,4–14,0)	14,4 (14,0–14,8)	12,6 (12,0–13,2)	13,0 (12,4–13,5)

a Equivalente de atividade de retinol (RAE).

b Ergocalciterol (D2) + colecalciterol (D3).

c Alfa-tocoferol total.

d Niacina pré-formada.

Prevalências de inadequação acima de 50% foram observadas para piridoxina, vitamina A e magnésio em todas as faixas etárias e, em específico, para fósforo entre adolescentes, tiamina entre adultos e idosos em ambos os inquéritos e, em 2017–2018, riboflavina entre homens idosos e adultos de ambos os sexos. Cálcio, vitamina D e vitamina E apresentaram as maiores inadequações em ambos os inquéritos (> 85%) (Tabela 2).

**Tabela 2:** Prevalências de inadequação (IC95%) da ingestão de vitaminas e minerais entre adolescentes, adultos e idosos nos Inquéritos Nacionais de Alimentação 2008-2009 e 2017–2018.

Nutrientes		Adolescentes		Adultos		Idosos	
		2008–2009	2017–2018	2008–2009	2017–2018	2008–2009	2017–2018
Homens							
	Cálcio (mg)	97,4 (96,9–97,9)	98,1 (97,9–98,4)	89,1 (88,0–90,2)	91,0 (90,1–91,8)	92,4 (91,0–93,8)	94,4 (93,6–95,1)
	Magnésio (mg)	50,2 (48,5–52,0)	54,2 (51,2–57,2)	66,1 (65,2–67,1)	69,2 (67,9–70,5)	77,0 (74,4–79,5)	80,5 (78,7–82,3)
	Fósforo (mg)	50,3 (47,8–52,8)	51,8 (49,9–53,7)	2,8 (2,3–3,4)	2,1 (1,8–2,5)	6,5 (5,7–7,4)	6,9 (6,4–7,5)
	Cobre (mg)	3,1 (2,7–3,6)	2,1 (1,7–2,4)	3,7 (3,2–4,1)	2,4 (2,1–2,7)	6,4 (4,7–8,1)	4,5 (4,0–5,0)
	Zinco (mg)	16,5 (13,9–19,0)	17,5 (14,7–20,3)	22,4 (21,1–23,6)	23,5 (22,4–24,7)	33,5 (30,8–36,1)	39,0 (37,7–40,2)
	Vitamina A (mcg)^a^	78,8 (75,4–82,3)	83,5 (81,1–85,9)	84,7 (82,9–86,4)	89,3 (88,4–90,2)	84,7 (82,9–86,6)	87,5 (86,1–89,0)
	Tiamina (mg)	41,1 (38,1–44,1)	32,0 (29,7–34,4)	59,8 (56,8–62,8)	51,8 (50,9–52,7)	70,0 (68,1–72,0)	63,6 (61,8–65,4)
	Riboflavina (mg)	31,4 (26,7–36,0)	36,6 (34,3–38,9)	45,9 (43,9–47,8)	55,0 (54,1–55,9)	47,9 (45,2–50,5)	57,9 (56,9–58,8)
	Piridoxina (mg)	70,2 (68,6–71,8)	68,8 (65,7–72,0)	81,5 (77,9–85,0)	82,3 (80,5–84,0)	95,9 (94,5–97,2)	95,0 (94,3–95,7)
	Cobalamina (mcg)	6,2 (4,3–8,1)	7,7 (6,4–9,1)	5,5 (4,3–6,6)	7,2 (6,2–8,1)	7,9 (6,2–9,6)	13,3 (11,9–14,7)
	Vitamina D (mcg)^b^	99,4 (99,2–99,6)	99,8 (99,8–99,9)	99,6 (99,4–99,8)	99,9 (99,9–100)	99,7 (99,6–99,9)	100 (99,9–100,0)
	Vitamina E (mg)^c^	90,6 (89,5–91,7)	89,1 (87,4–90,8)	93,6 (93,0–94,1)	91,6 (90,4–92,8)	96,1 (95,3–96,8)	95,3 (94,2–96,3)
	Vitamina C (mg)	33,2 (31,7–34,6)	34,5 (30,0–38,9)	43,7 (42,8–44,6)	47,5 (45,8–49,2)	45,3 (42,8–47,8)	48,7 (46,2–51,3)
Mulheres							
	Cálcio (mg)	98,0 (97,6–98,3)	99,0 (98,5–99,4)	93,7 (93,1–94,3)	96,1 (95,8–96,5)	97,5 (96,9–98,1)	98,5 (98,3–98,6)
	Magnésio (mg)	57,8 (54,2–61,4)	64,7 (62,5–66,9)	64,1 (63,3–64,9)	68,8 (66,9–70,7)	69,1 (68,0–70,1)	73,0 (71,8–74,1)
	Fósforo (mg)	64,0 (62,2–65,7)	71,1 (66,8–75,3)	11,5 (10,8–12,2)	12,8 (12,0–13,6)	15,0 (14,1–15,9)	18,7 (17,2–20,1)
	Cobre (mg)	7,3 (6,3–8,3)	6,6 (5,9–7,2)	13,1 (12,0–14,3)	10,6 (9,9–11,3)	15,0 (13,8–16,3)	11,9 (10,3–13,5)
	Zinco (mg)	21,1 (18,8–23,4)	25,6 (23,7–27,5)	18,1 (17,1–19,1)	22,2 (20,7–23,8)	23,0 (20,2–25,7)	31,4 (29,2–33,7)
	Vitamina A (mcg)^a^	69,2 (64,9–73,4)	78,9 (75,3–82,5)	72,4 (69,7–75,1)	80,1 (78,5–81,6)	68,0 (65,7–70,3)	72,3 (71,1–73,6)
	Tiamina (mg)	42,5 (40,2–44,8)	40,8 (39,3–42,3)	63,5 (62,4–64,6)	61,8 (60,6–63,1)	70,2 (67,3–73,1)	66,5 (65,8–67,2)
	Riboflavina (mg)	27,1 (22,2–31,9)	40,2 (34,4–46,1)	36,6 (34,5–38,6)	51,4 (50,0–52,8)	36,6 (32,6–40,6)	46,2 (44,7–47,8)
	Piridoxina (mg)	71,2 (68,6–73,8)	75,8 (72,6–78,9)	89,5 (87,8–91,3)	91,8 (91,1–92,5)	96,4 (95,1–97,8)	96,7 (96,2–97,2)
	Cobalamina (mcg)	7,6 (4,4–10,8)	11,4 (7,6–15,3)	10,9 (9,2–12,5)	17,1 (15,5–18,8)	12,6 (11,1–14,0)	21,3 (18,2–24,4)
	Vitamina D (mcg)^b^	99,5 (99,3–99,6)	99,9 (99,8–100,0)	99,8 (99,7–99,9)	100,0 (99,9–100)	99,8 (99,6–100,0)	100,0 (100,0–100,0)
	Vitamina E (mg)^c^	94,1 (93,2–94,9)	93,9 (92,1–95,6)	98,1 (97,9–98,4)	97,0 (96,1–97,8)	98,4 (98,2–98,6)	98,0 (97,4–98,6)
	Vitamina C (mg)	29 (26,3–31,7)	29,4 (25,7–33,1)	34,4 (33,5–35,2)	38,5 (37,1–39,9)	34,7 (31,6–37,9)	35,7 (32,9–38,6)

a Equivalente de atividade de retinol (RAE).

b Ergocalciterol (D2) + colecalciterol (D3).

c Alfa-tocoferol total.

As prevalências de inadequação para a maioria dos micronutrientes foram maiores em 2017–2018 que em 2008–2009. As mais importantes diferenças (> 10%) encontradas na população feminina foram para riboflavina e zinco em todas as faixas de idade e riboflavina entre homens adultos e idosos. De forma contrária, tiamina entre as mulheres idosas e homens de todas as idades, e cobre entre as mulheres adultas e idosas e entre os homens adolescentes e adultos foram os únicos nutrientes que mostraram redução discreta (< 10 pontos percentuais) na prevalência de inadequação no período investigado (Tabela 2).

Em 2008–2009, a porcentagem de indivíduos cuja ingestão de sódio deveria ser diminuída para reduzir o risco de doenças crônicas foi, entre os homens, de 75% (72%–78%) em adolescentes, 71% (70%–72%) em adultos, e 52% (47%–56%) em idosos. Entre as mulheres, essas porcentagens foram de 61% (59%–63%), 41% (39%–43%) e 29% (28%–30%), respectivamente. Em 2017–2018, os percentuais foram, para o sexo masculino, 78% (76%–80%) em adolescentes, 74% (73%–75%) em adultos, e 52% (50%–54%) em idosos. Entre as mulheres, 59% (56%–61%), 39% (36%–41%) e 25% (24%–27%).

Tanto em 2008–2009 (Tabelas 3 e 4) quanto em 2017–2018 (Tabelas 5 e 6), houve redução das prevalências de inadequação dos micronutrientes com o aumento da renda média per capita. No último inquérito, excetuando as estimativas relativas a cobre, magnésio e vitamina D entre idosos, e vitamina E entre mulheres adolescentes, diferenças significantes foram observadas entre os dois extremos de renda per capita (< 0,5 *vs*. > 2 salários-mínimos), sendo as mais expressivas (> 15 pontos percentuais) para vitamina C, vitamina A, riboflavina e tiamina em todas as idades, fósforo e piridoxina entre adolescentes, e cálcio entre homens adultos (Tabelas 5 e 6).

**Tabela 3:** Prevalências de inadequação (IC95%) da ingestão de vitaminas e minerais segundo renda familiar média per capita entre homens no Inquérito Nacional de Alimentação (INA/POF) 2008–2009.

Nutrientes	Faixa etária	Renda per capita			
		< 0,5 SM	0,5–1 SM	1–2 SM	> 2 SM
		% (IC95%)	% (IC95%)	% (IC95%)	% (IC95%)
Cálcio (mg)					
	Adolescentes	99,3 (98,9–99,6)	98,5 (98,3–98,8)	97,2 (96,4–98,0)	92,4 (90,9–94,0)
	Adultos	96,7 (96,1–97,3)	94,2 (93,8–94,7)	90,7 (89,6–91,8)	80,7 (78,5–83,0)
	Idosos	98,4 (97,9–98,9)	96,8 (96,1–97,4)	95,1 (94,5–95,7)	87,4 (85,1–89,7)
Magnésio (mg)					
	Adolescentes	50,0 (45,2–54,8)	51,0 (48,1–53,9)	48,1 (45,6–50,5)	53,3 (50,3–56,2)
	Adultos	71,1 (69,1–73,1)	65,3 (62,8–67,7)	63,6 (62,5–64,7)	66,6 (64,4–68,9)
	Idosos	81,4 (77,0–85,9)	76,4 (72,4–80,4)	76,1 (72,3–79,9)	77,6 (76,1–79,0)
Fósforo (mg)					
	Adolescentes	62,2 (60,0–64,3)	50,3 (48,1–52,6)	45,4 (42,6–48,1)	36,1 (33,5–38,7)
	Adultos	5,6 (4,9–6,3)	3,3 (2,4–4,2)	2,5 (2,1–3,0)	1,5 (1,1–1,9)
	Idosos	13,4 (10,8–16,1)	8,2 (6,7–9,7)	7,2 (5,6–8,7)	4,1 (3,6–4,6)
Cobre (mg)					
	Adolescentes	3,6 (2,7–4,4)	2,9 (2,5–3,4)	2,7 (2,1–3,3)	3,5 (3,0–3,9)
	Adultos	4,6 (3,7–5,5)	3,3 (2,9–3,8)	3,3 (2,7–4,0)	3,7 (3,1–4,4)
	Idosos	8,4 (5,5–11,4)	6,0 (4,2–7,7)	6,4 (4,0–8,8)	6,3 (5,0–7,5)
Zinco (mg)					
	Adolescentes	21,4 (16,2–26,6)	15,3 (13,9–16,7)	14,0 (12,1–16,0)	14,0 (12,6–15,4)
	Adultos	30,3 (28,2–32,4)	22,1 (19,7–24,5)	20,8 (19,7–22,0)	20,1 (18,2–22,0)
	Idosos	44,4 (40,4–48,4)	34,0 (31,3–36,7)	34,3 (31,6–37,0)	31,0 (26,6–35,4)
Vitamina A (mcg)^a^					
	Adolescentes	87,0 (82,1–91,9)	81,6 (79,0–84,3)	76,0 (73,8–78,2)	61,6 (58,7–64,6)
	Adultos	95,2 (93,4–96,9)	90,2 (88,7–91,7)	86,6 (84,7–88,4)	73,9 (70,9–76,9)
	Idosos	96,1 (93,9–98,2)	92,3 (90,6–93,9)	88,8 (87,5–90,2)	77,1 (74,3–79,9)
Tiamina (mg)					
	Adolescentes	52,8 (45,3–60,3)	41,6 (38,6–44,6)	34,5 (32,6–36,5)	28,5 (25,9–31,1)
	Adultos	78,0 (74,9–81,0)	64,4 (60,9–67,8)	57,9 (55,8–60,0)	49,6 (43,9–55,2)
	Idosos	87,8 (84,4–91,3)	77,2 (74,4–80,1)	72,0 (68,0–76,0)	62,8 (59,9–65,8)
Riboflavina (mg)					
	Adolescentes	42,8 (35,6–50,0)	30,6 (26,3–34,9)	25,5 (22,8–28,3)	20,1 (17,7–22,6)
	Adultos	65,7 (61,1–70,2)	49,3 (46,9–51,7)	43,8 (42,3–45,2)	35,7 (32,9–38,6)
	Idosos	71,3 (68,1–74,5)	54,9 (50,9–58,9)	49,9 (47,7–52,2)	39,8 (36,1–43,6)
Piridoxina (mg)					
	Adolescentes	81,5 (78,1–84,8)	71,3 (69,1–73,4)	64,6 (62,3–67,0)	55,2 (52,7–57,6)
	Adultos	92,9 (91,1–94,6)	85,1 (82,1–88,1)	81,4 (77,9–84,8)	73,6 (68,2–79,0)
	Idosos	99,5 (99,2–99,8)	97,8 (97,2–98,5)	97,0 (96,3–97,8)	94,0 (92,5–95,6)
Cobalamina (mcg)					
	Adolescentes	7,6 (5,2–9,9)	6,6 (4,7–8,5)	5,4 (3,9–7,0)	3,8 (2,6–4,9)
	Adultos	8,1 (6–10,3)	6,4 (5,1–7,8)	5,5 (4,4–6,7)	3,5 (2,5–4,6)
	Idosos	12,8 (10,9–14,6)	9,8 (7,7–12)	9,2 (7,7–10,7)	5,3 (3,5–7,1)
Vitamina D (mcg)^b^					
	Adolescentes	99,5 (99,3–99,7)	99,5 (99,4–99,6)	99,3 (99,1–99,6)	98,9 (98,5–99,2)
	Adultos	99,7 (99,6–99,9)	99,7 (99,5–99,8)	99,7 (99,5–99,8)	99,3 (99,0–99,7)
	Idosos	99,9 (99,7–100)	99,8 (99,7–100)	99,7 (99,6–99,9)	99,6 (99,3–99,9)
Vitamina E (mg)^c^					
	Adolescentes	91,5 (89,2–93,7)	90,3 (89,2–91,4)	89,8 (88,6–91,1)	90,1 (89,3–90,9)
	Adultos	95,3 (94,7–95,8)	93,3 (92,0–94,6)	93,4 (93,2–93,7)	93,1 (92,1–94,1)
	Idosos	97,3 (96,5–98,2)	96,0 (95,3–96,7)	96,4 (95,9–96,8)	95,7 (94,4–96,9)
Vitamina C (mg)					
	Adolescentes	43,8 (40,6–46,9)	35,2 (33,4–36,9)	27,5 (25,9–29,1)	17,8 (16,1–19,5)
	Adultos	64,2 (61,3–67,2)	51,5 (49,3–53,6)	43,1 (41,2–45,1)	29,3 (27,4–31,2)
	Idosos	69,8 (65,6–74,0)	57,3 (54,6–59,9)	48,7 (46,3–51,2)	33,2 (30,1–36,3)

a Equivalente de atividade de retinol (RAE).

b Ergocalciterol (D2) + colecalciterol (D3).

c Alfa-tocoferol total.

**Tabela 4:** Prevalências de inadequação (IC95%) da ingestão de vitaminas e minerais segundo renda média familiar per capita entre mulheres no Inquérito Nacional de Alimentação (INA/POF) 2008–2009.

Nutrientes	Faixa etária	Renda per capita			
		< 0,5 SM	0,5–1 SM	1–2 SM	> 2 SM
		% (IC95%)	% (IC95%)	% (IC95%)	% (IC95%)
Cálcio (mg)					
	Adolescentes	99,3 (99,1–99,5)	98,9 (98,7–99,1)	97,9 (97,5–98,2)	94,2 (93,3–95,1)
	Adultos	98,2 (97,9–98,6)	96,9 (96,6–97,2)	94,7 (94,1–95,2)	87,8 (86,3–89,4)
	Idosos	99,6 (99,6–99,7)	99,3 (99,2–99,4)	98,6 (98,3–99,0)	96,0 (94,8–97,2)
Magnésio (mg)					
	Adolescentes	58,9 (55,8–62,0)	56,4 (52,4–60,4)	56,6 (52,0–61,3)	60,8 (52,9–68,7)
	Adultos	69,5 (67,4–71,5)	63,3 (61,3–65,3)	61,5 (60,3–62,7)	64,5 (62,5–66,5)
	Idosos	73,3 (70,8–75,9)	69,2 (66,9–71,5)	67,1 (65,2–69,1)	69,9 (68,3–71,6)
Fósforo (mg)					
	Adolescentes	73,2 (70,6–75,8)	65,1 (63,5–66,8)	59,5 (56,7–62,3)	51,8 (49,8–53,8)
	Adultos	19,1 (17,5–20,8)	13,3 (12,0–14,6)	10,4 (9,4–11,3)	7,3 (6,9–7,7)
	Idosos	26,5 (25,0–28,0)	19,7 (17,6–21,8)	15,7 (14,1–17,4)	11,1 (10,1–12)
Cobre (mg)					
	Adolescentes	8,1 (6,4–9,8)	6,7 (5,9–7,5)	6,8 (5,7–7,9)	7,6 (6,4–8,8)
	Adultos	15,7 (13,4–17,9)	12,6 (11,8–13,4)	12,1 (10,1–14,1)	13,2 (12,5–14,0)
	Idosos	17,7 (14,6–20,7)	14,8 (13,1–16,5)	14,4 (12,4–16,4)	15,3 (13,8–16,9)
Zinco (mg)					
	Adolescentes	26,6 (22,1–31,2)	19,9 (18,9–20,8)	18,4 (15,7–21,0)	17,7 (16,1–19,3)
	Adultos	25,1 (22,3–27,9)	18,0 (16,5–19,4)	16,6 (15,4–17,7)	16,2 (15,4–17,0)
	Idosos	31,9 (26,3–37,5)	24,4 (21,4–27,4)	22,5 (18,9–26,0)	21,6 (19,6–23,7)
Vitamina A (mcg)^a^					
	Adolescentes	81,4 (75,9–86,9)	71,6 (68,6–74,7)	65,6 (63,4–67,7)	48,0 (44,7–51,3)
	Adultos	88,1 (84,8–91,4)	79,6 (77,3–81,9)	74,3 (71,8–76,8)	57,1 (52,8–61,3)
	Idosos	88,2 (84,4–91,9)	79,8 (77,7–81,8)	73,6 (71,4–75,9)	56,7 (53,9–59,5)
Tiamina (mg)					
	Adolescentes	55,5 (50,0–61,1)	41,9 (39,8–44,0)	36,1 (34,0–38,3)	29,4 (25,9–32,9)
	Adultos	80,5 (77,4–83,7)	67,6 (65,3–70,0)	61,4 (60,1–62,8)	53,3 (50,5–56,2)
	Idosos	88,5 (85,8–91,2)	78,2 (74,5–81,9)	71,7 (69,4–74,0)	64,4 (59,4–69,3)
Riboflavina (mg)					
	Adolescentes	39,6 (32,8–46,4)	25,6 (20,8–30,5)	21,3 (18,1–24,6)	15,5 (11,5–19,6)
	Adultos	56,5 (51,9–61,0)	39,6 (36,4–42,8)	34,0 (32,9–35,1)	26,6 (24,2–28,9)
	Idosos	60,5 (53,3–67,6)	44,0 (40,3–47,6)	37,4 (33,9–40,9)	29,7 (25,7–33,7)
Piridoxina (mg)					
	Adolescentes	82,9 (80,4–85,5)	71,5 (67,8–75,2)	66,0 (61,8–70,2)	56,0 (50,1–61,9)
	Adultos	96,7 (95,8–97,6)	92,1 (90,4–93,7)	89,5 (87,6–91,4)	84,0 (81,4–86,6)
	Idosos	99,5 (99,4–99,7)	98,3 (97,6–99,0)	97,4 (96,5–98,3)	95,3 (93,4–97,1)
Cobalamina (mcg)					
	Adolescentes	9,2 (5,7–12,7)	8,1 (4,3–11,9)	6,9 (4,7–9,2)	4,5 (2,4–6,6)
	Adultos	15,2 (12,8–17,7)	12,7 (10,4–15,1)	10,7 (9,2–12,2)	7,5 (6,1–8,8)
	Idosos	18,9 (16,0–21,9)	16,4 (14,2–18,6)	13,6 (12,4–14,9)	9,4 (7,4–11,3)
Vitamina D (mcg)^b^					
	Adolescentes	99,6 (99,4–99,7)	99,6 (99,5–99,7)	99,5 (99,3–99,7)	99,1 (98,8–99,3)
	Adultos	99,9 (99,8–100)	99,8 (99,8–99,9)	99,8 (99,8–99,9)	99,6 (99,5–99,8)
	Idosos	99,9 (99,8–100)	99,9 (99,8–100)	99,8 (99,8–99,9)	99,7 (99,3–100,1)
Vitamina E (mg)^c^					
	Adolescentes	94,5 (93,5–95,6)	93,7 (92,1–95,3)	94,0 (93,0–94,9)	93,9 (92,1–95,7)
	Adultos	98,6 (98,5–98,8)	98,1 (97,6–98,6)	98,1 (97,9–98,2)	97,9 (97,6–98,2)
	Idosos	99,1 (98,8–99,4)	98,5 (98,0–99,0)	98,4 (98,3–98,6)	98,3 (98,1–98,6)
Vitamina C (mg)					
	Adolescentes	41,2 (36,9–45,5)	30,1 (26,5–33,6)	23,3 (19,1–27,5)	13,5 (10,3–16,7)
	Adultos	54,1 (51,3–56,9)	41,1 (39,1–43,0)	32,9 (31,4–34,5)	20,8 (18,6–23,0)
	Idosos	58,6 (53,8–63,4)	46,3 (42,6–49,9)	37,4 (32,9–41,8)	24,3 (21,4–27,2)

a Equivalente de atividade de retinol (RAE).

b Ergocalciterol (D2) + colecalciterol (D3).

c Alfa-tocoferol total.

**Tabela 5:** Prevalências de inadequação (IC95%) da ingestão de vitaminas e minerais segundo renda familiar per capita entre homens no Inquérito Nacional de Alimentação 2017–2018.

Nutrientes	Faixa etária	Renda per capita			
		< 0.5 SM	0.5–1 SM	1–2 SM	> 2 SM
Cálcio (mg)					
	Adolescentes	99,6 (99,5–99,8)	99,0 (98,9–99,2)	97,8 (97,3–98,3)	93,1 (92,1–94,2)
	Adultos	98,1 (97,7–98,5)	95,8 (95,2–96,4)	91,7 (90,4–93,0)	81,9 (79,8–83,9)
	Idosos	99,2 (99,0–99,5)	98,2 (97,8–98,7)	96,6 (95,5–97,6)	90,2 (89,1–91,3)
Magnésio (mg)					
	Adolescentes	57,4 (54,7–60,1)	51,8 (48,6–54,9)	53,9 (47,7–60,0)	53,1 (45,4–60,9)
	Adultos	73,9 (70,1–77,7)	67,4 (65,6–69,3)	68,4 (64,7–72,0)	69,1 (66,8–71,4)
	Idosos	83,7 (81,2–86,3)	78,9 (77,7–80,1)	80,9 (77,9–83,8)	80,5 (78,7–82,2)
Fósforo (mg)					
	Adolescentes	63,1 (59,7–66,4)	51,8 (48,6–55,0)	45,5 (41–50,0)	38,3 (35,0–41,5)
	Adultos	4,5 (2,9–6,0)	2,3 (1,9–2,7)	1,8 (1,6–2,0)	1,1 (0,8–1,3)
	Idosos	14 (11,9–16,1)	9,0 (8,3–9,7)	7,2 (6,4–8,1)	4,5 (4–5,1)
Cobre (mg)					
	Adolescentes	2,2 (1,7–2,6)	1,8 (1,4–2,1)	2,3 (1,6–3,1)	2,2 (1,5–3,0)
	Adultos	2,4 (1,6–3,2)	2,0 (1,6–2,3)	2,5 (2,1–3,0)	2,7 (2,3–3,1)
	Idosos	4,1 (2,7–5,5)	3,6 (3,0–4,1)	4,7 (3,9–5,6)	4,7 (4,3–5,1)
Zinco (mg)					
	Adolescentes	20,6 (17,0–24,3)	17,6 (14,3–20,9)	15,5 (14,0–16,9)	14,1 (11,7–16,5)
	Adultos	28,7 (26,4–31,1)	24,6 (22,8–26,5)	22,3 (20,3–24,3)	21,0 (18,5–23,5)
	Idosos	45,3 (41,9–48,8)	41,1 (38,1–44,1)	39,4 (37,6–41,2)	36,5 (35,3–37,7)
Vitamina A (mcg)^a^					
	Adolescentes	92,7 (90,2–95,3)	85,8 (82,3–89,3)	78,6 (73,7–83,4)	64,6 (60,2–69,0)
	Adultos	97,7 (97,1–98,3)	94,3 (93,6–95,0)	89,3 (87,8–90,9)	79,7 (78,1–81,3)
	Idosos	98,1 (97,7–98,6)	94,7 (93,7–95,8)	90,2 (88,6–91,8)	80,7 (78,8–82,6)
Tiamina (mg)					
	Adolescentes	42,6 (37,7–47,6)	30,1 (27,6–32,6)	26,5 (24,6–28,5)	23,2 (21,1–25,3)
	Adultos	67,4 (63,7–71,2)	53,3 (51,9–54,6)	48,8 (45,8–51,8)	45,2 (43,2–47,2)
	Idosos	79,7 (77,3–82,1)	67,6 (65,3–69,9)	63,9 (59,7–68)	59,5 (58,2–60,7)
Riboflavina (mg)					
	Adolescentes	46,9 (44,2–49,6)	35,9 (32,0–39,8)	31,3 (27,0–35,7)	25,1 (22,7–27,5)
	Adultos	70,4 (68,4–72,4)	58,3 (56,4–60,3)	52,8 (49,8–55,8)	46,1 (43,6–48,6)
	Idosos	75,7 (73,6–77,7)	64,1 (62,6–65,5)	58,8 (56,1–61,5)	51,7 (48,5–54,9)
Piridoxina (mg)					
	Adolescentes	77,5 (75,6–79,4)	68,6 (63,4–73,9)	64,7 (58,8–70,5)	57,0 (53,1–60,8)
	Adultos	90,2 (88,1–92,3)	84,2 (82,0–86,4)	81,5 (79,9–83,0)	77,0 (74,0–79,9)
	Idosos	98,5 (98,2–98,7)	96,7 (95,7–97,6)	95,7 (94,8–96,6)	93,3 (92,4–94,1)
Cobalamina (mcg)					
	Adolescentes	9,2 (7,3–11,1)	9,0 (7,6–10,5)	6,3 (5,3–7,3)	2,6 (1,7–3,5)
	Adultos	10,4 (9,2–11,7)	10,0 (8,5–11,5)	7,0 (5,1–8,8)	3,4 (3,0–3,8)
	Idosos	19,8 (17,8–21,8)	19,8 (17,8–21,8)	15,1 (12,2–18)	7,7 (6,9–8,4)
Vitamina D (mcg)^b^					
	Adolescentes	99,9 (99,9–100)	99,9 (99,9–99,9)	99,8 (99,7–99,8)	99,3 (99–99,5)
	Adultos	100 (100–100)	100 (100–100)	99,9 (99,9–100)	99,8 (99,7–99,9)
	Idosos	100 (100–100)	100 (100–100)	100 (100–100)	99,9 (99,9– 100)
Vitamina E (mg)^c^					
	Adolescentes	91,4 (89,7–93,1)	89,4 (87,7–91)	88,3 (85–91,6)	84,6 (81,0–88,3)
	Adultos	94,4 (92,7–96,2)	92,7 (91,7–93,8)	91,4 (89,5–93,3)	89,2 (88,0–90,5)
	Idosos	97,2 (96,0–98,5)	96,2 (95,3–97,2)	95,8 (94,6–97)	94,0 (92,9–95,1)
Vitamina C (mg)					
	Adolescentes	47,7 (41,0–54,5)	34,1 (31,4–36,7)	27,6 (24,9–30,2)	17,8 (16,0–19,7)
	Adultos	68,0 (63,9–72,2)	53,2 (51,7–54,7)	45,5 (44,3–46,6)	33,3 (32,1–34,5)
	Idosos	72,8 (70,0–75,5)	58,5 (55,0–62,1)	51,3 (48,7–53,8)	38,0 (34,6–41,3)

SM: salário-mínimo.

a Equivalente de atividade de retinol (RAE).

b Ergocalciterol (D2) + colecalciterol (D3).

c Alfa-tocoferol total.

**Tabela 6:** Prevalências de inadequação (IC95%) da ingestão de vitaminas e minerais segundo renda familiar per capita entre mulheres no Inquérito Nacional de Alimentação 2017–2018.

Nutrientes	Faixa etária	Renda per capita			
		< 0.5 SM	0.5–1 SM	1–2 SM	> 2 SM
Cálcio (mg)					
	Adolescentes	99,8 (99,7– 99,9)	99,5 (99,2–99,8)	98,8 (98,0–99,6)	96,1 (95,0–97,1)
	Adultos	99,3 (99,2–99,4)	98,4 (97,9–98,9)	96,5 (95,3–97,8)	91,3 (90,6–92,0)
	Idosos	99,9 (99,9–100)	99,7 (99,7–99,8)	99,2 (99,0–99,5)	97,3 (96,9–97,6)
Magnésio (mg)					
	Adolescentes	65,9 (61,7–70,2)	63,0 (60,7–65,3)	65,5 (58,9–72,1)	63,9 (58,9–68,8)
	Adultos	73,2 (69,9–76,5)	67,2 (65,6–68,7)	67,9 (63,7–72,1)	68,6 (65,4–71,8)
	Idosos	77,3 (71,6–83,0)	72,1 (68,4–75,8)	73,0 (71,3–74,7)	72,8 (72,3–73,4)
Fósforo (mg)					
	Adolescentes	80,3 (77,4–83,2)	70,9 (66,4–75,3)	66,2 (59,4–73,0)	57,7 (51,5–64,0)
	Adultos	20,9 (18,3–23,5)	13,9 (12,8–15,0)	11,3 (9,7–12,9)	8,2 (7,2–9,2)
	Idosos	32,5 (26,4–38,7)	23,1 (21,8–24,4)	19,0 (17,9–20,2)	13,8 (12,5–15,1)
Cobre (mg)					
	Adolescentes	6,2 (5,1–7,3)	5,9 (5–6,7)	7,3 (5,4–9,2)	7,6 (5,1–10,1)
	Adultos	10,5 (8,1–13)	9,2 (8,2–10,2)	11,0 (10,2–11,9)	11,6 (10,7–12,4)
	Idosos	11,6 (7,7–15,4)	10,1 (7,9–12,4)	12,1 (10,2–14,0)	12,3 (11,1–13,6)
Zinco (mg)					
	Adolescentes	29,2 (26,7–31,7)	25,5 (23,6–27,4)	23,2 (20,3–26,1)	21,4 (17,5–25,3)
	Adultos	26,5 (24,0–28,9)	23,3 (21,6–25,0)	21,0 (18,6–23,4)	20,0 (17,8–22,2)
	Idosos	38,7 (34,6–42,7)	34,6 (32,5–36,7)	31,3 (28,4–34,1)	29,3 (27,0–31,7)
Vitamina A (mcg)^a^					
	Adolescentes	89,9 (87,3–92,4)	81,5 (78,7–84,4)	72,3 (66,8–77,9)	55,6 (50,9–60,3)
	Adultos	93,9 (92,9–95,0)	87,2 (86,4–88,0)	78,9 (77,3–80,5)	64,8 (62,2–67,5)
	Idosos	92,4 (91,4–93,5)	85,0 (83,7–86,2)	75,9 (74,1–77,8)	60,4 (58,4–62,4)
Tiamina (mg)					
	Adolescentes	50,3 (45,1–55,4)	39,2 (37,3–41,0)	35,8 (32,4–39,2)	31,2 (29,4–32,9)
	Adultos	75,3 (72,3–78,4)	63,2 (61,0–65,4)	58,7 (54,9–62,5)	55,3 (53,5–57,2)
	Idosos	82,0 (78,8–85,3)	70,9 (69,4–72,3)	66,8 (65,0–68,5)	62,5 (60,5–64,4)
Riboflavina (mg)					
	Adolescentes	50,6 (45,6–55,5)	39,5 (33,2–45,9)	34,7 (26,7–42,7)	28,1 (23,9–32,3)
	Adultos	66,5 (63,6–69,3)	54,6 (51,9–57,4)	48,5 (44,2–52,8)	42,0 (40,4–43,6)
	Idosos	65,8 (63,3–68,4)	53,3 (51,0–55,6)	46,9 (43,5–50,2)	39,7 (37,6–41,9)
Piridoxina (mg)					
	Adolescentes	82,3 (80,3–84,4)	75,9 (72,6–79,1)	72,7 (66,6–78,9)	64,8 (62,0–67,7)
	Adultos	96,1 (95,4–96,7)	93,2 (92,0–94,3)	91,4 (89,9–92,8)	88,4 (87,0–89,7)
	Idosos	99,0 (98,7–99,2)	98,0 (97,5–98,6)	97,2 (96,4–98,0)	95,5 (94,8–96,2)
Cobalamina (mcg)					
	Adolescentes	13,1 (9,8–16,3)	13,3 (9,5–17,1)	9,9 (5,0–14,9)	5,1 (2,9–7,3)
	Adultos	22,4 (20,1–24,7)	22,4 (19,9–24,8)	16,9 (13,6–20,1)	9,6 (8,6–10,5)
	Idosos	30,9 (26,2–35,6)	30,5 (26,1–34,8)	23,5 (18,4–28,5)	13,7 (12,2–15,2)
Vitamina D (mcg)^b^					
	Adolescentes	100 (99,9–100)	99,9 (99,9–100)	99,9 (99,8–100)	99,6 (99,5–99,7)
	Adultos	100 (100–100)	100 (100–100)	100 (100–100)	99,9 (99,9–99,9)
	Idosos	100 (100–100)	100 (100–100)	100 (100–100)	100 (99,9–100)
Vitamina E (mg)^c^					
	Adolescentes	94,9 (93,2–96,6)	94,1 (93,0–95,3)	93,6 (90,3–96,8)	91,7 (89,4–93,9)
	Adultos	98,2 (97,6–98,8)	97,5 (96,9–98,1)	96,9 (95,6–98,1)	95,8 (94,7–96,8)
	Idosos	98,9 (98,4–99,5)	98,7 (98,2–99,2)	98,3 (97,5–99,0)	97,5 (96,8–98,2)
Vitamina C (mg)					
	Adolescentes	41,4 (33,7–49,1)	28,6 (25,7–31,6)	22,9 (20,1–25,8)	14,1 (12,2–16,1)
	Adultos	58,1 (53,4–62,8)	43,2 (41,5–44,9)	35,6 (34,1–37,1)	24,6 (23,2–26,0)
	Idosos	60,2 (54,5–65,9)	45,0 (42,1–47,9)	37,4 (34,3–40,5)	25,8 (23,6–27,9)

SM: salário-mínimo.

a Equivalente de atividade de retinol (RAE).

b Ergocalciterol (D2) + colecalciterol (D3).

c Total de alfa-tocoferol.

Quando as análises foram estratificadas pelas regiões do país, resultados semelhantes foram encontrados para homens e mulheres, tanto em 2008–2009 (Tabelas 7 e 8) quanto em 2017–2018 (Tabelas 9 e 10). Verificou-se que tiamina, zinco, vitamina C, magnésio, cobalamina e cobre se destacaram como os micronutrientes mais variáveis entre as regiões (Figuras 1 e 2). Em 2017–2018, as regiões Norte e Nordeste, seguidas por Centro-Oeste, destacaram-se entre a primeira e a segunda posições de maior prevalência de inadequação para a maioria dos nutrientes analisados. Entre as diferenças estatisticamente significativas, optou-se por destacar aquelas mais evidentes (> 15 pontos percentuais).

**Tabela 7:** Prevalências de inadequação (IC95%) da ingestão de vitaminas e minerais segundo região entre homens no Inquérito Nacional de Alimentação (INA/POF) 2008–2009.

Nutrientes	Faixa etária	Região				
		Norte	Nordeste	Sul	Sudeste	Centro-oeste
		% (IC95%)	% (IC95%)	% (IC95%)	% (IC95%)	% (IC95%)
Cálcio (mg)						
	Adolescentes	96,8 (96,3–97,3)	98,6 (98,2–99,0)	96,8 (95,8–97,7)	96,5 (95,7–97,3)	98,5 (98,1–98,8)
	Adultos	87,5 (86,4–88,5)	93,3 (92,7–93,9)	87,1 (84,8–89,4)	86,7 (85,7–87,8)	92,6 (92,1–93,0)
	Idosos	91,0 (89,7–92,2)	95,6 (94,8–96,5)	90,8 (88,3–93,3)	90,7 (89,1–92,3)	95,0 (94,1–96,0)
Magnésio (mg)						
	Adolescentes	47,5 (42,9–52,2)	56,5 (54,3–58,7)	46,6 (45,1–48,1)	52,0 (48,5–55,4)	46,5 (41,8–51,1)
	Adultos	64,6 (63,0–66,2)	72,8 (70,9–74,7)	62,0 (60,4–63,6)	68,1 (65,9–70,2)	65,1 (63,6–66,7)
	Idosos	75,5 (70,6–80,4)	82,7 (78,6–86,7)	73,4 (71,4–75,5)	78,7 (74,5–82,9)	76,1 (72,4–79,9)
Fósforo (mg)						
	Adolescentes	44,4 (41,8–47,1)	55,0 (52,2–57,8)	46,7 (44,7–48,7)	52,0 (48,8–55,2)	56,0 (52,5–59,5)
	Adultos	1,7 (1,5–2,0)	3,5 (3,1–3,9)	2,4 (1,7–3,1)	3,1 (2,6–3,7)	3,6 (3,1–4,1)
	Idosos	4,6 (3,7–5,6)	8,3 (6,6–9,9)	5,2 (4,5–5,9)	7,0 (5,6–8,3)	8,4 (6,8–10,1)
Cobre (mg)						
	Adolescentes	3,6 (2,8–4,5)	3,5 (3,0–4,0)	2,5 (2,2–2,8)	3,9 (2,7–5,1)	2,4 (2,0–2,9)
	Adultos	4,4 (3,5–5,2)	4,3 (3,8–4,9)	2,9 (2,6–3,3)	4,7 (3,7–5,8)	2,7 (2,4–3,1)
	Idosos	7,6 (5,5–9,7)	7,8 (5,6–10,0)	4,9 (3,7–6,2)	8,2 (5,3–11,1)	4,8 (3,1–6,6)
Zinco (mg)						
	Adolescentes	14,1 (10,6–17,5)	16,7 (13,8–19,6)	17,9 (15,3–20,4)	15,7 (11,1–20,2)	12,4 (9,6–15,3)
	Adultos	19,1 (17,0–21,2)	22,4 (21,2–23,7)	24,3 (23,2–25,3)	20,9 (16,8–25,0)	17,2 (15,5–18,9)
	Idosos	29,4 (26,6–32,2)	33,9 (30,6–37,2)	35,0 (31,7–38,4)	31,9 (26,6–37,2)	27,5 (24,6–30,5)
Vitamina A (mcg)^a^						
	Adolescentes	83,1 (80,1–86,2)	83,7 (80,6–86,7)	75,2 (72,2–78,1)	74,6 (70,2–79,0)	79,1 (75,4–82,7)
	Adultos	89,0 (87,3–90,7)	89,0 (87,5–90,5)	82,0 (80,4–83,6)	81,5 (78,6–84,5)	86,1 (84,4–87,8)
	Idosos	89,2 (87,2–91,2)	89,6 (87,4–91,7)	82,1 (80,4–83,9)	82,3 (79,9–84,8)	86,5 (84,4–88,6)
Tiamina (mg)						
	Adolescentes	49,9 (47,0–52,7)	48,2 (43,0–53,5)	35,3 (33,1–37,5)	32,0 (28,8–35,1)	48,0 (44,9–51,2)
	Adultos	69,5 (67,0–71,9)	67,6 (63,6–71,5)	54,7 (49,9–59,6)	51,3 (49,1–53,4)	68,8 (65,6–72,0)
	Idosos	78,9 (76,4–81,4)	77,7 (74,0–81,4)	65,4 (63,6–67,3)	62,2 (57,9–66,5)	78,3 (75,0–81,6)
Riboflavina (mg)						
	Adolescentes	38,9 (34,2–43,6)	34,7 (27,6–41,7)	28,1 (24,6–31,6)	25,7 (21,3–30,2)	36,0 (31,6–40,3)
	Adultos	54,9 (51,4–58,4)	49,9 (44,2–55,5)	42,4 (40,6–44,3)	40,2 (36,3–44,2)	53,3 (49,5–57,1)
	Idosos	57,4 (53,8–61,0)	53,0 (47,8–58,3)	44,4 (42,2–46,7)	42,8 (37,6–47,9)	56,8 (53,1–60,4)
Piridoxina (mg)						
	Adolescentes	75,9 (73,4–78,4)	75,2 (71,4–78,9)	66,4 (63,2–69,7)	62,4 (59,8–65,0)	76,6 (73,0–80,1)
	Adultos	85,9 (82,8–89,0)	85,2 (81,8–88,5)	79,2 (73,7–84,8)	76,2 (73,5–78,9)	87,2 (83,6–90,7)
	Idosos	97,6 (96,7–98,5)	97,3 (96,2–98,3)	94,9 (92,8–97,0)	94,1 (93,0–95,1)	97,8 (96,8–98,8)
Cobalamina (mcg)						
	Adolescentes	1,0 (0,5–1,5)	5,8 (3,9–7,7)	7,5 (5,4–9,6)	6,8 (3,5–10,1)	5,5 (3,6–7,5)
	Adultos	0,7 (0,4–1,0)	5,0 (3,9–6,0)	6,4 (5,0–7,8)	5,7 (3,7–7,8)	4,5 (3,6–5,5)
	Idosos	1,2 (0,6–1,8)	7,5 (6,1–8,9)	8,5 (6,3–10,8)	8,0 (5,5–10,6)	6,6 (5,0–8,3)
Vitamina D (mcg)^b^						
	Adolescentes	98,5 (97,9–99,0)	99,5 (99,3–99,6)	99,4 (99,2–99,6)	99,5 (99,2–99,8)	99,6 (99,3–99,8)
	Adultos	98,9 (98,4–99,4)	99,6 (99,5–99,8)	99,6 (99,4–99,9)	99,7 (99,5–99,9)	99,7 (99,5–99,9)
	Idosos	99,1 (98,6–99,7)	99,8 (99,6–99,9)	99,7 (99,6–99,9)	99,8 (99,6–100)	99,8 (99,7–99,9)
Vitamina E (mg)^c^						
	Adolescentes	81,0 (78,6–83,3)	93,8 (92,9–94,6)	89,5 (88,4–90,6)	92,5 (91,1–93,9)	90,9 (89,5–92,3)
	Adultos	86,1 (84,0–88,3)	96,1 (95,4–96,7)	92,8 (92,0–93,5)	95,3 (94,2–96,4)	94,2 (93,4–95,0)
	Idosos	90,7 (88,6–92,7)	97,6 (97,3–98,0)	95,3 (94,4–96,2)	97,2 (96,7–97,7)	96,6 (96,1–97,1)
Vitamina C (mg)						
	Adolescentes	34,8 (32,2–37,4)	41,2 (38,5–43,8)	30,1 (28,6–31,6)	25,4 (22,5–28,2)	27,9 (25,7–30,1)
	Adultos	46,7 (42,9–50,5)	52,9 (50,5–55,3)	40,9 (38,8–43,1)	35,7 (33,6–37,7)	39,7 (37,8–41,6)
	Idosos	48,5 (44,4–52,6)	55,1 (52,1–58,2)	42,3 (39,1–45,4)	37,7 (33,4–42,0)	42,0 (39,1–44,8)

a Equivalente de atividade de retinol (RAE).

b Ergocalciterol (D2) + colecalciterol (D3).

c Alfa-tocoferol total.

**Tabela 8:** Prevalências de inadequação (IC95%) da ingestão de vitaminas e minerais segundo região entre mulheres no Inquérito Nacional de Alimentação (INA/POF) 2008–2009.

Nutrientes	Faixa etária	Região				
		Norte	Nordeste	Sul	Sudeste	Centro-oeste
		% (IC95%)	% (IC95%)	% (IC95%)	% (IC95%)	% (IC95%)
Cálcio (mg)						
	Adolescentes	97,7 (97,3–98,2)	99,0 (98,7–99,2)	97,4 (97,1–97,7)	97,2 (96,7–97,8)	98,7 (98,4–99,0)
	Adultos	92,3 (91,7–92,9)	96,2 (95,8–96,7)	92,6 (91,3–93,8)	92,0 (90,5–93,4)	96,1 (95,6–96,6)
	Idosos	97,1 (96,4–97,8)	98,7 (98,5–98,9)	97,1 (96,3–97,9)	96,7 (95,9–97,5)	98,3 (98,0–98,6)
Magnésio (mg)						
	Adolescentes	54,2 (51,6–56,9)	64,1 (61,2–67,0)	54,2 (47,7–60,8)	58,8 (55,0–62,7)	56,6 (53,0–60,2)
	Adultos	62,4 (60,6–64,3)	71,0 (69,3–72,6)	59,9 (58,7–61,2)	65,4 (64,0–66,9)	63,1 (61,3–64,9)
	Idosos	67,5 (65,8–69,1)	75,3 (73,6–77,0)	64,9 (63,6–66,3)	69,5 (66,9–72,1)	67,8 (66,0–69,5)
Fósforo (mg)						
	Adolescentes	58,1 (55,9–60,2)	68,3 (66,7–69,8)	61,0 (59,3–62,7)	65,8 (62,8–68,7)	68,5 (66,5–70,4)
	Adultos	8,4 (7,8–9,0)	13,6 (12,8–14,4)	10,1 (9,2–11,0)	12,2 (10,9–13,5)	14,0 (13,1–15,0)
	Idosos	11,6 (10,7–12,5)	18,0 (16,4–19,5)	13,1 (12,3–13,8)	15,9 (15,0–16,8)	18,1 (16,9–19,2)
Cobre (mg)						
	Adolescentes	8,0 (6,9–9,1)	8,4 (7,4–9,4)	6,0 (5,1–6,8)	9,2 (7,0–11,4)	6,1 (4,8–7,4)
	Adultos	15,0 (13,5–16,6)	15,0 (13,9–16,1)	11,1 (10,0–12,2)	16,0 (13,6–18,3)	10,9 (9,6–12,2)
	Idosos	17,3 (15,3–19,3)	17,4 (16,2–18,5)	12,7 (11,5–14,0)	18,2 (15,9–20,6)	12,8 (11,3–14,3)
Zinco (mg)						
	Adolescentes	18,1 (15,4–20,8)	21,2 (18,8–23,6)	23,1 (20,7–25,4)	19,9 (15,2–24,7)	16,7 (14,2–19,1)
	Adultos	15,1 (13,3–16,9)	18,1 (17,3–18,8)	19,9 (19,1–20,7)	16,7 (13,1–20,4)	13,7 (12,2–15,1)
	Idosos	19,6 (16,1–23,1)	23,1 (19,9–26,3)	24,4 (21,7–27,2)	21,1 (17,3–24,9)	17,6 (14,7–20,4)
Vitamina A (mcg)^a^						
	Adolescentes	75,4 (71,6–79,2)	75,5 (71,3–79,7)	64,7 (61,1–68,3)	63,8 (59,2–68,5)	70,3 (66,1–74,5)
	Adultos	78,6 (75,5–81,7)	78,8 (76,2–81,4)	68,8 (66,3–71,3)	67,8 (64,1–71,4)	74,4 (71,3–77,4)
	Idosos	75,5 (71,5–79,4)	74,8 (72,1–77,6)	64,2 (61,9–66,6)	62,6 (59,3–65,9)	70,7 (67,7–73,7)
Tiamina (mg)						
	Adolescentes	51,4 (47,8–55,1)	49,9 (44,8–55,0)	36,6 (34,3–39,0)	32,8 (30,4–35,1)	51,2 (46,1–56,4)
	Adultos	73,0 (71,2–74,7)	71,2 (67,8–74,6)	58,9 (56,9–60,9)	54,5 (53,5–55,4)	72,3 (69,5–75,1)
	Idosos	80,4 (77,9–82,9)	77,9 (74,9–81,0)	66,5 (62,5–70,4)	61,9 (60,3–63,6)	79,1 (77,1–81,1)
Riboflavina (mg)						
	Adolescentes	34,4 (29,1–39,7)	30,2 (22,3–38,2)	23,7 (20,4–27,0)	21,6 (17,0–26,2)	33,1 (27,2–39,1)
	Adultos	45,6 (42,0–49,3)	40,8 (34,9–46,6)	33,5 (32,3–34,6)	30,6 (26,9–34,3)	43,7 (40,2–47,2)
	Idosos	46,5 (40,5–52,5)	41,7 (35,6–47,7)	33,7 (30,9–36,5)	31,1 (26,0–36,2)	44,4 (40,1–48,7)
Piridoxina (mg)						
	Adolescentes	76,9 (72,9–80,9)	76,2 (72,1–80,3)	67,6 (62,1–73,0)	63,2 (61,3–65,1)	78,3 (72,1–84,5)
	Adultos	92,4 (90,5–94,3)	92,1 (90,0–94,2)	88,2 (85,4–91,0)	85,5 (83,7–87,3)	93,5 (91,4–95,5)
	Idosos	98,0 (96,9–99,2)	97,6 (96,6–98,6)	96,0 (94,2–97,8)	94,7 (93,3–96,0)	98,0 (97,2–98,7)
Cobalamina (mcg)						
	Adolescentes	1,4 (0,5–2,2)	7,4 (4,0–10,8)	9,0 (4,9–13,0)	8,3 (3,7–12,9)	7,2 (4,0–10,3)
	Adultos	2,0 (1,3–2,6)	10,1 (8,7–11,5)	12,5 (10,3–14,8)	11,3 (8,3–14,3)	9,6 (8,2–11,0)
	Idosos	2,3 (1,6–3,1)	11,6 (9,8–13,3)	13,5 (11,8–15,3)	12,8 (10,7–14,9)	10,8 (9,0–12,7)
Vitamina D (mcg)^b^						
	Adolescentes	98,8 (98,5–99,2)	99,5 (99,4–99,7)	99,5 (99,3–99,7)	99,7 (99,5–99,8)	99,7 (99,6–99,8)
	Adultos	99,4 (99,1–99,7)	99,8 (99,7–99,9)	99,8 (99,7–99,9)	99,8 (99,7–99,9)	99,9 (99,8–99,9)
	Idosos	99,5 (99,2–99,8)	99,8 (99,7–100)	99,8 (99,6–100)	99,9 (99,8–100)	99,9 (99,7–100)
Vitamina E (mg)^c^						
	Adolescentes	87,0 (84,4–89,6)	96,2 (95,5–96,9)	93,4 (92,3–94,5)	95,5 (94,3–96,6)	94,5 (93,5–95,4)
	Adultos	95,0 (93,8–96,1)	99,0 (98,8–99,1)	97,9 (97,6–98,2)	98,6 (98,3–99,0)	98,5 (98,2–98,7)
	Idosos	95,9 (94,7–97,0)	99,1 (98,9–99,4)	98,2 (98,0–98,5)	98,9 (98,7–99,0)	98,5 (98,2–98,8)
Vitamina C (mg)						
	Adolescentes	30,8 (26,2–35,4)	36,9 (34,7–39,1)	26,1 (21,5–30,6)	21,5 (18,9–24,1)	25,4 (21,8–29,1)
	Adultos	37,1 (33,2–41,0)	43,4 (40,7–46,0)	31,9 (30,7–33,0)	26,5 (24,4–28,7)	30,6 (28,5–32,7)
	Idosos	37,4 (32,2–42,5)	43,9 (41,2–46,6)	31,9 (27,8–36,1)	26,7 (23,7–29,7)	30,9 (27,9–34,0)

a Equivalente de atividade de retinol (RAE).

b Ergocalciterol (D2) + colecalciterol (D3).

c Alfa-tocoferol total.

**Tabela 9:** Prevalências de inadequação (IC95%) da ingestão de vitaminas e minerais segundo região entre homens no Inquérito Nacional de Alimentação (INA/POF) 2017–2018.

Nutrientes	Faixa etária	Região				
		Norte	Nordeste	Sul	Sudeste	Centro-oeste
		% (IC95%)	% (IC95%)	% (IC95%)	% (IC95%)	% (IC95%)
Cálcio (mg)						
	Adolescentes	98,8 (98,5–99,1)	98,6 (98,4–98,9)	97,9 (97,4–98,3)	97,3 (96,9–97,6)	98,0 (97,7–98,4)
	Adultos	93,3 (92,1–94,5)	93,2 (92,3–94,0)	89,9 (88,6–91,3)	88,5 (87,4–89,7)	91,1 (90,5–91,7)
	Idosos	96,2 (94,8–97,5)	96,1 (95,0–97,2)	93,8 (93,4–94,3)	92,7 (91,3–94,1)	94,4 (93,4–95,4)
Magnésio (mg)						
	Adolescentes	58,4 (56,6–60,2)	55,9 (53,9–57,9)	54,0 (49,7–58,4)	53,4 (46,7–60,2)	43,7 (40,3–47,1)
	Adultos	74,4 (73,1–75,6)	71,1 (69,3–72,9)	70,2 (67,7–72,6)	65,8 (63,3–68,3)	58,9 (56,5–61,2)
	Idosos	84,8 (83,1–86,6)	82,3 (80,4–84,3)	81,3 (79,1–83,5)	77,8 (75,0–80,6)	71,8 (70,1–73,6)
Fósforo (mg)						
	Adolescentes	56,1 (54,3–58,0)	52,9 (50,4–55,3)	51,2 (48,0–54,3)	48,3 (46,5–50,2)	50,6 (45,3–55,8)
	Adultos	2,7 (2,0–3,4)	2,3 (1,7–2,8)	2,13 (2,0–2,3)	1,78 (1,3–2,3)	1,87 (1,1–2,7)
	Idosos	8,5 (7,0–10,0)	7,4 (6,1–8,7)	7,0 (6,6–7,3)	5,81 (5,0–6,7)	6,57 (4,8–8,3)
Cobre (mg)						
	Adolescentes	3,0 (2,6–3,4)	1,3 (1,2–1,4)	2,3 (1,8–2,8)	3,5 (2,7–4,4)	1,0 (0,8–1,2)
	Adultos	3,3 (2,7–3,9)	1,4 (1,2–1,7)	2,6 (2,2–3,0)	3,7 (3,1–4,3)	1,1 (0,8–1,4)
	Idosos	5,8 (4,6–7,1)	2,7 (2,2–3,2)	4,9 (4,3–5,4)	6,5 (5,8–7,2)	2,3 (1,9–2,8)
Zinco (mg)						
	Adolescentes	15,5 (12,3–18,8)	16,1 (12,8–19,3)	22,5 (20,3–24,8)	13,7 (10,6–16,8)	9,2 (6,6–11,8)
	Adultos	20,5 (16,1–2)	21,4 (19,1–23,6)	29,3 (27,1–31,5)	18,0 (17,2–18,9)	12,8 (10,8–14,9)
	Idosos	34,7 (28–41,4)	36,3 (33,3–39,2)	45,7 (44,5–46,8)	31,6 (30,1–33,1)	24,3 (21,7–26,9)
Vitamina A (mcg)^a^						
	Adolescentes	88,7 (86,2–91,1)	84,0 (81,2–86,8)	82,9 (81,4–84,4)	80,1 (76,9–83,3)	83,0 (77,3–88,7)
	Adultos	93,5 (92,6–94,5)	89,9 (88,8–91,1)	89,1 (87,9–90,4)	86,1 (84,9–87,3)	89,1 (84,8–93,5)
	Idosos	92,2 (90,6–93,8)	88,3 (86,1–90,5)	87,6 (86,1–89,2)	84,2 (82,4–86,0)	87,6 (83,4–91,8)
Tiamina (mg)						
	Adolescentes	45,2 (41,8–48,5)	34,1 (31,1–37,0)	29,1 (27,1–31,2)	22,4 (18,2–26,6)	33,6 (30,8–36,3)
	Adultos	67,2 (65,0–69,3)	55,2 (54,1–56,2)	50,2 (49,4–51,1)	39,3 (35,3–43,3)	55,7 (54,3–57,2)
	Idosos	78,2 (76,1–80,4)	67,4 (65,1–69,8)	63,2 (61,6–64,9)	51,4 (47,4–55,4)	67,6 (65,4–69,9)
Riboflavina (mg)						
	Adolescentes	45,8 (43,1–48,4)	37,2 (34,5–39,8)	32,8 (29,7–35,8)	34,6 (31,6–37,7)	42,3 (40,5–44,0)
	Adultos	65,6 (62,7–68,5)	56,1 (54,1–58,2)	51,9 (50,8–53,0)	51,7 (49,1–54,3)	62,4 (60,4–64,4)
	Idosos	68,7 (65,8–71,6)	59,1 (57,3–60,9)	55,7 (54,5–57,0)	54,6 (52,1–57,1)	65,7 (63,3–68,0)
Piridoxina (mg)						
	Adolescentes	73,5 (71,0–76,0)	69,5 (64,9–74,0)	66,8 (63,6–70,0)	65,1 (61,0–69,2)	75,7 (73,8–77,7)
	Adultos	85,7 (84,1–87,2)	82,8 (79,4–86,1)	81,6 (80,6–82,6)	78,7 (76,0–81,4)	87,5 (85,8–89,2)
	Idosos	96,7 (96,0–97,4)	95,5 (94,5–96,5)	94,8 (94,1–95,5)	93,3 (92,3–94,3)	97,1 (96,6–97,6)
Cobalamina (mcg)						
	Adolescentes	2,6 (2,2–3,0)	6,8 (5,3–8,3)	10,8 (9,4–12,1)	7,7 (5,4–10,0)	4,8 (4,0–5,6)
	Adultos	2,1 (1,5–2,7)	5,8 (4,4–7,2)	9,9 (9,0–10,9)	6,3 (4,9–7,6)	4,1 (3,3–5,0)
	Idosos	4,3 (2,8–5,8)	10,9 (8,0–13,8)	17,1 (15,8–18,5)	11,5 (9,6–13,4)	8,2 (6,7–9,8)
Vitamina D (mcg)^b^						
	Adolescentes	99,8 (99,7–99,9)	99,9 (99,8–99,9)	99,8 (99,7–99,9)	99,8 (99,8–99,9)	99,8 (99,7–99,9)
	Adultos	99,9 (99,8–99,9)	99,9 (99,9–100)	99,9 (99,9–100)	99,9 (99,8–99,9)	99,9 (99,8–100)
	Idosos	100 (99,9–100)	100 (100–100)	100 (99,9–100)	99,9 (99,9–100)	99,9 (99,9–100)
Vitamina E (mg)^c^						
	Adolescentes	81,8 (76,3–87,2)	92,8 (91,9–93,8)	88,0 (85,3–90,7)	90,6 (89,5–91,6)	87,4 (86,4–88,5)
	Adultos	85,3 (79,3–91,3)	94,7 (94,1–95,4)	90,7 (89,2–92,2)	92,7 (91,8–93,6)	90,0 (89,1–91,0)
	Idosos	91,2 (86,3–96,1)	97,3 (96,7–97,8)	94,7 (93,5–96,0)	95,7 (95,0–96,4)	94,2 (93,3–95,1)
Vitamina C (mg)						
	Adolescentes	42,7 (38,0–47,3)	36,2 (31,6–40,8)	33,9 (29,2–38,6)	25,3 (20,5–30,1)	32,8 (28,5–37,0)
	Adultos	57,2 (53,8–60,5)	50,0 (48,2–51,9)	48,0 (45,7–50,3)	36,1 (33,2–38,9)	46,6 (44,9–48,4)
	Idosos	58,8 (53,4–64,2)	51,4 (48,1–54,8)	50,1 (48,0–52,1)	37,6 (34,0–41,2)	48,6 (45,5–51,7)

a Equivalente de atividade de retinol (RAE).

b Ergocalciterol (D2) + colecalciterol (D3).

c Alfa-tocoferol total.

**Tabela 10:** Prevalências de inadequação (IC95%) da ingestão de vitaminas e minerais segundo região entre mulheres no Inquérito Nacional de Alimentação (INA/POF) 2017–2018.

Nutrientes	Faixa etária	Região				
		Norte	Nordeste	Sul	Sudeste	Centro-oeste
		% (IC95%)	% (IC95%)	% (IC95%)	% (IC95%)	% (IC95%)
Cálcio (mg)						
	Adolescentes	99,4 (99,0–99,8)	99,3 (98,8–99,7)	98,8 (98,4–99,1)	98,5 (97,8–99,3)	99,0 (98,4–99,6)
	Adultos	97,2 (96,5–97,8)	97,2 (96,5–97,8)	95,7 (95,5–95,9)	95,0 (94,2–95,8)	96,3 (95,6–96,9)
	Idosos	99,1 (98,8–99,4)	99,0 (98,9–99,1)	98,4 (98,2–98,5)	97,8 (97,5–98,1)	98,6 (98,4–98,8)
Magnésio (mg)						
	Adolescentes	68,9 (66,4–71,4)	65,5 (63,0–68,1)	66,0 (63,3–68,6)	61,2 (57,8–64,6)	54,7 (50,5–58,8)
	Adultos	74,0 (72,9–75,1)	70,6 (68,9–72,3)	69,7 (66,7–72,6)	65,6 (62,7–68,6)	58,5 (55,4–61,6)
	Idosos	77,0 (74,2–79,8)	74,8 (72,4–77,3)	74,0 (72,9–75,2)	69,3 (67,0–71,5)	62,8 (58,7–66,9)
Fósforo (mg)						
	Adolescentes	75,0 (71,0–79,0)	72,4 (68,0–76,8)	70,7 (65,8–75,6)	67,8 (64,0–71,6)	69,0 (63,0–74,9)
	Adultos	14,7 (13,4–15,9)	13,3 (12,1–14,6)	12,8 (11,7–13,9)	11,2 (10,2–12,3)	11,7 (8,6–14,8)
	Idosos	21,6 (19,2–24,1)	19,5 (16,9–22,1)	18,8 (17,9–19,6)	16,5 (14,6–18,4)	17,6 (13,6–21,7)
Cobre (mg)						
	Adolescentes	8,6 (7,5–9,7)	4,6 (4,3–4,9)	7,2 (6,0–8,3)	9,8 (8,6–11,1)	3,8 (3,2–4,3)
	Adultos	13,3 (12,0–14,6)	7,3 (6,6–8,1)	11,5 (10,8–12,1)	15,1 (13,7–16,4)	6,2 (5,3–7,1)
	Idosos	14,3 (11,8–16,9)	8,3 (6,7–9,9)	12,6 (11,0–14,2)	16,3 (14,2–18,3)	6,9 (5,4–8,4)
Zinco (mg)						
	Adolescentes	22,9 (18,7–27,1)	23,6 (20,5–26,8)	31,8 (28,8–34,9)	20,3 (19,1–21,4)	14,5 (12,0–17,0)
	Adultos	18,8 (14,3–23,2)	20,3 (18,0–22,6)	27,8 (25,6–29,9)	17,2 (16,3–18,1)	12,0 (9,8–14,2)
	Idosos	27,3 (22,4–32,2)	28,6 (25,8–31,4)	37,5 (34,5–40,5)	24,4 (22,7–26,0)	18,3 (15,8–20,9)
Vitamina A (mcg)^a^						
	Adolescentes	85,3 (81,5–89,2)	79,4 (75,0–83,7)	78,4 (76,1–80,6)	73,5 (68,2–78,7)	77,8 (69,6–86,0)
	Adultos	86,9 (85,9–87,8)	80,8 (79,6–82,1)	79,9 (77,2–82,5)	75,6 (74,2–77,1)	79,9 (74,4–85,3)
	Idosos	80,0 (77,5–82,5)	73,3 (71,1–75,5)	72,6 (71,3–74,0)	66,8 (64,5–69,1)	72,2 (65,0–79,4)
Tiamina (mg)						
	Adolescentes	54,2 (51,0–57,5)	42,8 (40,5–45,1)	38,6 (36,8–40,3)	29,2 (25,0–33,3)	42,9 (41,2–44,5)
	Adultos	75,8 (73,7–77,9)	65,2 (63,3–67,1)	60,6 (59,7–61,4)	49,7 (45,8–53,6)	65,7 (64,4–67,0)
	Idosos	79,5 (77,3–81,8)	70,5 (68,7–72,2)	66,2 (65,6–66,9)	54,5 (51,4–57,7)	70,5 (69,2–71,9)
Riboflavina (mg)						
	Adolescentes	50,4 (44,5–56,4)	40,8 (35,8–45,8)	36,9 (30,2–43,5)	36,6 (31,1–42,1)	46,6 (41,6–51,6)
	Adultos	62,2 (59,7–64,7)	52,6 (51,5–53,6)	48,2 (46,0–50,4)	48,3 (46,4–50,2)	59,2 (58,0–60,4)
	Idosos	57,9 (53,9–61,9)	47,8 (46,0–49,6)	43,9 (41,7–46,2)	43,3 (42,0–44,6)	54,6 (52,5–56,7)
Piridoxina (mg)						
	Adolescentes	79,9 (76,4–83,4)	76,0 (72,4–79,6)	74,7 (71,4–78,1)	70,9 (66,5–75,3)	81,1 (78,2–83,9)
	Adultos	93,9 (93,2–94,6)	92,2 (90,7–93,7)	91,4 (90,9–91,9)	89,6 (88,3–90,9)	94,8 (94,3–95,2)
	Idosos	97,9 (97,3–98,4)	97,0 (96,2–97,9)	96,6 (96,2–97,1)	95,4 (94,7–96,2)	98,2 (97,8–98,5)
Cobalamina (mcg)						
	Adolescentes	4,2 (1,8–6,6)	10,0 (5,5–14,5)	15,7 (10,8–20,7)	11,0 (6,5–15,5)	7,4 (4,0–10,9)
	Adultos	6,1 (4,6–7,6)	14,8 (11,8–17,8)	22,2 (21,0–23,5)	15,9 (13,5–18,3)	11,1 (9,2–13,1)
	Idosos	7,9 (5,7–10,0)	17,8 (13,5–22,1)	26,3 (23,7–28,8)	18,8 (14,9–22,7)	14,0 (10,6–17,3)
Vitamina D (mcg)^b^						
	Adolescentes	99,8 (99,7–100)	99,9 (99,9–99,9)	99,9 (99,9–100)	99,8 (99,8–99,9)	99,9 (99,8–99,9)
	Adultos	100 (99,9–100)	100 (100–100)	100 (99,9–100)	99,9 (99,9–100)	99,9 (99,9–100)
	Idosos	100 (100–100)	100 (100–100)	100 (100–100)	100 (99,9–100)	100 (99,9–100)
Vitamina E (mg)^c^						
	Adolescentes	89,1 (83,8–94,5)	96,0 (95,1–97,0)	93,4 (91,5–95,3)	94,8 (93,5–9,06)	92,3 (91,0–93,6)
	Adultos	94,1 (91,0–97,2)	98,3 (98,0–98,7)	96,6 (95,6–97,6)	97,5 (97,0–97,9)	96,3 (95,8–96,9)
	Idosos	95,9 (92,7–99,2)	98,9 (98,6–99,2)	97,9 (97,2–98,6)	98,3 (97,9–98,6)	97,5 (97,0–97,9)
Vitamina C (mg)						
	Adolescentes	37,5 (32,3–42,7)	30,9 (26,1–35,8)	29,2 (25,5–32,9)	20,0 (17,1–22,9)	28,0 (24,7–31,4)
	Adultos	48,0 (44,0–51,9)	40,8 (38,5–43,1)	38,9 (37,0–40,8)	27,9 (25,4–30,5)	37,6 (35,6–39,6)
	Idosos	45,2 (41,0–49,4)	38,4 (36,0–40,9)	36,6 (33,4–39,8)	25,5 (21,7–29,2)	35,2 (32,7–37,7)

a Equivalente de atividade de retinol (RAE).

b Ergocalciterol (D2) + colecalciterol (D3).

c Alfa-tocoferol total.

**Figura 1:** Prevalências de inadequação (IC95%) da ingestão de vitaminas e minerais segundo região no Inquérito Nacional de Alimentação (INA/POF) 2017–2018.

**Figura 2:** Prevalências de inadequação (IC95%) da ingestão de vitaminas e minerais segundo região no Inquérito Nacional de Alimentação (INA/POF) 2008–2009.

Discrepâncias foram encontradas nas prevalências de inadequação de tiamina entre Norte e Sudeste e de zinco entre Sul e Centro-Oeste, cujas estimativas, em todas as faixas etárias, foram de 1,5 a 2 vezes maiores na primeira região em relação à última, respectivamente. A região Sudeste se destacou com menores prevalências de inadequação de tiamina em relação às regiões Nordeste e Centro-Oeste entre adultos e idosos, ao passo que o Sul, na segunda posição de menor inadequação, ainda diferiu em relação à região Norte entre adolescentes e adultos. Além disso, a região Norte despontou com as diferenças mais elevadas nas prevalências de inadequação de vitamina C, em contraste com a região Sudeste, entre homens e mulheres de todas as faixas etárias, e de magnésio, em contraste com a região Centro-Oeste, para adultos, mas com prevalência menor de inadequação de cobalamina em relação à região Sul entre mulheres adultas e idosas (Tabelas 9 e 10).

## DISCUSSÃO

Apesar da distância de uma década entre os dois Inquéritos Nacionais de Alimentação, o consumo de energia não variou de forma significativa no período, e os nutrientes que causavam preocupação no primeiro inquérito permanecem sendo aqueles com maiores prevalências de inadequação na segunda investigação, com destaque para cálcio, vitaminas A, D e E, magnésio e piridoxina. Ressalta-se também a alta prevalência de consumo excessivo de sódio nos dois inquéritos, principalmente entre homens adultos.

Os indivíduos mais pobres foram mais vulneráveis ao consumo inadequado da maioria dos nutrientes investigados, com atenção especial para vitaminas A e C, tiamina e riboflavina, que tiveram grande diferença, absoluta e relativa, em comparação com o estrato de maior de renda. Foram observadas disparidades demográficas de acordo com as regiões do país e, de forma geral, as prevalências de inadequação de consumo foram maiores para a região Norte, seguida das regiões Nordeste ou Centro-Oeste.

As prevalências de inadequação aqui apresentadas têm como referência valores ideais para a manutenção de marcadores bioquímicos em indivíduos saudáveis. Elas não indicam, portanto, necessariamente, o aparecimento de desfechos clínicos de saúde. Esses desfechos decorrem de mecanismos metabólicos que envolvem a interação entre a biodisponibilidade e a necessidade de nutrientes, assim como de características individuais relacionadas, por exemplo, a fatores genéticos e ao estado nutricional ao longo da vida^18^.

Poucos estudos com amostra representativa da população brasileira analisaram a prevalência de inadequação da ingestão de nutrientes. Dentre eles, o Estudo de Riscos Cardiovasculares em Adolescentes (Erica)^19^ avaliou adolescentes brasileiros e apontou como nutrientes com maiores prevalências de inadequação os mesmos observados na presente análise, por exemplo o cálcio (prevalência de inadequação de 99%), vitaminas A (prevalências de inadequação variando entre 60% e 74%) e E (prevalência de inadequação de 100%) e sódio (prevalências de consumo excessivo variando entre 79% e 91%).

A inadequação de 50% na ingestão de fósforo entre os adolescentes investigados na presente análise também foi observada no estudo Erica, que encontrou prevalências de inadequação ainda mais altas, entre 59% e 65%. A maior inadequação de fósforo nesta faixa etária se deve ao maior valor de necessidade média de ingestão, aproximadamente o dobro do recomendado para adultos^20^. Destaca-se ainda a importância do fósforo na adolescência, juntamente com o cálcio e a vitamina D, para manutenção adequada do metabolismo ósseo e prevenção de doenças como escoliose e osteoporose em idades mais avançadas^21^.

Estudos em países da Europa, América do Norte, América Latina, África e Ásia também apontam para a ingestão inadequada de nutrientes em diversas populações. Cada região apresenta características específicas em relação aos nutrientes cuja ingestão por sua população é mais crítica. Contudo, em geral, observaram-se altas prevalências de inadequação na ingestão de cálcio, vitaminas A, D, E, zinco, folato e ferro^22,23^.

A evolução da inadequação de nutrientes foi avaliada também com dados de adultos do inquérito norte-americano *National Health and Nutrition Examination Survey*, de 2003 a 2016. Foi observada redução da inadequação para a maioria dos nutrientes. Ainda assim, cálcio, magnésio, vitaminas A, C, D, e E e folato permaneceram com inadequação acima de 50% em 2015–2016^24^.

Cabe destacar, porém, que apesar de diferentes estudos apontarem altas prevalências de inadequação para os mesmos nutrientes, tais resultados precisam ser vistos com cautela, uma vez que as pesquisas diferem nos métodos de avaliação dietética, nos valores de referência e nas análises utilizadas para estimar o consumo usual.

Alguns aspectos importantes sobre a alta prevalência de inadequação da ingestão de cálcio e vitamina D precisam ser apontados. Os valores de referência desses dois nutrientes foram revistos na última década, com base na melhor evidência disponível à época^20^. No entanto, assumiu-se, para estabelecer esses valores, pouca ou nenhuma exposição solar^20^, o que pode não ser aplicável em países de clima predominantemente tropical, como o Brasil. Parte da necessidade de vitamina D pode ser atendida por síntese a partir da exposição solar. Assim, em estudo realizado no município de São Paulo, quase 100% da população tinha ingestão inadequada de vitamina D, mas aproximadamente metade das pessoas apresentavam nível sérico adequado^25^.

As necessidades de cálcio também são fonte de debate intenso, que envolve tanto a adequação dos estudos de balanço quanto a não confirmação dos efeitos benéficos para saúde óssea em metanálises de estudos longitudinais^26^. No entanto, apesar das críticas aos valores de referência, é importante salientar que a frequência do consumo de alimentos fonte de cálcio e vitamina D, como laticínios e pescados, é muito baixa desde o INA 2008–2009^10^.

O consumo excessivo e inadequado de sódio se manteve entre os dois inquéritos, resultado alinhado com a Pesquisa Nacional de Saúde de 2013, que estimou o consumo de sal na população brasileira pela excreção urinária de sódio. O consumo verificado, maior entre os homens, foi o dobro da quantidade máxima de ingestão recomendada pela Organização Mundial da Saúde^27^.

A alta prevalência do consumo excessivo de sódio é uma preocupação de décadas no Brasil^28^ e no mundo^29^. O seu consumo em excesso, associado a uma dieta pobre em potássio, aumenta os níveis pressóricos e representa risco para o desenvolvimento da hipertensão arterial, que por sua vez tem sido elencada como um dos fatores de risco mais importantes para morbimortalidade por doenças cardiovasculares^30^. Outro resultado de destaque foi o aumento, entre idosos, da inadequação de riboflavina, vitamina que pode exercer efeitos neuroprotetores para alguns distúrbios neurológicos (por exemplo, doença de Parkinson, enxaqueca e esclerose múltipla), tendo como principais fontes o leite e produtos lácteos, vísceras e carne bovina^31^.

Indivíduos com menor renda domiciliar per capita foram aqueles com maiores prevalências de inadequação para a maioria dos nutrientes investigados. De forma similar, em geral, regiões mais pobres do país apresentaram maiores prevalências de inadequação. Estudos com a população norte-americana também mostraram que, entre adultos de baixo nível socioeconômico, aumentava a proporção de indivíduos com ingestão inadequada de nutrientes^24^, enquanto nos maiores níveis de renda o risco de inadequação diminuía^32^.

As disparidades socioeconômicas e demográficas aqui apontadas já foram observadas em outras análises do inquérito de 2008–2009^33^ e em outras amostras de base populacional brasileira^34^. Essas disparidades também foram observadas na avaliação do consumo de alimentos usando a mesma base de dados, verificando-se redução na frequência do consumo de frutas entre 2008–2009 e 2017–2018, mais acentuada no quarto de renda mais baixo. Em contrapartida, os resultados de 2017–2018 apontaram que alimentos tipicamente brasileiros, como arroz, feijão, farinha de mandioca, milho e alimentos à base de milho foram consumidos com mais frequência entre indivíduos de menor renda^10^.

Diferenças regionais acompanham as diferenças de renda. Alimentos como farinha de mandioca, milho e alimentos à base de milho foram mais frequentemente consumidos em regiões mais pobres, como Norte e Nordeste, respectivamente. Por outro lado, as regiões Sul e Sudeste apresentaram as maiores médias de consumo per capita de frutas e verduras^10^. Destaca-se ainda que os resultados da POF 2017-–2018 mostraram também como o acesso regular e permanente dos domicílios a alimentos de qualidade e em quantidade suficiente é desigual no Brasil. As regiões Norte e Nordeste apresentaram as menores proporções de domicílios particulares em situação de segurança alimentar. Menos da metade dos moradores dessas regiões tiveram acesso pleno e regular aos alimentos^5^. Portanto, qualquer estratégia que objetive melhorar a ingestão de nutrientes no Brasil precisa enfatizar o acesso à alimentação adequada aos indivíduos mais desfavorecidos e residentes nas regiões mais pobres.

O presente estudo apresenta algumas limitações. Primeiro, a ingestão de nutrientes não considerou as informações sobre uso de suplementos nutricionais aplicadas no último inquérito. Os indivíduos foram questionados somente sobre o uso nos últimos 30 dias, mas sem informações sobre quantidade e frequência consumida. Logo, não foi possível estimar o quantitativo de nutrientes fornecidos por suplementos. O uso de multivitamínicos, vitaminas do complexo B e vitamina C foi relatado por 11,1% da população, variando até 19,5% em idosas, e o uso de suplemento de cálcio foi relatado por 4,6%, variando até 21,3% também em idosas.

A segunda limitação foi a mudança no método de coleta do consumo alimentar, que dificulta a comparação entre os dois inquéritos. A mudança se deu porque estudos de validação sistematicamente mostraram que a coleta por meio de R24h assistida por computador diminui o sub-relato de energia, sódio, potássio e proteína^35^. Assim, acredita-se que a alteração tenha permitido estimativas mais acuradas. Entretanto, o sub-relato na ingestão energética, comum na coleta por R24h, provavelmente levou a prevalências de inadequação superestimadas. Mas como a ingestão calórica foi semelhante entre os dois inquéritos (1.753 kcal em 2008–2009 e 1.748 kcal em 2017–2018), é provável que o efeito desse possível sub-relato tenha sido semelhante.

As elevadas e persistentes prevalências de inadequação verificadas neste estudo apontam para uma alimentação nutricionalmente deficiente, em um contexto de sindemia global de obesidade, desnutrição e mudanças climáticas^36^. Tendo em vista esse cenário, a FAO reconhece o desafio de combater a má nutrição em todas as suas formas, incluindo a desnutrição, carências nutricionais específicas, sobrepeso, obesidade e DCNT associadas à alimentação^37^.

No Brasil, o acesso à alimentação adequada como direito está previsto em lei^38^ e incorporado à Constituição Federal^39^. Nessa perspectiva, o acesso físico e econômico aos alimentos deve garantir não somente alimentação em quantidade suficiente como nutricionalmente adequada. No entanto, como demonstrou o presente artigo, a ingestão de nutrientes tem se mostrado inadequada entre os brasileiros.

## CONCLUSÃO

Prevalências elevadas de inadequação de ingestão de nutrientes e consumo excessivo de sódio foram verificadas em ambos os inquéritos. A prevalência de inadequação se modifica de acordo com as variáveis sociodemográficas, aumentando entre indivíduos nos menores estratos de renda e nas regiões mais pobres do país.
